# Interactive narratives reveal the personalizing effect of agency on episodic memory

**DOI:** 10.1038/s41467-026-73907-2

**Published:** 2026-06-06

**Authors:** Xian Li, Nicole Kim Ni, Savannah J. Born, Ria J. Gualano, Iris Lee, Buddhika Bellana, Janice Chen

**Affiliations:** 1 Department of Psychological and Brain Sciences, Johns Hopkins University, Baltimore, MD USA; 2 Department of Psychological and Brain Sciences, Washington University in St. Louis, St. Louis, MO USA; 3 Department of Communication, Cornell University, Ithaca, NY USA; 4 The Writing Seminars, Johns Hopkins University, Baltimore, MD USA; 5 Department of Psychology, Glendon College, York University, Toronto, ON Canada

**Keywords:** Human behaviour, Long-term memory, Motivation

## Abstract

Humans make choices that actively shape the trajectory of events in their lives. These choices rely on personal knowledge that can influence how these events are later remembered. We study how people’s memory for a naturalistic sequence of events is altered when their choices control the future. Participants read “choose-your-own-adventure” stories with full, partial, or no control over future events. In all conditions, events which are causally or semantically central to the story are better recalled. However, even when all participants read the exact same story, those with full control recall more idiosyncratic combinations of events. Moreover, their memories are less well predicted by generic sentence embeddings, suggesting a shift away from normative semantic space. Agency also increases the likelihood of jointly remembering or forgetting consecutive events. These results reveal that agency fundamentally reshapes memory organization, increasing the influence of idiosyncratic personal factors and strengthening local temporal integration.

## Abstract (web summary)

Humans often make choices that shape the events they experience. Here, the authors show that agency during narratives reorganizes event memory, producing more personalized recall and stronger links between nearby moments.

## Introduction

We exert control over how our lives unfold. This capacity, known as *agency*, is an essential aspect of how individuals interact with the world^1–6^ and how they remember it^7–10^. Imagine how a person might recall their evening activities: “I arrived home from work and decided to make lasagna for dinner, but realized I was out of cheese, so I drove to the grocery store.” This recollection reflects how actions (driving) stem from internal states (hunger) and decisions (making dinner), influenced by personal preferences (lasagna). Our memories often involve such sequences of connected events, and we typically take an active role in determining their trajectory. Yet, surprisingly little is known about how agentive narratives like these are encoded and recalled.

What is known about the role of agency in memory? When people have some level of control over the items that they learn about, they tend to remember more^7,10–17^. While compelling, these findings come largely from studies using lists of isolated and unrelated words, images, or videos as memoranda^13,18^. Life events, however, do not occur in isolation, nor are they remembered in isolation. Naturalistic experiences unfold as sequences of events, assembling into rich semantic and causal structures that shape later memory^19–25^. As a result, the influence of agency in naturalistic contexts may extend beyond a mere increase in the amount of information remembered. Indeed, active control has been shown to enhance memory for specific aspects of an experience, such as its spatial^8,26^, temporal^18^, or causal structure^27,28^. Such studies point to the possibility that agency affects which across-event relations we attend to during encoding – potentially changing precisely *what* we remember about an experience rather than *how much*^29^.

To illustrate this idea, consider how semantic relations shape episodic memory. Semantic similarity influences both the order and probability of recall, such that semantically related items tend to be retrieved consecutively in free recall tasks^30–32^. Extending this to event memory, Lee & Chen^21^ showed that events that occupied more central positions (i.e., had stronger or more numerous connections) within a story’s semantic network were more likely to be recalled. However, in contexts with agency—such as a choose-your-own-adventure narrative—the semantic associations that come to mind during a given event can vary substantially from person to person. For example, chocolate ice cream is often considered a tasty dessert, but if *I* had recently chosen to poison a batch as a part of an elaborate plot to take down an enemy, it now takes on particular associations for *me*. This choice would change my semantic associations with “chocolate ice cream”, pulling it away from its usual position in generic semantic space towards “poison” instead, and altering how story events featuring chocolate ice cream relate to other events^33,34^. In this way, agency may highlight an individual’s idiosyncratic experiences and knowledge, causing memory to rely less on the kinds of generic semantic relations typically derived from large corpora^33,35–37^ and more on personalized semantic associations unique to each individual.

The extent to which agency changes *what* or *how much* we remember, particularly in the context of richly interconnected naturalistic events, remains unclear. In traditional paradigms, agency has been shown to boost the quantity of remembered details, possibly because making choices is inherently rewarding^38,39^ and such motivational factors can enhance memory encoding^40^. Additionally, agency may increase the self-relevance of the current input^11^, which could boost episodic memory by encouraging integration of the input with existing networks of self-relevant knowledge – a form of deeper elaborative processing^41–44^. What do these ideas suggest about how agency might alter memory for sequences of interconnected events? Since making choices requires agency, one may predict enhanced memory for events that involve making a choice. Furthermore, since agency activates self-relevant knowledge, it could amplify *inter-individual differences* in memory. For example, two friends exploring the main street of a new city together might visit shops together in the same sequence, but have different motivations for doing so (e.g., historical vs. food tour). The two friends would experience distinct framings for how the shops relate to one another; two “personalized” networks of associations across time would be generated, making different shops memorable or forgettable for each person^45^. In this way, agency need not increase the amount of information remembered wholesale, but could instead shift *which* relations among events are formed or attended to in the process of organizing them. This idea that agency might personalize individuals’ memory has yet to be tested.

In this study, we examined how agency affects memory for complex event sequences by having participants read interactive narratives, i.e., “choose-your-own-adventure” stories. Every few sentences, participants were offered a choice about how the story would proceed. In the Free condition, these choices were always granted (full agency); in the Yoked condition, choices were not always granted (partial agency); in the Passive condition, choices were not offered (no agency). While our main comparisons centered on full versus no agency, the Yoked condition provided a meaningful middle ground, enabling us to investigate the effects of degree of control and denied choices on recall. After reading, participants freely recalled the story. Critically, half of the events in a narrative were designed to be identical across participants in all conditions. If agency personalizes memory, we should see more inter-individual variability in the contents of recall for participants in the Free condition as compared to the Yoked or Passive conditions, despite the identical story content at encoding.

Our results reveal that agency indeed reshapes the way in which narrative events are recalled. Participants in the Free condition remembered more divergent subsets of events from each other relative to the Yoked and Passive participants, even when all events were identical across conditions; in other words, agency personalized memory. Furthermore, generic semantic associations between events (estimated via sentence embeddings^35^) were worse at predicting memory in the Free condition, consistent with the personalizing influence of agency on memory. Meanwhile, the impact of causal connections on memory was not changed by agency. Finally, in the Free condition, temporally adjacent events at encoding were more likely to share the same mnemonic fate (either both remembered, or both forgotten), which we term the *neighbor encoding effect*. Overall, the results show that the introduction of agentive control changes fundamental organizing principles of episodic memory for naturalistic events, particularly in terms of how semantic and temporal associations between events shape what is subsequently remembered. We speculate that the activation of self-relevant knowledge needed to make decisions drives this change, resulting in the emergence of more personalized memories.

## Results

**Fig. 1:**
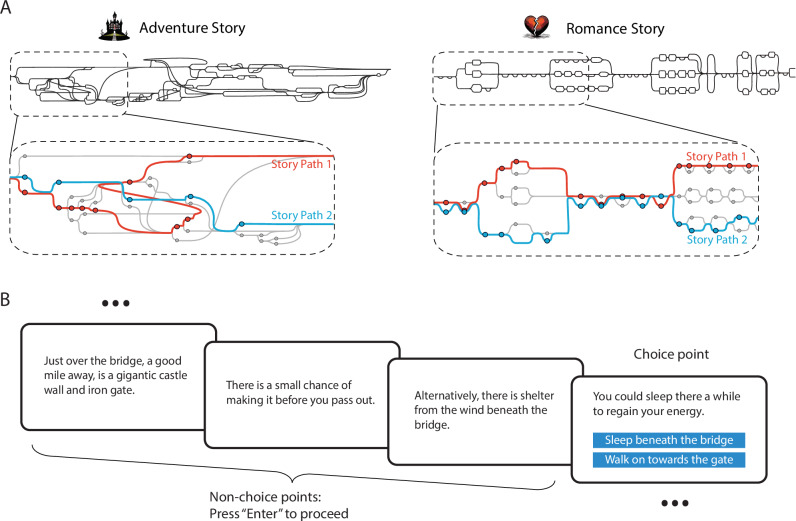
The choose-your-own-adventure (CYOA) story tree diagram and experiment paradigm. **A** Diagrams illustrating all possible story paths for the Adventure (left) and *Romance* stories (right). The story begins at the leftmost point of the tree (“Start”) and proceeds to the right; each line-split represents a choice-point, where options are offered, and each node (circles in popout) represents a choice option. Thus, an individual story can be visualized as a path through the tree, e.g., one participant chose the red nodes (choice options) while reading, resulting in Story Path 1, whereas another participant chose the blue nodes while reading, resulting in Story Path 2. The Romance story was designed to have half of its events shared across all participants, as seen in central sections of the tree where all lines merge. **B** Schematic of the CYOA paradigm with self-paced reading. Sentences were presented in black text on a white screen. Participants hit ‘Enter’ to proceed to the next sentence until arriving at a choice point. At a choice point, options were listed below the story text, and participants indicated their choice using the mouse.

We conducted the experiment with two different interactive stories, *Adventure* and *Romance*. In all conditions, participants read the story in a self-paced manner, one sentence at a time. In the Free condition (*Adventure:* *N* = 22; *Romance*: *N* = 100), the story periodically came to a “choice point” at which participants were offered 2–4 options for what could happen next. Participants indicated their preferred option, and the story proceeded following the plotline of their choice. Thus, each Free participant generated a unique “story path” through the narrative tree (Fig. 1). In the Yoked condition (*Adventure*: *N* = 45; *Romance*: *N* = 53), participants encountered the same choice points and options, and made their selections, but these selections were ignored. They were instead obliged to follow the story path of a previously collected Free participant. Consequently, Yoked participants sometimes did have their choice granted and sometimes did not, depending on whether they happened to make the same choice as the Free participants to whom they were yoked. Yoked participants were told in advance that they might not always have their choices granted, but they were not informed about the existence of Free participants. In the Passive condition (*Adventure*: *N* = 49; *Romance*: *N* = 55), participants encountered the same choice points but did not get to choose; these participants also followed the story path of a previously collected Free participant. Thus, the three conditions were designed to give participants three different levels of agency: Free had the most perceived control over the story, Yoked had an intermediate amount of perceived control (i.e., choices were sometimes granted by chance), and Passive had the least. For the *Adventure* story, each of the 22 story paths generated by Free participants was read by 2-3 Yoked and 2-3 Passive participants. For the *Romance* story, 18 of the original 100 Free participants were selected semi-randomly, and 2-3 Yoked and 2-3 Passive participants read each of these 18 story paths (see Methods). There were minor differences in the data collection procedures for the two stories; see Methods for details. The *Romance* story had an additional design feature: 50% of events were identical across all participants (Fig. 1).

Overall, this design allowed participants to explore a naturalistic choose-your-own-adventure narrative with varying degrees of agency. Critically, variability in story paths was fully matched across conditions: each unique path generated by a Free participant was read *verbatim* by multiple Yoked and Passive participants, including the same physical action of advancing screens (e.g., mouse clicks). Thus, potential differences in memory across conditions cannot be attributed to any stimulus variation other than the degree of perceived agency.

After story reading, all participants were asked to recall the story in as much detail as possible, by typing in a customized text box (see Methods). For the *Romance* story only, a multiple-choice recognition test was administered, as well as a number of trait surveys. All data were collected on Prolific. Each story path was segmented into “events” of a few sentences (see Methods) for subsequent analyses. Recall was scored for accuracy, and semantic and causal relations between story events were calculated or determined via human judgment. See Supplement S1 and Supplementary Fig. S1 for information about a GPT-based method for identifying causal relations in narratives. Responses for a third story were collected but not reported here due to poor data quality; see Supplement S2 for details.

### Agency had no statistically significant effect on overall recall performance

For each participant, events were binned according to whether they were remembered or forgotten. Independent raters compared each sentence of recall to the story path read by the participant; if any part of a given event was mentioned in any recall sentence, it was counted as remembered. There were no statistically significant differences in recall performance across conditions in either story (*Adventure:* F(2,113) = 1.43, *p* = 0.243, η^2^ = 0.025; *Romance:* F(2,123) = 0.67, *p* = 0.513, η^2^ = 0.011; Supplementary Fig. S2). The *Romance* story additionally recorded individual reading speed and showed no statistically significant difference across the three agency conditions (reading time per sentence: F(2,123) = 0.41, *p* = 0.668, η^2^ = 0.007; total reading time: F(2,123) = 0.42, *p* = 0.656, η^2^ = 0.007; see Supplement S3 for details). See Supplement S4 for details about memory for choice and non-choice events; See Supplement S5 for details about recognition memory performance; See Supplement S6 for details about memory for denied and granted choice events. Overall, increased agency did not result in a statistically significant effect on the number of remembered details, unlike previous work using random-item list-learning paradigms^7,10–17^; instead, this result adds to the set of studies showing that agency’s enhancing effects on memory depend on details of the content, experimental paradigm, and population^46–48^.

### Agency magnified individual variability in recall and choice

The *Romance* story, by design, had half of its events shared across all participants (“shared story sections”), regardless of condition (Fig. 1A). While participants made many choices during these shared story sections, unbeknownst to them, all choice options led to the same subsequent events. This allowed us to examine inter-participant variability in terms of memory (which events were recalled) and choice behavior (which options were selected) when all events were perfectly matched across participants, i.e., all participants read these events, and the events were composed of identical text.

#### Individual variability in recalled events

**Fig. 2:**
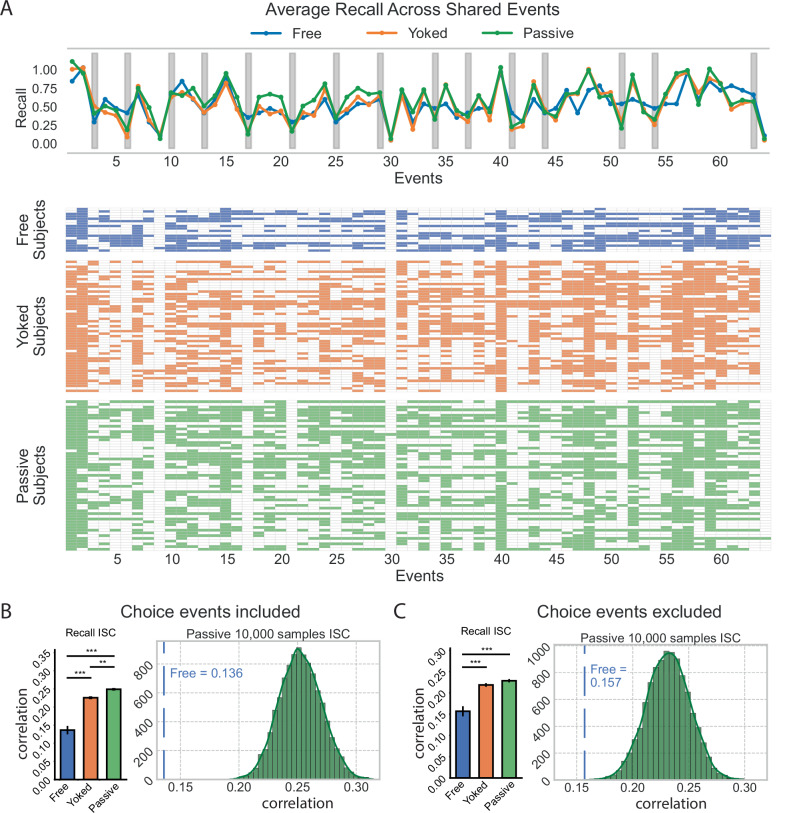
Recall inter-subject correlation (ISC) for the “shared story sections”. **A** *Top:* Average recall across all participants in each condition (Free, Yoked, Passive) for the 64 events in the shared story sections of the Romance story; the 15 choice events are indicated by grey bars. *Bottom:* Raster plot showing the raw data for each participant’s vector of recall performance (shaded = remembered, blank = forgotten). Note that choice events are qualitatively different from non-choice events in the current paradigm by design and therefore cannot be directly compared against each other. **B** *Left:* Average within-condition Recall ISC for the 64 shared events. *Right:* Distribution plot from the permutation test comparing the true Recall ISC for the Free condition (blue dashed line) to 10,000 Recall ISC values from samples drawn from the Passive condition, where each Passive sample consisted of 18 unique story paths (green distribution). **C** Same as B with Choice events excluded. The bar plots in B-C compared the group averaged recall ISC between all pairs of subjects in the Free (*n* = 153), Yoked (*n* = 1378), and Passive (*n* = 1485) conditions. Two-sided *t*-tests to follow up on the one-way ANOVAs for both stories showed that Free was lower than Yoked (ps *< *0.001) and Passive (ps < 0.001) conditions. Data are presented as mean values +/− SEM. The distribution plots in B-C demonstrate the permutation test results when controlling for the sample size and story versions to be the matched across conditions (*n *= 153).

A recall score (0 = Forgotten, 1 = Recalled; see Methods for details) for each of the 64 events in the shared story sections was extracted for each participant, composing a vector of recall performance (Fig. 2A). To assess the memory similarity across participants, we computed the inter-subject correlation (ISC), i.e., the Pearson correlation between each pair of participants’ recall performance vectors, i.e., “Recall ISC”. While events differed in their overall memorability, Recall ISC was significantly above zero in all three conditions (*Romance:* Free: mean *r* = 0.136, 95% CI [0.113, 0.159], t(152) = 11.75, d = 0.95, *p* < 0.001; Yoked: mean *r *= 0.226, 95% CI [0.218, 0.233], t(1377) = 58.29, d = 1.57, *p *< 0.001; Passive: mean *r *= 0.249, 95% CI [0.242, 0.256], t(1484) = 70.87, d = 1.84, *p* < 0.001; one-sample t-tests against zero), indicating that individuals tended to remember events more similarly to one another than would be expected by chance.

Interestingly, when comparing across the conditions, Free participants had reduced Recall ISC relative to Yoked and Passive participants, indicating that agency induced greater individual variability in terms of which events were recalled (Fig. 2B, left; *Romance:* F(2,3013) = 48.13, *p* < 0.001, η^2^ = 0.031; post-hoc tests: Free vs. Yoked: t(1529) = −7.29, *p* < 0.001, d = −0.62, 95% CI for mean difference [−0.113, −0.065]; Free vs. Passive: t(1636) = −9.73, *p* < 0.001, d = −0.83, 95% CI for mean difference [−0.135, −0.090]). Out of the 64 events, 15 were “choice events” (the event that the participant chose to occur, e.g., “Sleep beneath the bridge” in Fig. 1B). To examine whether the reduced Recall ISC observed among Free participants was driven by these choice events, we repeated the analysis using only the 49 non-choice events. The results were largely unchanged: Recall ISC was still significantly above zero in all three conditions (*Romance:* Free: mean *r *= 0.157, 95% CI [0.133, 0.180], t(152) = 13.21, d = 1.07, *p* < 0.001; Yoked: mean *r* = 0.219, 95% CI [0.210, 0.227], t(1377) = 51.43, d = 1.39, *p* < 0.001; Passive: mean *r* = 0.229, 95% CI [0.221, 0.236], t(1484) = 59.03, d = 1.53, *p* < 0.001; one-sample t-tests against zero), and the Free condition continued to show reduced Recall ISC relative to the Yoked and Passive conditions (Fig. 2C, left; *Romance:* F(2,3013) = 15.45, *p* < 0.001, η^2^ = 0.010; post-hoc tests: Free vs. Passive: t(1636) = −5.68, *p* < 0.001, d = −0.48, 95% CI for mean difference [−0.097, −0.047]; Free vs. Yoked: t(1529) = −4.63, *p* < 0.001, d = −0.40, 95% CI for mean difference [−0.088, −0.036]).

In the Yoked and Passive conditions, multiple participants followed the story-path corresponding to each of the 18 unique Free participant story-paths. To ensure that the above-reported higher inter-participant memory similarity in the Yoked and Passive conditions was not due to participants sharing the same story-path, we conducted non-parametric tests of Recall ISC (Fig. 2B, C). We randomly sampled one Yoked and one Passive participant from each of the 18 story-paths to form a sample of 18 Yoked and 18 Passive participants; thus, no pairs within these samples read the same story-path. This process was repeated 10,000 times to generate distributions of Recall ISC for both the Yoked and Passive conditions. The analysis confirmed that the Free condition’s Recall ISC was significantly lower than that of the Passive (*p* < 0.001; excluding choice events, *p* < 0.001) and the Yoked (*p* < 0.001; excluding choice events, *p* = 0.007) conditions (Fig. 2B, C, right).

#### Individual variability in choices made

The option that was selected (1 or 2) at each of the 15 choice-points in the shared story sections was extracted for each Free and Yoked participant (Passive participants made no choices), composing a vector of choice selections. To assess the choice similarity across participants, we computed the Pearson correlation between each pair of subjects’ choice selection vectors, i.e., “Choice ISC”. Within-group Choice ISC was significantly above zero in both conditions (Free: mean r = 0.208, 95% CI [0.165, 0.251], t(152) = 9.55, *p* < 0.001, d = 0.77; Yoked: mean r = 0.255, 95% CI [0.241, 0.269], t(1377) = 36.19, *p* < 0.001, d = 0.97), showing that certain choice options were intrinsically preferred over others. Comparing across conditions, Free participants had significantly reduced Choice ISC (mean r = 0.208) compared to Yoked participants (mean r = 0.255), indicating that agency (full as opposed to partial) induced greater individual variability in terms of which options were selected (t = −2.11, *p *= 0.035, d = −0.18, 95% CI for mean difference [−0.091, −0.003], two-sample t-test). This increased Choice ISC within the Free condition did not drive their increased Recall ISC. Another permutation test showed that Free participants still had significantly reduced within-group Recall ISC compared to their Yoked counterparts with matching Choice ISC (*p* < 0.001); see Supplement S12. These results suggest that agency was the driver of greater individual variability in both memory and choice behaviors.

#### Divergence from the group

Each Free participant’s *memory divergence* score was calculated as one minus the Pearson correlation between their recall performance vector and the group averaged recall performance vector. In other words, the more different their memory performance vector was from the group average, the higher their memory divergence score. Similarly, we calculated each Free participant’s *choice divergence* score as one minus the Pearson correlation between their choice selection vector and the group averaged choice selection vector. Memory divergence and choice divergence were correlated with each other in the full (N = 100) *Romance* sample (*Romance:* *N* = 18: r(16) = 0.405, 95% CI [−0.076, 0.733], *p* = 0.095; *N* = 100: r(98) = 0.296, 95% CI [0.106, 0.465], *p* = 0.003). In other words, the more idiosyncratic their memory for shared events, the more idiosyncratic their choices.

Overall, these results support the idea that agency magnified individual variability in, i.e., *personalized*, both memory and choice behaviors. This effect was observed while all events were held constant across conditions.

### Event recall was predicted by semantic and causal centrality

Narrative networks were computed for each unique story path following the methods of Lee & Chen^21^. For semantic narrative network analysis, each event was converted into an embedding vector using the Universal Sentence Encoder (USE^35^). *Semantic centrality*, a measure of how strongly interconnected a given event was with other events in the narrative via shared meaning, was calculated for each event by averaging its embedding cosine similarity with all other events in the story path. The effect of semantic centrality (*semantic influence*) on memory was computed as the Pearson correlation between semantic centrality and recall (an event-by-event vector of remembered = 1, forgotten = 0) for each participant. For both stories, semantic centrality significantly predicted recall, i.e., a significant semantic influence on memory was observed, in all three conditions (ps < 0.001, one-sample *t*-tests against zero; Supplement S8, Supplementary Table S1 and Supplementary Fig. S7).

For causal narrative network analysis, independent human raters judged which pairs of events were causally linked in a given story path (*Adventure:* 1 rater per path; *Romance:* average of 3 raters per path; see Methods). *Causal centrality*, a measure of an event’s causal connectedness to other events within a narrative, was calculated for each event by averaging across its causal connections with all other events in the story path. The effect of causal centrality (*causal influence)* on memory was computed as the Pearson correlation between causal centrality and event-by-event recall for each participant. For both stories, causal centrality significantly predicted recall, i.e., a significant causal influence on memory was observed, in all three conditions (ps < 0.001, one-sample *t*-tests against zero; Supplement S8, Supplementary Table S1 and Supplementary Fig. S7).

In sum, both semantic centrality and causal centrality predicted recall of interactive narratives, echoing the findings of earlier studies on the effects of semantic and causal relations on memory for narratives^20,21,49,50^.

### Agency reduced the influence of semantic centrality on recall

**Fig. 3:**
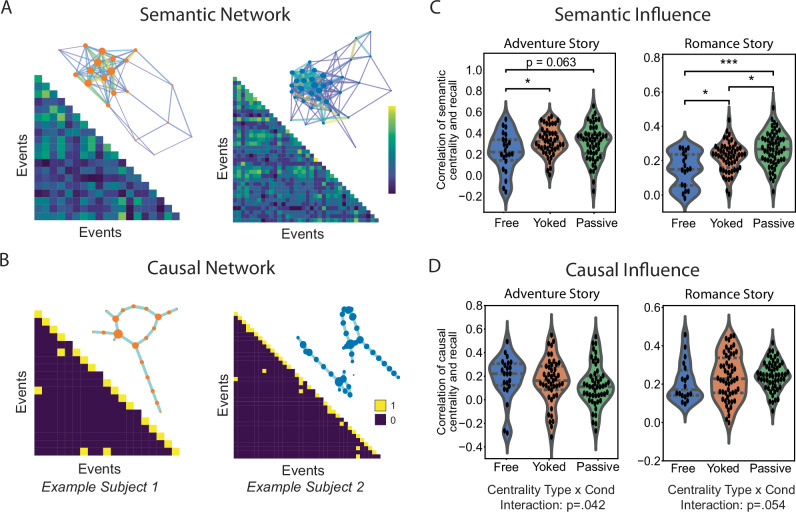
Semantic and causal centrality effects on memory. **A** Semantic similarity matrix showing the USE cosine similarity between pairs of events for example Adventure (left) and Romance (right) stories, and the corresponding semantic network plots for the same stories. Each node in the network graphs (top right corners) represents an event; edges (τ > 0.1) scale in thickness and color by similarity; node size reflects degree centrality. **B** Causal matrix showing the human-judged causal connection (yellow: 1 = causally connected; purple: 0 = not causally connected) between pairs of events for example Adventure (left) and Romance (right) stories, and the corresponding causal network plots for the same stories (network graphs constructed using the same methods as in A). **C** Distributions of semantic influence, i.e., the correlation between semantic centrality and recall performance, in the Adventure story (left) and the Romance story (right) in each condition. Each dot represents one participant. Two-sided t-tests to follow up on the one-way ANOVAs showed that the Free was reduced compared to Yoked (*Adventure*: *p* = 0.015, *Romance*: *p* = 0.003) and Passive (*Adventure*: *p* = 0.063, *Romance*: *p* < 0.001) conditions. **D** Distributions of causal influence, i.e., the correlation between causal centrality and recall performance, in the Adventure story (left) and the Romance story (right) in each condition. Each dot represents one participant.

We next compared the strength of semantic and causal influences on memory across the three conditions (Free, Yoked, Passive). Importantly, because Yoked and Passive participants read the story paths generated by Free participants, event content was matched across conditions; only the degree of perceived agency varied. In the *Romance* story, we observed significant differences across conditions, wherein Free had lower semantic influence on memory compared to Yoked and Passive (*Adventure*: F(2,113) = 3.04, *p* = 0.052, η^2^ = 0.051; post-hoc: Free vs. Yoked: t(65) = −2.50, *p* = 0.015, d = −0.61; Free vs. Passive: t(69) = −1.89, *p* = 0.063, d = −0.45; *Romance*: F(2,123) = 11.46, *p* < 0.001, η^2^ = 0.157; post-hoc: Free vs. Yoked: t(69) = −3.09, *p* = 0.003, d = −0.73; Free vs. Passive: t(71) = −4.43, *p* < 0.001, d = −1.04; Yoked vs. Passive: t(106) = −2.49, *p* = 0.014, d = −0.48; Fig. 3A). In contrast, causal influence on memory did not show a statistically significant difference between conditions (Fig. 3B). There was a significant network type x agency interaction for the *Adventure* story (F(2,113) = 3.27, *p* = 0.042, η^2^ = 0.055) and the same effect that did not reach statistical significance in the *Romance* story (F(2,123) = 2.99, *p* = 0.054, η^2^ = 0.046). Note that semantic and causal centrality were often correlated with each other but had weak or even negative correlations in some story paths, enabling analyses showing that they separately predicted event recall (Supplement S9 and Supplementary Table S2).

These results show that when participants had agentive control over the plot of a narrative, semantic influence (the impact of semantic centrality) on later memory was reduced; meanwhile, causal influence (the impact of the causal centrality) on recall did not show a statistically significant difference under agentive control. This weakening of semantic influence suggests that Free participants’ semantic space may have shifted away from the generic (normative) semantic space captured by generic text embeddings.

### Agency introduces temporal dependencies in memory

**Fig. 4:**
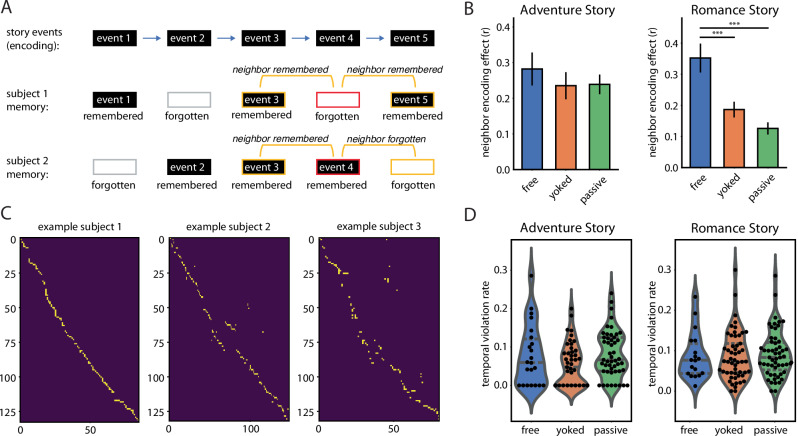
Neighbor encoding effect and temporal violation rate in participants’ recall. **A** Schematic showing how the neighbor encoding effect is calculated. Participant 1 recalls events 1, 3, and 5, producing a recall vector [1 0 1 0 1], while Participant 2 recalls events 2, 3, and 4, producing a recall vector [0 1 1 1 0]. For each event, we compute the average recall score of its neighbors (the previous and the next event), resulting in a “neighbor” recall vector. The neighbor encoding effect is computed as the correlation between the recall vector and the neighbor recall vector; in other words, can the remembered vs. forgotten status of a given event be predicted by the status of its neighboring events? **B** Bar plots comparing the neighbor encoding effect across conditions in the Adventure story (left; Free: *n* = 22, Yoked:* n* = 45, Passive: *n* = 49) and in the Romance story (right; Free: *n* = 18, Yoked: *n* = 53, Passive: *n* = 55). Two-sided t-tests to follow up on the one-way ANOVAs showed that Free was lower than Yoked (p = 0.002) and Passive (*p* < 0.001) conditions in the Romance story. Data are presented as mean values +/− SEM. **C** Three example participants’ recall chronology. Y-axis: Event number during encoding. X-axis: Event number during recall. **D** Temporal violation rate in the Adventure story (left) and in the Romance story (right). Each dot represents the temporal violation rate in one participant’s recall, i.e., the frequency with which each participant recalled events out of order.

We examined whether recall performance for a given event could be predicted by whether its temporally *neighboring* events at encoding were recalled, which we term the “neighbor encoding effect”. First, for each participant and for each event, we calculated the average of the recall scores for the immediately previous and next events at encoding (the neighbors); for the first and last event, there were neighbors on only one side, and thus these entries consisted merely of recall performance for the next and previous event, respectively. This procedure generated a vector of *neighbor recall performance* for each participant. We then calculated the *neighbor encoding effect* as the correlation between the neighbor recall performance vector and the original recall performance vector for each participant (Fig. 4A).

The neighbor encoding effect was positive in all three conditions for both stories (*Adventure* and *Romance*, ps < 0.001), and significantly different across the three conditions in the *Romance* story, with Free having a higher neighbor encoding effect compared to Yoked and Passive (F(2,123) = 12.07, *p* < 0.001, η^2^ = 0.164; post-hoc tests: Free vs. Yoked: t(69) = 3.26, d = 0.77, *p* = 0.002; Free vs. Passive: t(71) = 5.24, d = 1.23, *p* < 0.001; Fig. 4B). Note that despite having the same trend, the *Adventure* story did not show a statistically significant agency enhancement. This is because the Passive participants reached a ceiling for the neighbor encoding effect due to limited and vastly varying story length (*Adventure* vs. *Romance*: 22–59 events vs. 128–135 events). However, additional analysis confirmed that agency can enhance neighbor encoding effect in longer *Adventure* stories, supporting the result for the *Romance* story; see Supplement S13. Overall, these results suggest that agency enhanced the tendency for temporally neighboring events at encoding to share the same subsequent memory status, either both recalled or both forgotten.

The neighbor encoding effect is distinct from the “temporal contiguity effect”, which describes the phenomenon that recalling one item from a randomized list tends to trigger the recall of items which were experienced nearby in time^32,51^; the neighbor encoding effect does not incorporate any information about the temporal order of recall. To examine temporal order effects during recall in our data, we calculated the *temporal violation rate* as the frequency with which each participant recalled events out of order. For each participant, recall was divided into segments (brief sentences). We counted the number of times that a recall segment referred to an event that occurred earlier in the story than the events referred to by the previous recall segment; this was then divided by the participant’s total number of recall segments. There was no statistically significant difference in temporal violation rate across conditions in either story (*Adventure:* F(2,113) = 2.56, *p* = 0.081, η^2^ = 0.043; *Romance:* F(2,123) = 0.10, *p* = 0.908, η^2^ = 0.002). In general, temporal order was remarkably well-preserved, with low temporal violation rates in all conditions (Fig. 4C, D).

### Greater memory divergence was associated with weaker semantic influence and stronger temporal dependence

We next examined how memory divergence scores (Fig. 2) were related to a) the impact of semantic centrality on recall (Fig. 3) and b) the neighbor encoding effect (Fig. 4). Each Free participant’s *semantic influence* score was obtained by calculating the Pearson correlation between semantic centrality and memory performance (same as shown in Fig. 3).

Memory divergence was negatively correlated with semantic influence scores in Free participants. In other words, the more a participant’s recall deviated from other participants in the group, the weaker the effect of semantic centrality on their recall. This was true when including only the 18 Free participants who had yoked counterparts (*Romance:* *N *= 18: r(16) = −0.519, 95% CI [−0.794, −0.069], *p* = 0.027) as well as when using the full sample (*Romance:* *N* = 100: r(98) = −0.460, 95% CI [−0.602, −0.290], *p* < 0.001; Supplement S8 and Supplementary Fig. S8A). Choice divergence, however, was not significantly correlated with semantic influence scores. Note that these comparisons could only be made for the *Romance* story, as the analyses of divergence depend on the shared story sections.

The neighbor encoding effect was also positively associated with memory divergence in Free participants (*Romance:* *N* = 18: r(16) = 0.496, 95% CI [0.038, 0.782], *p* = 0.036; *N* = 100: r(98) = 0.304, 95% CI [0.114, 0.472], *p* = 0.002; Supplementary Fig. S8B). In other words, the more a participant’s recall deviated from other participants in the group, the more that participant’s neighboring events tended to have the same recall status (remembered or forgotten). The neighbor encoding effect was negatively correlated with semantic influence scores in Free participants, across both stories, while only the full (*N* = 100) *Romance* sample reached statistical significance (*Adventure:* *N* = 22: r(20) = −0.348, 95% CI [−0.671, 0.087], *p* = 0.113. *Romance:* *N* = 18: r(16) = −0.324, 95% CI [−0.687, 0.168], *p* = 0.189; *N* = 100: r(98) = −0.347, 95% CI [−0.509, −0.162], *p* < 0.001; Supplementary Fig. S8C). However, when including both semantic influence and the neighbor encoding scores in a multiple linear regression predicting memory divergence, semantic influence score was a significant predictor (β = −0.42, *p* < 0.001) while the neighbor encoding effect was not statistically significant (β = 0.09, *p* = 0.087); model R^2^ = 0.235.

Overall, the degree to which a participant’s memory was idiosyncratic in terms of which events they recalled (*memory divergence*) was negatively correlated with impact of the story’s semantic network on memory – in line with the idea that agency personalizes memory.

### Consequences of having your choices denied

In the results reported above, Yoked subjects generally exhibited similar memory performance to the Passive subjects—similar idiosyncrasy in event recall, in semantic and causal centrality effect on memory, and in neighbor encoding effects, with their group mean falling in between that of the Free and Passive condition. Given that Yoked subjects had a varied number of choices granted/denied (see Supplementary Fig. S4), these results aligned with the design of ‘partial agency’ for the Yoked condition.

Nonetheless, having one’s agency denied can have unique memory effects at local choice events: memory for denied choice events was selectively reduced compared to their choice-granted counterparts in the Free condition (*Adventure:* t(88) = −2.49, *p* = 0.015, d = −0.53, 95% CI for mean difference [−0.253, −0.029]; *Romance:* t(104) = −2.44, *p* = 0.017, d = −0.47, 95% CI for mean difference [−0.186, −0.019], two-sample t-test); individual differences contributed to variation in the tendency to selectively recall or forget the denied choice events (see Supplement S6 for details).

The percentage of choices granted in the Yoked participants was not predictive of individual’s recall performance, recall similarity to their Free and Passive condition counterparts, semantic centrality effects on memory, nor their neighbor encoding effects (all ps > 0.3; see Supplement S7 for details); however, higher percentage of choices granted predicted greater individual tendency to forget the choice-denied events in the *Adventure* story (*Adventure*: r(42) = 0.335, 95% CI [0.042, 0.575], *p* = 0.026. *Romance*: r(49) = 0.137, 95% CI [−0.144, 0.398], *p* = 0.336; see Supplement S6 and S7 for details).

Together, these results suggest that in a context lacking full agentive control, perceived agency and their effects on memory could vary across individuals in non-systematic ways. The one exception is that with more control in such agency-uncertain contexts, the more one has reduced recall for the agency-denied events.

## Discussion

In this study, we examined whether agency resulted in more personalized memories. Participants read one of two interactive choose-your-own-adventure narratives followed by free recall. We compared memory behavior across three conditions: Free, in which choices were always granted (full agency); Yoked, in which choices were sometimes granted (partial agency); and Passive, in which participants did not get to choose (no agency). Agency shaped episodic memory in at least three ways: (i) agency personalized memory such that participants in the Free condition were less likely to recall the same shared events as one another, despite all conditions being exposed to identical story content; (ii) agency weakened the capacity for generic semantic relations to predict memory while leaving the influence of causal relations unaffected; and (iii) agency introduced dependencies between temporally adjacent events such that they were more likely to share the same mnemonic fate (i.e., both remembered or both forgotten). Overall, we propose that agentive control changes how basic determinants of episodic memory operate during naturalistic experience, potentially as a consequence of activating self-specific knowledge and past experiences in the service of making decisions.

Agency is known to benefit learning and memory^7,10–12,14–16^. Having the ability to choose – or sense that one could choose – results in better memory. This boost has been attributed to choice driving reward signals^38–40^ and encouraging more elaborative self-related encoding^11^. These accounts suggest that if people encounter a sequence of events, with some requiring a choice, choice events will be better remembered than non-choice events. Further downstream, this would result in individuals with agency showing more across-person similarity in terms of which events they recall, relative to individuals without agency – agency would *standardize* memories. In our study, each participant in the Free condition made choices to create their own unique narrative; Yoked and Passive participants then read these narratives. Crucially, the *Romance* story was designed such that 50% of events, distributed across the story, were read by all participants regardless of the agency condition they were assigned to or the choices they made. This feature allowed us to examine individual variability in terms of which events were recalled, with event content held constant across participants and levels of agency. As opposed to standardizing, we found that agency *personalized* memory for shared events: agency magnified individual variability, both for which events were later remembered and for which options were chosen. That is, participants in the Free condition tended to recall different events and make different choices from each other, while participants within the Yoked and Passive conditions were more similar to each other.

What factors drive the personalization in memory and choice observed in the Free condition? We propose that the need to make decisions about how the story will unfold causes participants to activate self-specific information^9^. For example, imagine the story entailed a choice about where the protagonist was to eat dinner (e.g., Italian vs. Lebanese restaurant). If the participant happened to have spent the summer in Italy, that person’s choice and subsequent interpretation of the Italian restaurant events – a busy patio, surprisingly excellent wine, a distracted waiter – could mingle with features from their own personal experiences visiting Italy. As a result, the features of the story that stand out to the participant may become more idiosyncratic. Not all decisions, however, require such self-specific knowledge; for example, in bandit-style tasks where participants make choices that maximize their rewards, only learned information about the value of stimuli is needed. In our study, the use of narratives – complex events that resemble real-life situations – was likely important for eliciting the personalizing effect of agency. This has broad implications for how human memory operates in daily life, as the amount of control that a person has over their environment varies greatly in natural settings (e.g., being a driver vs. a passenger in a car; creating a meal vs. following a recipe; choosing which topics to study vs. following a predetermined lesson plan). Our findings suggest that the effects of agentive control on memory may be better understood as changing precisely what is remembered across individuals, as opposed to a general enhancement of learning.

Agency reduced semantic influence on memory, while leaving causal influence unaffected. Specifically, semantic centrality (i.e., the degree to which an event has strong or numerous semantic similarities to other events in the narrative) predicted which events would be recalled in all conditions, but the prediction was weakest when participants were able to control the story (Free condition). We propose that this result reflects a change in how individuals organize concepts – a warping of their semantic space – when they have agency over current events. Vector representations of events semantics were calculated using sentence embeddings^35^, and these were necessarily generic; such neural network language models have no ability to capture idiosyncrasies of an individual’s own interpretation of a text. If having agency activated self-specific knowledge, one consequence could be that individuals’ interpretations of, or associated thoughts with, each story event may have diverged from the generic semantic representation. Consider our previous example about the Italian restaurant. This participant’s mental representation of the word “spaghetti” – perhaps associated with a delicious meal from their trip as well as a feeling of embarrassment from the moment they realized they had lost their wallet after attempting to pay the bill – may diverge from the normative associations learned by a language model. For this reason, we suggest that our finding that agency enhances the idiosyncrasy of, or personalizes, memory is meaningfully related to the observation that agency reduced semantic narrative network effects on recall. Indeed, in an individual differences analysis, the degree to which a person’s memory was idiosyncratic (i.e., *memory divergence score*) was negatively related to semantic influence on memory for that person (r = −0.519). Future studies may further test this idea by probing how agency reshapes how we organize concepts; for example, by directly questioning individuals about perceived semantic similarity between events or topics related to a laboratory experience presented with and without agency.

Why was causal influence on memory unaffected by agency? That is, causal centrality (the degree to which an event has strong or numerous causal connections to other events in the narrative) predicted which events would be recalled in all conditions, and the strength of this prediction was *not* modulated by whether participants were able to control the story. This may seem surprising, as making choices is conceptually linked to causality^52–55^. One might have reasonably expected that because Free condition participants were able to effectively “cause” future events via their choices, these cause-effect links might be added to the causal narrative network and interfere with the ability of “standard” causal centrality to predict memory. Or, perhaps Free condition participants would have a heightened awareness of causal connections in the narrative, which could change the way that these causal connections shaped memory. Nonetheless, our results are not compatible with these views. Instead, our findings point to a potential separation between 1) the causality inherent in making decisions and 2) the causal relations between events that are extracted in the course of narrative comprehension. The distinction between the two, and their possible interplay, is an interesting topic for future research^24^. A parsimonious interpretation of the current data may be that causal connections in a narrative play an essential role in comprehension and memory, even in the absence of choose-your-own-adventure-style agency^19–21,56–59^. The influence of causal associations on memory for narratives, then, may be relatively impervious to the kinds of manipulations of agency used in this study.

How did agency alter the role of temporal order in memory? This question should be split into two parts: the effects of agency on temporal relations at encoding and at recall. First, did agency change the way that temporal relations between events *at encoding* influenced whether an event would be later remembered? Indeed, we found evidence that agency introduced temporal dependencies in memory. Specifically, agency enhanced the tendency for events which were adjacent during encoding to share the same mnemonic fate, either both recalled, or both forgotten. This “neighbor encoding effect” was positive in all three conditions for both stories, and highest for the Free condition in one story. Our finding adds to recent work showing that agency can change memory for the temporal properties of events at encoding. For example, Houser et al.^18^ report an association between participants being able to choose the order of studied events in a text-based room exploration game and more accurate temporal order memory for the game experience. Such temporal dependency could arise from idiosyncratic binding of neighboring events, as agency may promote activation of self-relevant knowledge that links adjacent moments in the narrative. Not incompatible with this, another interpretation is that participants with agency may have formed expectations about upcoming events^10,53^, strengthening associations between temporally adjacent events^14^. However, this latter account might predict stronger effects specifically at choice points. While agency’s enhancement of neighbor encoding effect is indeed prominent around choice events, we saw this across all events, even when choice events were excluded. This suggests a more general influence of agency on how adjacent events are bound in memory at encoding.

Second, did agency change temporal properties of events *at recall*? Free recall studies using sequences of unrelated memoranda have established the “temporal contiguity effect”: items are likely to be recalled in a similar order to that in which they were presented^60^. In this study, we calculated the *temporal violation rate* as the frequency with which each participant recalled events out of order. No statistically significant differences between agency conditions were observed. However, it is important to note that most narratives are linear chains of causally connected events, thus confounding temporal and causal relations. Under these conditions (and true in our study), interpretation of narrated recall through the lens of temporal contiguity is problematic; temporal contiguity is typically high, but it may be driven by a combination of causal and temporal structure^61^. Antony et al.^23^ solved this problem by employing a narrative in which temporal order, story chronology, and causal linkage were separable. They found that causal relations were the dominant factor in determining the order of recalled events. These observations suggest that, while both random-item lists and narratives tend to be recalled in the order of original presentation, temporal order drives the former while causal relations drive the latter phenomenon. In sum, our study revealed evidence that agency changed the way that temporal relations between events at encoding influenced later memory (i.e., agency increased neighbor encoding effect), but no evidence that temporal order during recall is affected by agency (i.e., agency did not affect temporal violation rate).

A necessary aspect of research using naturalistic stimuli such as narratives is that labeling of many stimulus and behavioral response features relies on human judgments: e.g., in the current study, delineation of event boundaries, causal links between events, segmentation of participants’ recall and assignment of those segments to the story events. For each such variable, researchers must choose between 1) having the features “curated” by the researchers themselves or by trained labelers, 2) estimating population-level human judgments by running a separate experiment (commonly performed for event boundary selection), or 3) some combination. The “curation” approach avoids some of the messiness which comes with human data, while the “separate experiment” approach may allow for scientific claims arising from the human judgment data. In our study, we largely took the curation approach (see Methods). Interestingly, recent publications have demonstrated that LLMs can perform some of these judgment tasks with comparable-to-human performance (e.g., recall-to-story content matching^62,63^ and event segmentation^64,65^). As the field moves toward adopting such automated annotation pipelines, the high-quality human-rated data from the current study will serve as a valuable resource for training and validating these future systems. However, it is also important to note that the judgments of LLMs may not be more “objective” than those of humans; certain judgments have no correct answer, but rather should be expected to generate a distribution of responses from humans. It would be a mistake to replace human ratings with automated tools if doing so creates an illusion that the cognition underlying those judgments is well-understood^66^.

In summary, we studied the impact of agency on memory for naturalistic experiences, rich in temporal, semantic, and causal relations between events. Agency enhanced the distinctiveness of memories, driving individuals apart in terms of which events they recalled. This personalizing effect on memory was related to a decreased emphasis on semantic relations between story events in predicting subsequent recall, which we interpret as a warping of individuals’ conceptual space towards their personal experience and away from normative semantic relations. At the same time, agency did not modulate the ability of causal relations between events in predicting recall, and agency increased the likelihood of neighboring events during encoding sharing the same mnemonic status at later recall. Our findings highlight agency as an important determinant of memory for naturalistic events, best understood as changing precisely what is remembered across individuals, as opposed to enhancing learning overall.

## Methods

### Participants

324 participants (*Adventure* story: 22 Free, 45 Yoked, 49 Passive; *Romance* story: 100 Free, 53 Yoked, 55 Passive) completed the main experiment (183 females, 139 males, 2 non-binary) using the Prolific website, with an average age of 28.0 ± 8.8 yr (mean ± s.d.). Another 52 participants (28 for the 3 selected *Adventure* story-paths; 24 for the 3 selected *Romance* story-paths) completed the causal rating task on the 6 story-paths selected to test GPT-4 (21 females, 31 males) using the Prolific website, with an average age of 27.3 ± 7.4 yr (mean ± s.d.). All participants were proficient in English. Informed consent was obtained in accordance with procedures approved by the Johns Hopkins University Institutional Review Board.

### Stimuli

The *Adventure* story was a slightly modified version of an interactive story titled “Before Alice: Xenization”, authored by Moola Hoola (Fig. 1A). The story takes place in a universe inspired by Lewis Carroll’s *Alice’s Adventures in Wonderland*, in which the protagonist, Alice, wakes up in a magical world and over the course of the story discovers that she is the Princess of Hearts. We chose the Adventure story because it is an engaging genre that most participants can relate to and enjoy. There were 8-24 choice events, depending on the choices made by each participant; each choice event had 2-4 options. Participants generated 22 unique *Adventure* story-paths via their choices. These story-paths varied in length (400-1000 words) and number of events (22-59 events). The *Romance* story was titled “The Monthiversary” and was written by Ria Gualano and refined for experiment purposes via discussion among the study authors. This genre was selected to provide a contrasting narrative style, promoting generalizability of the results across different types of stories. The story follows the actions of a woman recovering from a recently ended relationship and potentially finding new romance. It was designed such that half of its events were identical (“shared sections”) across all story-paths, while the other half were split into 3 possible storylines (Fig. 1B). There were 7 stretches of shared sections distributed across the story. The number of choice events was predetermined at 29 per story-path, 15 of which were in the shared sections, regardless of what choices the participants made. Each choice event had 2 options. Participants generated 100 unique *Romance* story-paths via their choices. These story-paths had varied text length (4800-5200 words) and number of events (128-135 events). All revisions to the story were made prior to the start of data collection.

### Experimental procedures

In the main experiment, participants read the story one sentence at a time in a self-paced manner, pressing the ‘Enter’ key to proceed to the next sentence. Periodically they encountered a choice event, and available options were presented below. In the *Adventure* story, 2 to 4 options were offered; *Free* participants used their mouse to drag the options on the screen to rank their preferences from most to least preferred (top to bottom), then clicked a ‘Next’ button to continue. *Yoked* participants experienced the same procedures, except that their story-path was pre-assigned to follow one of the 22 *Free* story-paths; consequently, they had some of their choices granted, while others were denied, depending on whether their choice coincided with the pre-assigned one. *Passive* participants experienced the same procedures but saw only one option at each choice event; their story-path was also pre-assigned to follow one of the 22 Free story-paths. Each *Free* story-path was followed by 2-3 *Yoked* and 2-3 *Passive* participants. In the *Romance* story, *Free* participants generated 100 story-paths based on their choices, and 18 *Free* participants’ story-paths were selected for *Yoked* and *Passive* participants to follow (see ‘Behavioral data preparation’ for details). The procedures for the Romance story were identical to those for the Adventure story except for the following: 1) each choice event offered only 2 options; 2) *Free* and *Yoked* participants clicked on their preferred option, rather than dragging; 3) *Passive* participants saw both options (rather than only one) with an arrow pointing to the pre-assigned option, on which they were required to click in order to continue. Each *Free* story-path was followed by 2-3 *Yoked* and 2-3 *Passive* participants. See *Data Availability* for demonstrations of the experiment^67^.

Before the experiment, participants completed a short practice story to familiarize them with the interactive paradigm. After the experiment, participants completed several trait surveys before continuing to the free recall task. They were then instructed to type their recall of the story in as much detail as possible. The instructions emphasized the importance of recall completeness over accurate sequencing of events. Participants typed in a one-line window in which they could see approximately the last 10 words of their recall. Participants were not able to click or scroll through their recall, and they could not backspace past a period, meaning they could only edit the current sentence they were typing. We have implemented an identical procedure in previous work^21^, with the goal of minimizing participants’ ability to reorganize, polish or otherwise edit their memories after typing them. This strategy forces typed recall to better approximate spoken recall, where participants cannot change what they have already said aloud. This procedure is consistent with standard free-recall paradigms, in which participants study lists and are later asked to recall as many studied items as possible “in any order,” and robust temporal contiguity effects reliably emerge in the resulting output sequences^32,60,68,69^. For the *Romance* story only, a multiple-choice recognition test was administered after free recall. The *Romance* story experiment required more time to complete (1.5–2.5 h) compared to the *Adventure* story (20–50 mins), due to the longer narrative and the additional recognition memory test. To ensure data quality, incentives were offered for high-quality recall and recognition responses for the *Romance* story. Beyond the base payment, participants were informed (veridically) that an additional bonus would be awarded to those scoring in the top 40% for recall, and another bonus for top performance in the recognition test ($2 for the top 40% recall, and $2 for the top 40% recognition).

### Behavioral data collection and preparation

The *Romance* story was read by 100 participants in the *Free* condition, resulting in 100 unique story-paths, of which 18 were chosen based on maximizing story paths difference from each other to ensure a good variation of stories adopted. Narrative diversity was calculated as follows. The story was structured so that half of its content was shared across all story-paths, while the remainder branched into three distinct storylines, culminating in two possible endings. Participants in the same storyline encountered similar overall narratives, though their choices caused minor divergences in many places. The similarity between two *Free* participants’ story-paths increased with the similarity of their choices. We calculated the similarity of choices between each pair of *Free* participants within the same storyline, then selected the three story-paths with the lowest similarity in choices for each story version, resulting in the 18 selected story-paths (3 story-paths × 3 storylines × 2 endings) which would be followed by the *Yoked* and *Passive* conditions. When appropriate, statistical comparisons across conditions utilized these 18 selected *Free* participants’ story-paths, which ensured that story content was held constant. The shared half of the *Romance* story enabled us to examine the effects of agency on within-group variability in memory and choices. To ensure that the 18 selected Free participants were representative of the full sample of 100 Free participants in terms of memory and choice variability, we compared their mean variability against the distributions of the full group. Results indicated no statistically significant differences between the selected subset and the full Free condition sample; see Supplement S11.

#### Human ratings for naturalistic stimuli

Our laboratory maintains a team of raters (research assistants) who routinely perform tasks pertaining to processing and ratings of naturalistic stimuli, as we have similar needs across many of our experiments (speech transcription, stimulus labeling, matching, etc.). The raters are trained by a graduate-student team leader who also regularly reviews the raters’ work for quality and provides feedback. They followed a standardized written instruction set (publicly available in the “instruct-raters” folder on GitHub; see Data Availability^67^), ensuring consistency across coders.

#### Event segmentation

In order to calculate recall performance, each story needed to be segmented into discrete events. A team of experienced raters identified event boundaries by marking transitions in scenes, topics, locations, times, actions, emotions, or other significant shifts, discussing markings with each other until consensus was reached (see *Data Availability* for instructions^67^). Choice options were treated as separate events due to their distinct presentation (different font, color, background, and interaction mode) and their natural demarcation from the narrative flow. Following event segmentation, the *Adventure* story contained 169 events in total, with the 22 *Free* participants experiencing between 22 and 59 events. The *Romance* story contained 342 events in total, with the 100 *Free* participants experiencing between 128 and 135 events.

#### Matching recall to story-paths

Post-experiment, participants’ written recall was corrected for spelling and grammatical errors with minimal changes by experienced in-lab staff. Recalls were then segmented based on both punctuation and topic shifts, corresponding to sentences or parts of sentences. Each recall segment was assigned to the events in the unique story-path it referred to, with segments potentially aligning with none, one, or multiple events. Hence, the recall score for each event was determined as 0 (not recalled) or 1 (recalled).

#### Causality ratings for the main experiment

For the *Adventure* story, experienced raters analyzed the causal relationships between events for each of the 22 story-paths. The raters read through a story-path (one rater per story-path) and identified pairs of events with a strong causal relationship, where one event directly caused another. They were cautioned against listing event pairs based merely on their chronological order (e.g., “you broke your leg” not because “you woke up in the morning”, but because “someone pushed you down the stairs”), and encouraged to keep criteria consistent throughout their ratings. The *Romance* story, with its 100 story-paths, was rated in the same manner by an average of 3 raters (range: 1–6) per path. The finished causal ratings were lists of event pairs, from which we constructed an event x event causal matrix for each story-path, representing its causal network. These causal matrices denoted event pairs with causal connections as 1, and all others as 0. When multiple raters rated a story-path, the average matrix was computed. See Supplement S1 and Supplementary Fig. S1 for information about a GPT-based method that we developed for identifying causal relations in narratives. The ChatGPT-based causal-rating agent is publicly available (https://chatgpt.com/g/g-dCYo6ta2J-causal-rater-for-story) with corresponding human and AI causal-rating data included in the repository.

### Statistical tests and reports

Comparisons across conditions included all three conditions (Free, Yoked, Passive) unless otherwise noted. Group mean differences were tested using one-way ANOVAs, with follow-up t-tests conducted only when the overall effect was significant. For analyses involving multiple factors (e.g., semantic vs. causal network effects across conditions), repeated-measures ANOVAs were used, again followed by targeted t-tests when warranted by significant main or interaction effects. Only follow-up comparisons that were significant, consistent across measures, and replicated across both stories are reported in the Results. All *t*-tests were two-sided. All figures display error bars representing the standard error of the group mean. Significant between-group differences are marked on figures using asterisks, with * for *p* < 0.05, ** for *p* < 0.01, and *** for *p* < 0.001, and trends (*p* < 0.08) indicated numerically. Full analysis code and test statistic, including all parametric test statistic (e.g. F, t, r) with confidence intervals, effect sizes, degrees of freedom, *p*-value, and non-parametric tests are provided on GitHub (see Code Availability^67^).

#### Averaging correlations

In all instances where we performed statistical tests involving Pearson correlation, we additionally conducted the same tests but with a Fisher z-transformation to stabilize the variance across the distribution of correlation values. These additional analyses confirmed the results reported in the current manuscript. Full analysis code and statistical outputs, including the Fisher r-to-z transformed Z-test results on the correlation values are provided on GitHub (see Code Availability^67^).

### Inter-participant memory similarity and choice similarity

The *Romance* story, by design, had half of its events shared across all participants (“shared story sections”), regardless of condition (Fig. 1A). While participants made many choices during these shared story sections, unbeknownst to them, all choice options led to the same outcomes. This allowed us to examine inter-participant variability in terms of memory (which events were recalled) and choice behavior (which options were selected) when all events were perfectly matched across participants, i.e., all participants read these events, and the events were composed of identical text.

#### Variability in memory for events

A recall score (0 = forgotten, 1 = recalled; see Methods for details) for each of the 64 events in the shared story sections was extracted for each participant, composing a vector of recall performance (Fig. 2A). *Inter-participant memory similarity* was computed as the Pearson correlation between each pair of participants’ recall performance vectors (Fig. 2A, B). While Phi coefficient or Chi-squared tests are often used for assessing relationships between binary variables, here we chose to use Pearson correlation because for two binary vectors, the Pearson correlation coefficient is mathematically identical to the Phi coefficient, and Chi-squared tests independence at the group level rather than measuring pairwise similarity of recall patterns.

#### Variability in choices made

The option that was selected (1 or 2) at each of the 15 choice-points in the shared story sections was extracted for each Free and Yoked participant (Passive participants made no choices), composing a vector of choice selections. *Inter-participant choice similarity* was computed as the Pearson correlation between each pair of participants’ choice selection vectors.

#### Memory divergence score

Each Free participant’s *memory divergence* score was calculated as one minus the Pearson correlation between their recall performance vector and the group averaged recall performance vector, i.e., the more different their memory performance vector was from the group average, the higher their memory divergence score. Similarly, we calculated each Free participant’s *choice divergence* score as one minus the Pearson correlation between their choice selection vector and the group averaged choice selection vector.

### Semantic and causal centrality calculations

Following the methods of Lee & Chen, for semantic narrative network analysis, each event was converted into an embedding vector using the Universal Sentence Encoder (USE^35^). *Semantic centrality*, a measure of how strongly interconnected a given event was with other events in the narrative via shared meaning, was calculated for each event by averaging its embedding cosine similarity with all other events in the story path. The effect of semantic centrality on memory was computed as the Pearson correlation between semantic centrality and recall (an event-by-event vector of remembered = 1, forgotten = 0) for each participant.

For causal narrative network analysis, independent human raters judged which pairs of events were causally linked in a given story path (*Adventure:* 1 rater per path; *Romance:* average of 3 raters per path). *Causal centrality*, a measure of an event’s causal connectedness to other events within a narrative, was calculated for each event by averaging across its causal connections with all other events in the story path. The effect of causal centrality on memory was computed as the Pearson correlation between causal centrality and event-by-event recall for each participant.

Network visualization (Fig. 3A, B) in matrix format showed the event-by-event pairwise Pearson similarity; those in the graph format (top right corners) are constructed from the similarity matrices for visualization purposes only: each node corresponds to a story event; edges connect pairs of events if their similarity exceeds a fixed threshold of τ = 0.1, with the edge thickness and color intensity proportional to the similarity weight; node size is scaled by weighted degree centrality (sum of connected edge weights); node positions are determined by a force-directed layout (Fruchterman–Reingold in MATLAB), which places more strongly connected events closer together. Note that the absolute 2D node coordinates, axes, and overall orientation carry no additional meaning and can rotate/flip without changing the underlying relationships. Node color is used solely as a visualization aid to distinguish example subject 1 (orange) from example subject 2 (blue).

### Neighbor encoding effect

We examined whether recall performance for a given event could be predicted by whether its *neighboring* events at encoding were recalled, which we term the “neighbor encoding effect”. First, for each participant and for each event, we calculated the average of the recall scores for the immediately previous and next events at encoding (the neighbors); for the first and last event, there were neighbors on only one side, and thus these entries consisted merely of recall performance for the next and previous event, respectively. This procedure generated a vector of *neighbor recall performance* for each participant. We then calculated the *neighbor encoding effect* as the correlation between the neighbor recall performance vector and the original recall performance vector for each participant.

The neighbor encoding effect is distinct from the concept of temporal order memory, which refers to the tendency for recall to preserve the original order of events in time. Temporal order memory is commonly characterized by the sequential retrieval of items in the order they were experienced. In contrast, the neighbor encoding effect does not incorporate any information about the temporal order in which events are recalled. Instead, it focuses solely on how the encoding of events adjacent to a target event contributes to the recall performance of that event, regardless of their order during retrieval. In fact, temporal order memory was not predictive of one’s neighbor encoding effect across all participants in either story.

In the current study, temporal order memory was measured using the temporal violation rate. For each participant, recall was divided into segments, and we counted the number of times a segment referred to an event that occurred earlier in the story than events in the preceding segment. This count was then normalized by dividing by the total number of recall segments for each participant. Unlike the common temporal order memory measure of lag-conditional response probability (lag-CRP), the temporal violation rate better suits our human recall data. Lag-CRP measures the likelihood of recalling items based on their temporal proximity to the previous recall and assumes a strictly sequential recall process, but in our study, each recall segment can refer to multiple events that may or may not be sequential. This disrupts the assumptions of lag-CRP, making it unsuitable for our analysis. The temporal violation rate, by contrast, captures temporal order memory more effectively by accounting for the flexibility inherent in human recall.

Because the neighbor encoding effect is computed using correlations, a potential concern is that it might be biased by individual differences in their overall memory performance. To rule out this possibility, we checked that the overall recall performance was statistically equivalent across the Free, Yoked, and Passive conditions in both stories (see Supplementary Fig. S2).

#### Citation diversity statement

Recent work in several fields of science has identified a bias in citation practices such that papers from women and other minority scholars are under-cited relative to the number of such papers in the field^70,71^. Here we sought to proactively consider choosing references that reflect the diversity of the field in thought, form of contribution, gender, race, ethnicity, and other factors. First, we obtained the predicted gender of the first and last author of each reference by using databases that store the probability of a first name being carried by a woman^72^. By this measure and excluding self-citations to the first and last authors of our current paper, our references contain 13.33% woman(first)/woman(last), 13.33% man/woman, 5.63% woman/man, and 67.70% man/man. This method is limited in that a) names, pronouns, and social media profiles used to construct the databases may not, in every case, be indicative of gender identity and b) it cannot account for intersex, non-binary, or transgender people. Second, we obtained predicted racial/ethnic category of the first and last author of each reference by databases that store the probability of a first and last name being carried by an author of color^73,74^. By this measure (and excluding self-citations), our references contain 2.80% author of color (first)/author of color(last), 13.70% white author/author of color, 12.96% author of color/white author, and 70.55% white author/white author. This method is limited in that a) names and Florida Voter Data to make the predictions may not be indicative of racial/ethnic identity, and b) it cannot account for Indigenous and mixed-race authors, or those who may face differential biases due to the ambiguous racialization or ethnicization of their names. We look forward to future work that could help us to better understand how to support equitable practices in science.

### Reporting summary

Further information on research design is available in the Nature Portfolio Reporting Summary linked to this article.

## Supplementary information

Supplementary information

Reporting Summary

Transparent Peer Review file

## Data Availability

The raw and fully rated behavioral data generated in this study (including recall ratings, event segmentations, and causal ratings), complete story stimuli, all rater-instruction materials, together with demonstrations of the choose-your-own-adventure paradigm, are available at GitHub (https://github.com/xianNeuro/agency-personalizes-episodic-memory.git), linked to Zenodo (https://zenodo.org/records/19124153). The human and AI causal-rating data used to evaluate the performance of the AI causal-rating agent alongside the prompt used are provided in the repository (under *supplement*).

## Code availability

All code used to generate the analyses and figures in this manuscript is available at the GitHub repository (https://github.com/xianNeuro/agency-personalizes-episodic-memory)^67^. The analyses and figures in the current manuscript used code available through Python and MATLAB. Custom scripts that can be used to reproduce the figures from the source data are included in the GitHub repository. The repository contains the full statistical analysis pipeline (*stats_code*), complete test outputs (including parallel analyses using Fisher Z-transformed correlation values), and all figure-generation scripts (*figures*), accompanied by detailed instructions to facilitate full reproducibility.
