# Supplementary material for: Interactive narratives reveal the personalizing effect of agency on episodic memory
Source: Nat Commun. 2026 Jun 6;17:7272. doi: 10.1038/s41467-026-73907-2 (PMC13402355; doi:10.1038/s41467-026-73907-2)
Supplement: Supplementary file 1 — Supplementary information [file 41467_2026_73907_MOESM1_ESM.pdf]

Supplementary Note 1. GPT-4 identifies causally related events in narratives on par with human raters.

Recent advances in large language models have enabled automatic narrative segmentation [1] and labeling of narrative recall [2] with performance comparable to humans. Thus, we examined whether GPT-4 could also identify pairs of causally related events in narratives. Three *Adventure* story-paths (number of words: 1047-1320; number of events: 40-60) and three *Romance* story-paths (number of words: 4818-5160; number of events: 129-131) with a variety of causal structures were selected from among the story-paths generated by *Free* participants. For each of these we collected causal ratings from a new set of 7-10 participants.

The new group of participants was recruited from Prolific. Three *Adventure* story-paths were selected because they contained a distinctive long-range causal event pair (implanted by the experimenters at the outset of the experiment), where an early event in the story directly caused an event much later. The long-distance causal relation was consistently identified by most human raters and allowed quick evaluation of GPT-4's outputs. The three *Romance* story-paths were randomly chosen, one from each storyline in the pool of 18 story-paths used for yoking. The causal assessments from Prolific participants were deemed valid provided they adhered to the following standards: 1) The rationale behind the cause-and-effect relationships was logical and in line with the instructions; 2) a sufficient number of cause-and-effect pairs were identified to encompass the story's full scope; 3) the criteria for causal rating remained consistent throughout the evaluation process. Each of the six chosen story-paths received valid causal ratings from 7 to 10 participants.

We developed a GPT-4 Agent (https://chatgpt.com/g/g-dCYo6ta2J-causal-rater-for-story) using prompt modified from the causal rating instructions for human participants (https://github.com/xianNeuro/cyoa/tree/main/instruct-raters). In order to judge the quality of GPT's causal ratings, we examined two sets of metrics. First, we tested how similar humans were to each other, how similar GPT was to itself across queries, and how similar individual humans were to individual GPT queries (five iterations); in each case, we calculated the Pearson correlation between causal centrality vectors. For all six stories, these correlations were significantly higher than zero (all $ps < 0.03$), showing that GPT was substantially similar to humans in the causal ratings it produced (Supplementary Figure S1A). Second, we compared the magnitudes of 1) correlations of individual humans with each other, relative to 2) correlations of individual human raters with individual trials of GPT. We found no difference for the three *Adventure* story-paths ($ps > 0.2$), but higher similarity among humans than between humans and GPT for the *Romance* story-paths ($ps < 0.04$; Figure S1A). That is, GPT-4 was indistinguishable from humans at generating causal ratings for the *Adventure* story but was detectably different from humans for the *Romance* story.

To assess generalization to other narrative formats, we used the same prompt with six movie narratives (annotations of movies, similar to a screenplay), ranging from 569-2105 words in length. Causally related events for the movie narratives were identified by 13 human raters. GPT was queried 10 times per movie narrative. Again, causal centrality was rated in a consistent manner across humans and across trials of GPT, and the two were significantly similar to each other ($ps < 0.001$; Supplementary Figure S1B). Comparing the magnitudes of correlations, we found that for three movies GPT was

indistinguishable from humans (ps > 0.05), and for three movies GPT was detectably different from humans (ps < 0.001).

Positive correlations between human and GPT performance in terms of causal centrality did not necessitate that GPT's causal ratings would predict human memory. Thus, we compared the causal centrality vectors produced by GPT to human recall vectors. We found that GPT and human-generated causal centrality vectors predicted human recall performance similarly well (*Adventure*: mean r for humans = 0.32, for GPT = 0.36; $t(28) = -.821$, $p = .419$; *Romance*: mean r for humans = 0.27, for GPT = 0.23; $t(40) = 1.36$, $p = .182$; Supplementary Figure S1C).

In summary, we successfully developed a prompt for GPT-4 that enabled the identification of causally related events in both text-based narratives and movie annotations. GPT's judgments broadly resembled that of humans, though in some cases GPT was detectably different from humans.

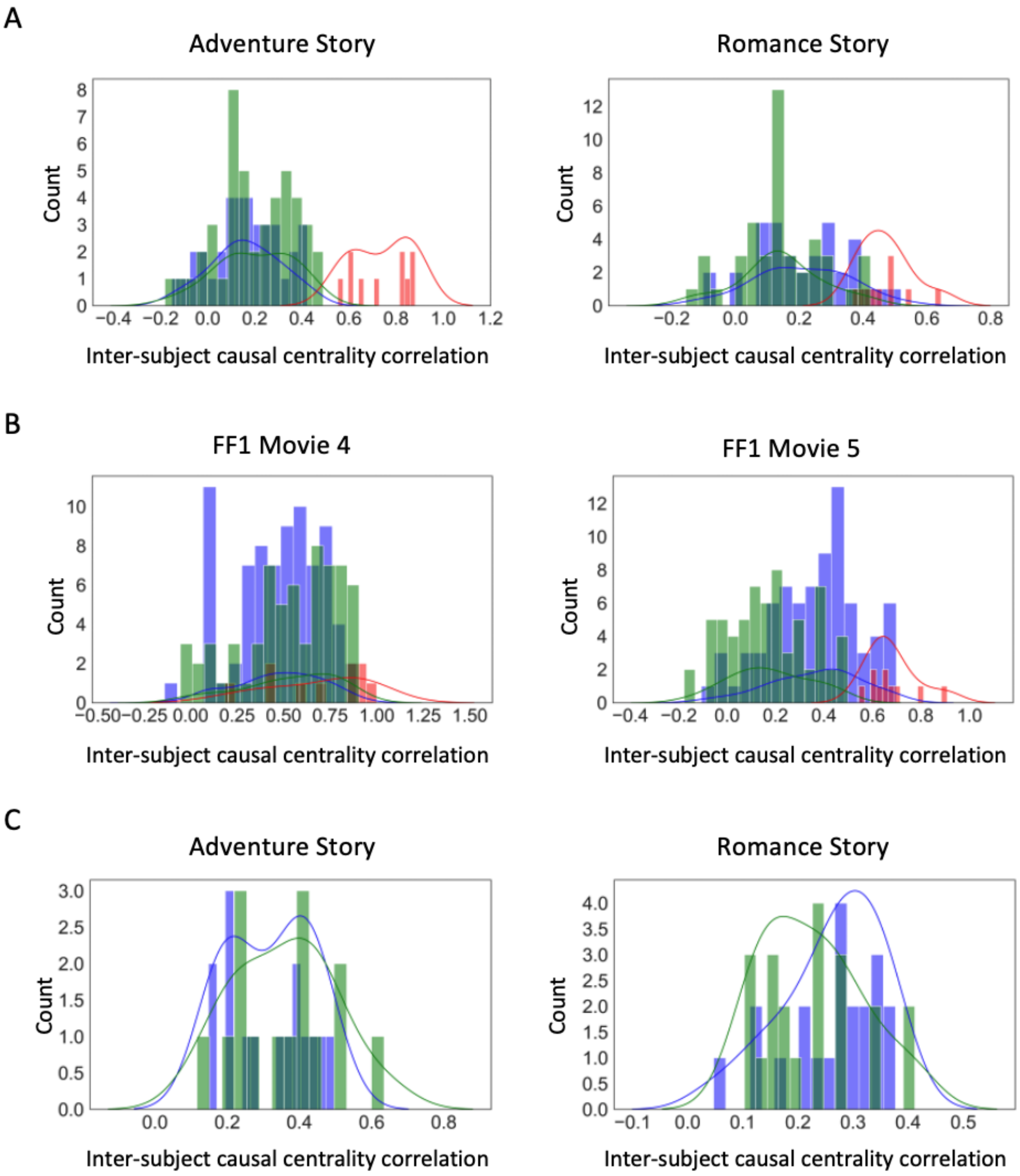

Supplementary Fig. S1| GPT-4 agent performance on identifying causally related events in narratives compared to human raters. A, *Left*: The similarity of causal centrality vectors among human raters (blue, n=9), among iterations of GPT4 agent's responses (red, n=5), and between the human raters and the GPT4 agent iterations (green) in one Adventure story path (story1020). The blue curve reflects inter-human reliability, the red curve reflects GPT-4's internal consistency, and the green curve reflects human–GPT-4 similarity. Overlap between the blue and green distributions indicates that GPT-4's causal-centrality estimates are as similar to human raters as human raters are to each other. **B,** The same three distributions were plotted for a short movie narrative (FF1 movie 4; left) and a long movie narrative (FF1 movie 5; right; Humans n=13, GPT iterations = 5). GPT-4's causal-centrality estimates closely matched human ratings for the short movie and fell within the human-human reliability range for the long movie. **C,** The two distributions in the plot (left) compares the predictability on 15 participants'

event memory across 3 Adventure story paths using the causal centrality derived from human raters and that derived from GPT4 agent. Similarly, the two distributions in the plot (right) compare the predictability of 21 participants' event memory across three *Romance* story paths. In both cases, GPT-4–derived causal-centrality predicted memory performance comparably to human-derived measures.

Supplementary Note 2. A third story excluded due to incomplete recollections

In addition to the two CYOA stories reported in the main paper, we wrote a third CYOA story from the mystery genre. Participants' (N=331; Free N=97, Yoke N=104, Passive N=130) recalls were collected, processed, and analyzed under the same procedures. However, a large proportion of participants skipped a sizable portion of the story during their recall; we speculate that this occurred due to the unusual structure of the narrative.

Specifically, this mystery story featured a protagonist discovering a potential murder, wherein the murderer's identity is revealed by three clues in three separate rooms of a spooky augmented-reality house. By word count and number of events, the three rooms constitute approximately 60% of the story. However, we found that more than half of Passive participants (52.4%) skipped or drastically summarized these parts of the story during their recall, e.g., referring to the entirety of the "rooms" plot in a sentence or two; these 'room-skippers' occurred at a rate of 37.5% in the Yoked condition, and 32% in the Free condition. In short, many participants seemed to think it sufficient to merely mention the "outcome" of each room (the recovered clue, which was in each case a single letter of the alphabet), without going into any detail about what actually happened in the rooms that led to the discovery of the clues.

Given that a significant portion of the collected sample in each condition skipped a large portion of the story in their recall, and that this factor appeared to interact with agency, we dropped this story from further analyses.

Supplementary Note 3. Agency did not improve overall recall

Participants' overall recall for the narrative were calculated as the number of events recalled divided by the total number of events in the story-path the participant read, i.e., the proportion of events recalled. In both stories, the Free participants recalled comparable proportion of events as the Yoked and Passive participants (Supplementary Figure S2). This suggest that agency does not improve recall for a narrative when it contains dense events that are interconnected through various relations.

For the Romance story, we additionally recorded the reading time for each subject and measured individual engagement over the course of their reading via a 13-item modified version of the Narrative Transportation scale[3]. There were no significant differences across the three agency conditions on participants' transportation score ($F(2,123) = 1.82$, $p = 0.167$), average reading time per story sentence ($F(2,123) = 0.41$, $p = 0.668$), or the overall reading time for the entire story-path they experienced ($F(2,123) = 0.42$, $p = 0.656$). These results suggest that the overall engagement for the story remained roughly the same across the three agency conditions.

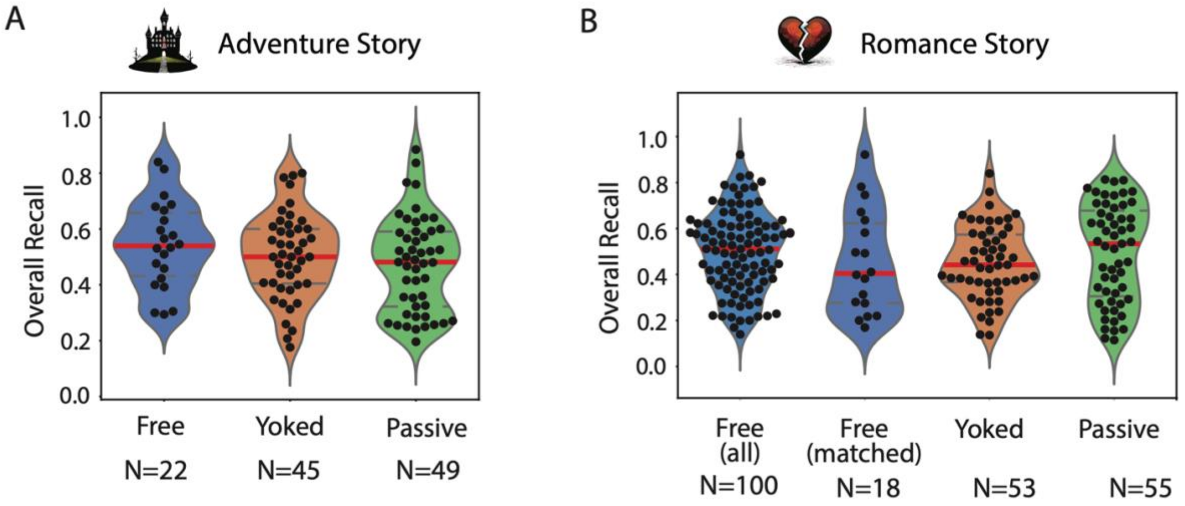

Supplementary Figure S2| Overall recall performance comparison. A, Overall recall performance of participants in Adventure story. Each dot represents one subject's percentage of events recalled. **B,** Overall recall performance of participants in the Romance story. The full group of Free condition participants (N=100) is plotted alongside the sample of Free condition participants (N=18) whose story-paths were read by the Yoked and Passive condition participants.

Supplementary Note 4. Recall for choice and non-choice events

Memory for choice events is significantly reduced compared to the other events ($p < .001$). The choice events in our paradigm differ in nature from the other (non-choice) narrative events—choice texts were presented in different fonts, sizes, styles, and served a different function from the non-choice events during the reading. Moreover, they describe specific actions that participants can select and thus are much shorter (around 5 words) than non-choice events (~40–50 words), which are usually extended narrative segments containing rich descriptive and action content (e.g., non-choice event: "*One girl breaks away from the cluster and skips over to you. You've seen her at one or two parties. She points at you, grinning. "You're Adam's girlfriend." Your throat catches. "Ex." "Oh." Her eyes twinkle. "I know a secret." She leans closer. The smell of beer shoots up your nose. "Do you want to know?";* choice event: "*Don't tell me.*")

The overall less information contained in the choice events potentially rendered them less likely to be recalled in absolute terms. Given the differences in propositional density between non-choice and choice events, we may use word count to serve as a pragmatic proxy for information load within each scored event. When recall is normalized by the number of words in each event, we see the opposite pattern: participants recall choice events *more often per word* than normal story events. This was true across all agency conditions, even for the Passive condition.

We compared recall score for non-choice versus choice events across the three agency conditions (Free, Yoked, Passive). Importantly, because Yoked and Passive participants read the story paths generated by Free participants, event content was matched across conditions; only the degree of perceived agency varied. For both stories, memory performance did not differ significantly across conditions (*Adventure*: $F(2,113) = 1.76$, $p = 0.177$; *Romance*: $F(2,205) = 1.80$, $p = 0.167$). Recall performance for non-choice events was significantly higher than for choice events across all conditions for both stories (*Adventure*: $F(1,113) = 430.23$, $p < .001$; *Romance*: $F(1,205) = 722.06$, $p < .001$). There

was a significant agency condition × event type interaction for the Romance story ($F(2, 205) = 12.77$, $p < .001$), and a trend for the *Adventure* story ($F(2, 113) = 2.76$, $p = 0.068$). For the *Romance* story, post-hoc comparisons for choice events revealed: Free > Yoked ($t = 2.43$, $p = 0.016$), Free > Passive ($t = 1.99$, $p = 0.048$).

We next compared recall by word performance for non-choice versus choice events across the three agency conditions (Free, Yoked, Passive). Across the two stories, we did not observe consistent differences across conditions (*Adventure*: $F(2,113) = 1.37$, $p = 0.258$; *Romance*: $F(2,205) = 3.48$, $p = 0.033$). Recall by word performance for choice events was significantly higher than for non-choice events (*Adventure*: $F(1,113) = 165.41$, $p < .001$; *Romance*: $F(1,205) = 459.21$, $p < .001$). There was a significant agency condition × event type interaction for the Romance story ($F(2, 205) = 4.45$, $p = 0.013$), but not for the *Adventure* story ($F(2,113) = 1.85$, $p = 0.163$). For the *Romance* story, post-hoc comparisons for choice events revealed: Free > Yoked in recall per word ($t = 2.46$, $p = 0.015$), Free > Passive in recall per word ($t = 2.09$, $p = 0.038$).

Together, these results showed that the choice events were better recalled than non-choice events when we normalized recall performance by word count. This is consistent with the idea that making a choice could improve memory—even in the passive condition, the simple physical action of clicking a choice button but not having an actual ability to choose otherwise could still improve memory. Further, the enhanced memory for choice events was most prominent in the free condition, where subjects had the most agency. This suggests that perceived agency may further boost recall per word beyond the physical action of selection (clicking or dragging a button).

The result that Free participants recalled choice events better than matched participants in Yoked and Passive conditions echoes classic findings in the study of human memory: the enactment effect, wherein memory is enhanced when actions are performed oneself, as opposed to when observed [4]; and the generation effect, wherein memory is enhanced when one generates information oneself, as opposed to when it is merely presented [5].

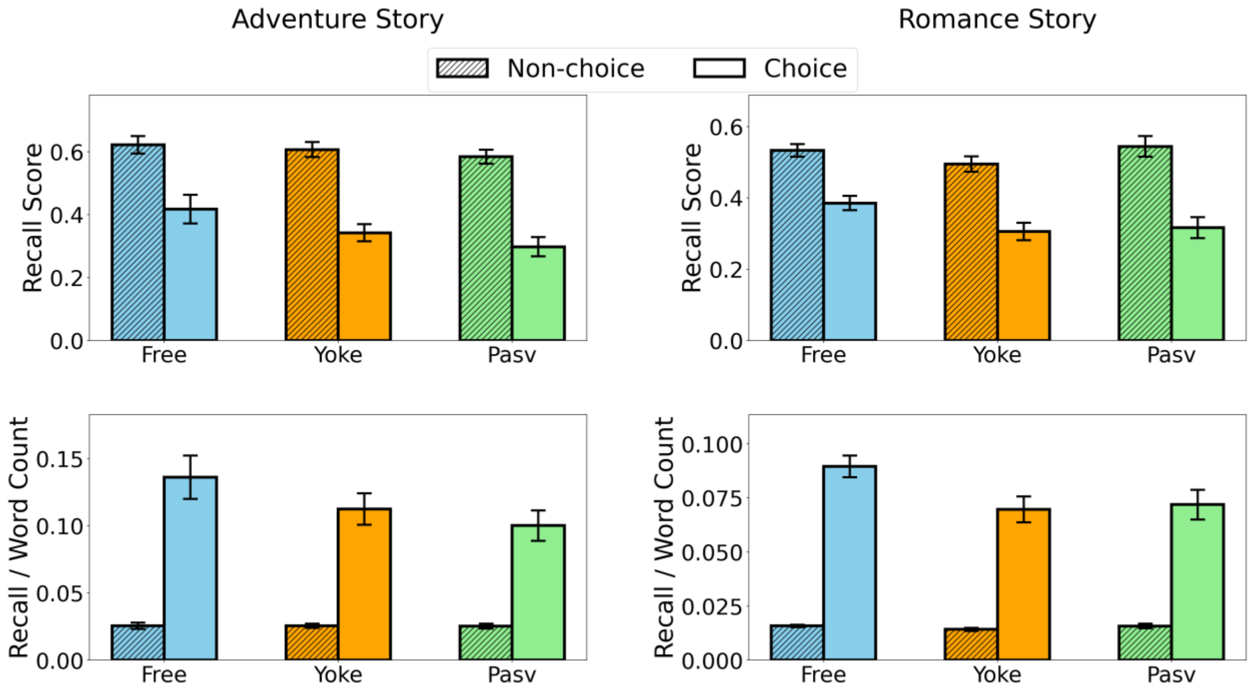

Supplementary Figure S3| Recall and recall per word split by choice and non-choice event. The figure displays two measures organized in rows: Recall Score (top row) and Recall per Word (bottom row). For each measure, the left column shows data from the Adventure story (Free: n=22, Yoked: n=45, Passive: n=49), and the right column shows data from the Romance story (Free: n=100, Yoked: n=53, Passive: n=55). Each panel shows six bars representing the three agency conditions (Free: sky blue, Yoked: orange, Passive: light green) × two event types (Non-choice: hatched bars with diagonal slashes, Choice: solid bars). Error bars represent standard error of the mean across subjects. The figure demonstrates that choice events showed higher recall per word than non-choice events across all conditions and both stories, with agency condition differentially affecting memory for choice versus non-choice events in the Romance story.

Supplementary Note 5. Agency has dissimilar effects on recognition and recall.

We designed a 4-choice recognition memory test for the shared story sections of the *Romance* story. This test was intended to probe event-specific memory, as opposed to general comprehension; as such, to the extent possible, each question covered material unique to one event (validated across iterations with naïve participants). A result of this requirement was that the recognition memory test likely assessed substantially different aspects of memory from the free recall test used in all prior sections.

The test consisted of 89 questions, each of which was a short excerpt (1-2 sentences) from the story with key words or phrases left blank; participants were provided four options to fill in the blank. The passages were presented in chronological order, in order to avoid confusion. Each participant answered 76 questions on the shared story sections (39 shared events; 2-3 questions per event), and 6-7 questions on the ending that they read (6 questions on 3 events in ending 1; 7 questions on 3 events in ending 2). In *Romance* story experiment, participants first read through the story, then completed several questionnaires, then performed written recall of the story, and lastly were presented with the recognition memory test.

The recognition memory test questions were designed to probe memory for only one event/passage at a time, so as to allow generation of a timecourse of event memory (corresponding to the manner in which we analyzed the free recall data). This sharply constrained which words/phrases could be selected for the test (to be left blank). We could only choose words/phrases that depended on memory of the currently presented event/passage and no other events. For example, it would not be acceptable to blank out the name of a character, because that information would also appear elsewhere in the story. Furthermore, the selection of foils needed to be done carefully, as many words/phrases have high frequency biases in a given context; for example, "salt and <blank> shakers" has a very obvious completion and most foils would be rejected even by participants with no memory of that event. Thus, we iteratively tested the recognition memory test in a separate group of participants. The objective was to ascertain, for each test question, whether participants would select the correct answer above chance-level, despite never having read the story. Each set of recognition memory questions was tested on 25-35 naïve participants (Total N=329). Questions for which recognition accuracy was higher than 50% were removed from the test set, and new questions were developed to replace them; the entire set was then re-tested on another sample of naïve participants. Five iterations of this process were completed to ensure the average accuracy across the questions was below chance level (final naïve group accuracy across 89 questions: N=20, M=0.24, SE=0.02). However, note that a few individual questions still remained above-chance. See *Data Availability* for the full set of questions.

Overall recognition performance was significantly different across conditions [$F(2,123) = 4.06$, $p = 0.020$]; post-hoc analyses showed that Free participants (mean = 23.9) had lower performance than Passive (mean = 27.3; $p = 0.027$), while no difference was found between Free and Yoked (mean = 25.4) participants ($p = 0.447$). Neither semantic centrality nor causal centrality were consistently related to recognition memory performance, and agency did not modulate correlations between semantic centrality and recognition memory ($p > 0.1$), nor between causal centrality and recognition memory ($p > 0.2$).

Recognition and recall performance were positively correlated across participants [Free ($r = 0.22$), Yoked ($r = 0.48$), and Passive ($r = 0.21$)]. Correlations of recall vs. recognition event vectors within subject were significantly different across conditions ($p = 0.012$), with Free participants showing reduced correlations relative to Yoked (Free mean $r = -0.01$, Yoked mean $r = 0.12$, $p = 0.009$).

In sum, recognition and recall tests showed different behavior profiles in this experiment. While recall performance was not different between agency conditions, recognition performance was; both semantic and causal centrality predicted recall in all conditions, but they did not consistently predict recognition; and agency modulated the relationship between semantic centrality and recall, but had no impact on the relationship between semantic centrality and recognition. At the event level, recognition and recall were most different from each other in the Free condition.

It is important to clarify the differences between the recall and recognition tests in the context of this study. Our goals for the recognition memory test required that each test question be written to query *only* information unique to the event of interest; this was because we ultimately needed a separate memory score for each event, as we had from free recall. Thus, recognition memory questions had to be extremely temporally precise,

which often resulted in an emphasis on small details. For example, participants might be asked to identify what specific childhood memory was disrupted by an alarm in the narrative, with options such as a drawing lesson, a golden retriever, a toy bunny, or a camping experience. It is also worth noting that such a test is difficult to create because humans are quite good at selecting the correct answer from a list of options even when they have not read the story, and thus question design requires multiple iterations of testing with naïve participants (see Methods). In contrast, the recall test simply consisted of a retelling of the narrative, and thus included both details and broader statements that could span multiple events. For example, the recall sentence "…finally they go grab a coffee where the woman has an awkward interaction with the barista Ryan" refers to four non-consecutive events. Beyond overall memory performance, agency did not modulate any other metrics of memory when using the recognition test scores (e.g., semantic centrality, causal centrality). This is consistent with a recent study suggesting that a sense of agency may not improve recognition memory[6]. The differences between recall and recognition tests are important methodological considerations for future studies which use narratives or other lifelike event sequences; recognition tests are sharply constrained in their ability to assess event comprehension, and instead should be employed when memory for fine and temporally limited details are of primary interest.

Supplementary Note 6. Consequences of having one's choices denied.

The number of choices granted to each Yoked participant across their story-path varied depending on the degree of alignment between their choices and those of their Free counterparts. In both stories, there was considerable variation in the percentage of choices granted in Yoked participants. *Adventure*: range=.29-.83; mean=.53; SE=.02. *Romance*: range=.45-.83; mean=.62; SE=.01. (Supplementary Figure S4)

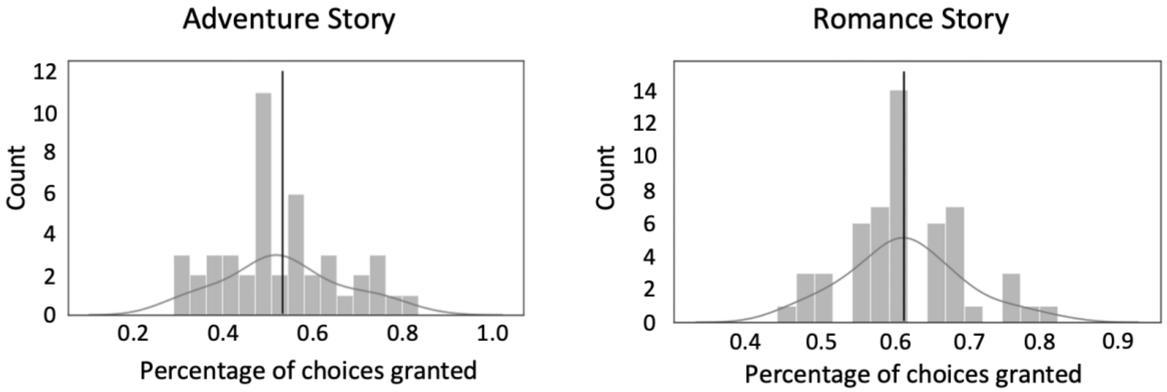

Supplementary Figure S4| Percentage of choices granted to the Yoked participants. Distributions of overall percentage of choices granted for Yoked participants in the Adventure story (mean=0.53; left) and in the Romance story (mean=0.62; right).

We examined whether having choices granted vs. denied had consequences for event memory. We separately computed the average recall scores of choice-denied and choice-granted events for each Yoked participant, and of the exact same events for their Free counterparts. For both stories, recall performance was lower for choice-denied events in Yoked participants compared to their Free counterparts (*Adventure:* $p = 0.015$; *Romance:* $p = 0.017$, two-sample t-test), while recall performance for choice-granted events was not different ($ps > 0.2$; Supplementary Figure S5).

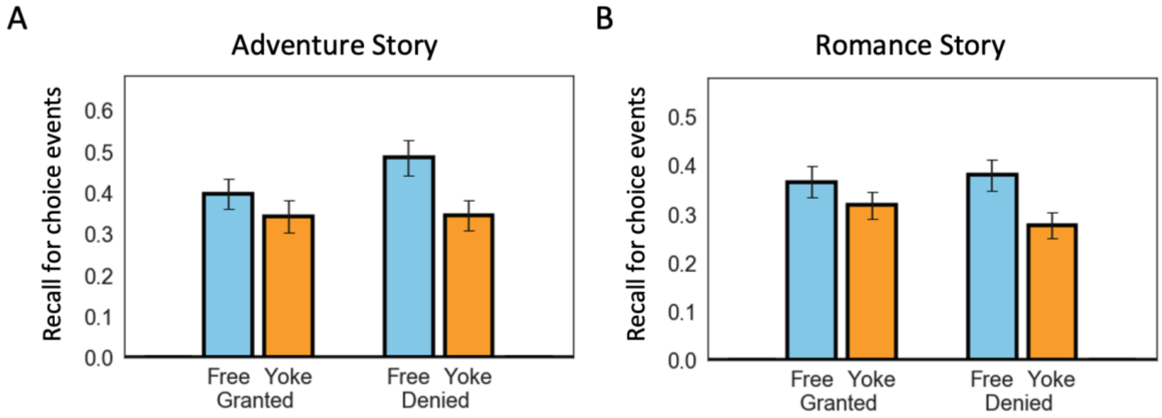

Supplementary Figure S5| Recall performance for choice events that are denied in the yoke condition vs. their free condition counterparts. A, Adventure story participants' recall performance for granted choice events (left two bars), and for the denied choice events (right two bars). Each Yoked subject has a different collection of choice events denied (Free: n=44, Yoked: n=44). We computed each Yoked subject's average recall for events where their choice was denied and computed the average recall of the same events in their matching Free counterparts (where these choice events were always granted). This ensured that the events we compared were exactly the same. The same analysis was performed for granted choice events in Yoked participants and in their Free counterparts. Events for which Yoked participants were denied their choice were less well recalled compared to their Free counterparts, for whom choices were granted; meanwhile, recall for the granted choice events in Yoked participants was comparable to that of their Free counterparts. **B,** Same as A, for the Romance story (Free: n=51, Yoked: n=51). In both A-B, data are presented as mean values +/- SEM.

We quantified each Yoked participant's tendency to recall or forget denied events as the Pearson correlation between a binary vector (denied/granted = 0/1) and their event-by-event recall vector (0/1). One Adventure participant and two Romance participants were excluded from this analysis for recalling none of the choice events. Correlations centered near zero in both stories (Adventure: $t(43) = -0.076$, $p = 0.939$; Romance: $t(50) = 1.53$, $p = 0.133$; two-sample t-test), indicating substantial individual differences.

To probe these differences, we classified Yoked participants by whether they tended to forget denied events or recall them (Adventure: forget-denied, N = 21; recall-denied, N = 20. Romance: forget-denied, N = 29; recall-denied, N = 22). For denied events, the forget-denied subgroup showed reduced recall relative to Free (Adventure: $t(40) = -2.68$, $p = 0.011$; Romance: $t(56) = -3.66$, $p < .001$), whereas the recall-denied subgroup did not differ from Free (Adventure: $t(38) = -1.10$, $p = 0.279$; Romance: $t(42) = 1.16$, $p = 0.252$). Thus, the overall reduction in Yoked recall for denied events was driven by participants who tended to forget denied events.

These results suggested that the overall reduced memory for choice-denied events among Yoked subjects, compared to their Free counterparts, was likely driven by the sub-sample that tended to forget choice-denied events. These subjects had a significantly greater memory reduction for choice-denied events than the memory improvement in subjects who tended to recall these events, contributing to the overall reduced memory in Yoked subjects for the choice-denied events compared to Free.

Supplementary Note 7. Agency effects not as a function of percentage of choices granted.

Since the Yoked subjects had a considerable variation in the percentage of choices granted, could the various memory effects of agency be a function of the percentage of choices granted?

We examined whether the percentage of choices granted across the whole story-path was correlated with overall recall performance, recall of choice-granted events, or recall of choice-denied events. We found no consistent relationship between the percentage of choices granted and these memory scores across the two stories. All *p*s>.1 except *Adventure*: percentage of choices granted vs. choice-denied events' recall r(43)=-.309, p=.039.

We then investigated whether Yoked participants with a higher percentage of granted choices had event-by-event recall more similar to their Free counterparts in the shared sections of the *Romance* story; however, no significant correlation emerged. r(51)=.100, p=.476. We also examined whether Yoked participants with a lower percentage of granted choices had event-by-event recall more similar to their Passive counterparts in the shared sections of the *Romance* story; however, no significant correlation emerged. r(51)=.117, p=.404.

Next, we tested whether the percentage of choices granted to Yoked participants was predictive of the magnitude of either the semantic centrality effect or the neighbor encoding effect on memory. We found that the percentage of choices granted to Yoked participants did not correlate with these two effects on memory across the two stories (all ps>.3).

Furthermore, we tested whether the percentage of choices granted to Yoked participants affected their tendency to either forget or recall choice-denied events. One subject from the *Adventure* story and two from the *Romance* story were dropped from this correlation analysis due to not recalling any choice events. For both stories, there was a positive correlation between the percentage of choices granted to participants and their tendency to forget choice-denied events, i.e., participants who were granted a higher percentage of choices tended to forget more of the choice-denied events. *Adventure*: r(42) = .335, p = .026. *Romance*: r(49) = .137, p = .337. These findings implied that in contexts lacking full agentive control, a higher frequency of granted choices (higher overall agency) was associated with a more pronounced memory reduction for denied choices (choice-denied events).

Overall, these results suggest that the effects of agency on memory is not a function of the percentage of choices granted in the Yoked subjects. This implies that in a context lacking full agentive control, perceived agency and their effects on memory could vary across individuals in non-systematic ways. The one exception is that with more control in such agency-uncertain contexts, the more one has reduced recall for the agency-denied events.

Supplementary Note 8. Semantic centrality and Causal centrality in all conditions: individual data.

Semantic centrality values generally ranged from 0.02–0.25 for the Adventure story and from 0–0.30 for the Romance story. (Supplementary Figure S6)

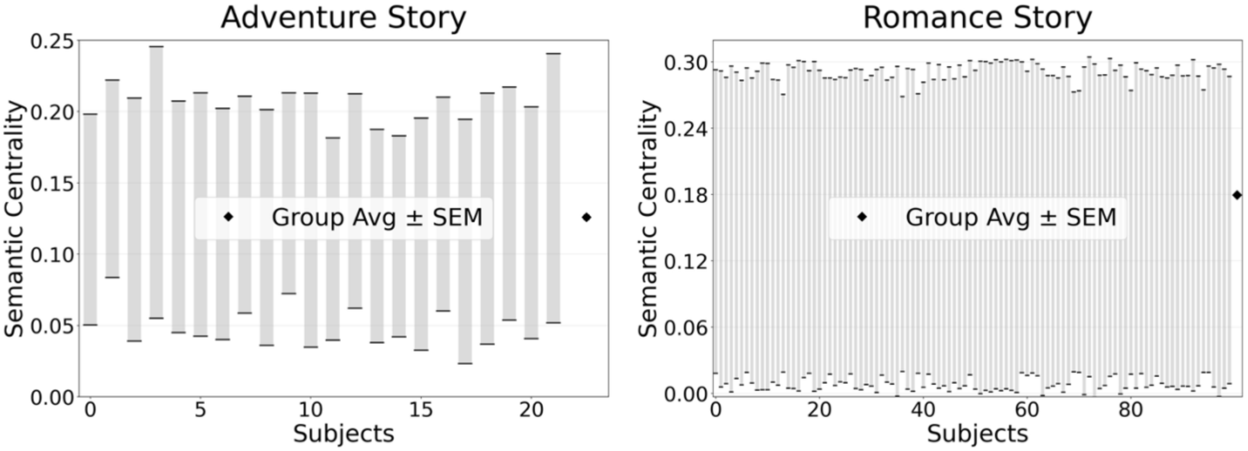

Supplementary Figure S6| Range of semantic centrality values per subject. The figure displays the range of semantic centrality values for individual subjects in both the Adventure Story (left panel, n=22) and Romance Story (right panel, n=100). Each vertical light gray bar represents one subject, with black horizontal lines capping the minimum and maximum semantic centrality values for that subject across all events in their story path. The black diamond marker with error bars positioned to the right of the individual subject data shows the group average ± standard error of the mean (SEM) across all subjects and events for each story (Adventure: mean = 0.126, SEM = 0.001; Romance: mean = 0.180, SEM = 0.001). This visualization demonstrates the variability in semantic centrality values both within individual subjects (range) and across the group (group average with SEM) for both narrative contexts.

Semantic centrality significantly predicted recall in all three conditions (one-sample t-tests against zero). *Adventure*: Free, $t(21) = 5.58$, $p < .001$, Cohen's $d = 1.19$, 95% CI of difference = [.14, .31]; Yoked, $t(45) = 16.23$, $p<.001$, Cohen's $d = 2.39$, 95% CI of difference = [.29, .37]; Passive, $t(48) = 12.68$, $p<.001$, Cohen's $d = 1.81$, 95% CI of difference = [.26, .36]. *Romance:* Free, $t(17) = 6.11$, $p < .001$, Cohen's $d = 1.44$, 95% CI of difference = [.10, .20]; Yoked, $t(52) = 19.02$, $p<.001$, Cohen's $d = 2.61$, 95% CI of difference = [.20, .25]; Passive, $t(54) = 20.00$, $p<.001$, Cohen's $d = 2.70$, 95% CI of difference = [.24, .29].

Causal centrality significantly predicted recall in all three conditions (one-sample t-tests against zero). *Adventure*: Free, $t(21) = 4.60$, $p<.001$, Cohen's $d = 0.98$, 95% CI of difference = [.10, .27]; Yoked, $t(45) = 5.63$, $p<.001$, Cohen's $d = 0.83$, 95% CI of difference = [.10, .22]; Passive, $t(48) = 4.92$, $p<.001$, Cohen's $d = 0.70$, 95% CI of difference = [.07, .18]. *Romance:* Free, $t(17) = 8.61$, $p < .001$, Cohen's $d = 2.03$, 95% CI of difference = [.16, .27]; Yoked, $t(52) = 14.40$, $p<.001$, Cohen's $d = 1.98$, 95% CI of difference = [.20, .27]; Passive, $t(54) = 23.99$, $p<.001$, Cohen's $d = 3.23$, 95% CI of difference = [.22, .27].

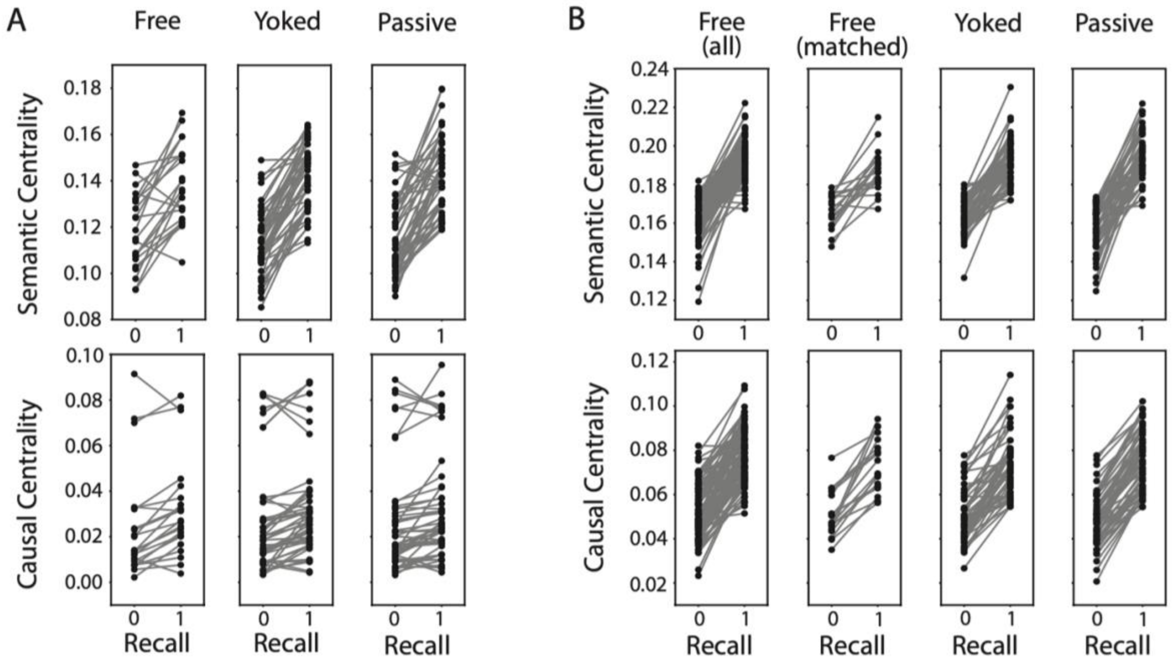

Supplementary Figure S7| Semantic and causal centrality effects on memory plotted for individual participants. A, The top panel shows connected-dot line plots for the Adventure story, illustrating the effect of semantic centrality on memory. In each sub-plot, the x-axis shows recall score of 0 (unrecalled) and 1 (recalled), and the y-axis shows the average semantic centrality score across the unrecalled and recalled events; each subject is represented as a connected line. Across all participants in the three conditions, events with higher semantic centrality were more likely to be recalled. The bottom panel shows the same information for the effect of causal centrality on memory. **B,** Same as A, for the Romance story. The total number of Free participants (N=100) is plotted alongside the sample of Free participants (N=18) whose story paths were matched with the Yoked and Passive participants.

Story	Condition	Network	Mean r [95% CI]	Test statistic	Cohen's d
Adventure	Free	Semantic	0.223 [0.140, 0.306]	t(21) = 5.58, p < 0.001	1.19
Adventure	Yoked	Semantic	0.323 [0.282, 0.363]	t(44) = 16.07, p < 0.001	2.40
Adventure	Passive	Semantic	0.308 [0.259, 0.356]	t(48) = 12.68, p < 0.001	1.81
Romance	Free	Semantic	0.146 [0.096, 0.197]	t(17) = 6.11, p < 0.001	1.44
Romance	Yoked	Semantic	0.221 [0.198, 0.245]	t(52) = 19.02, p < 0.001	2.61
Romance	Passive	Semantic	0.265 [0.239, 0.292]	t(54) = 20.00, p < 0.001	2.70
Adventure	Free	Causal	0.187 [0.102, 0.271]	t(21) = 4.60, p < 0.001	0.98
Adventure	Yoked	Causal	0.159 [0.100, 0.217]	t(44) = 5.45, p < 0.001	0.81
Adventure	Passive	Causal	0.126 [0.075, 0.178]	t(48) = 4.92, p < 0.001	0.70
Romance	Free	Causal	0.213 [0.161, 0.266]	t(17) = 8.61, p < 0.001	2.03
Romance	Yoked	Causal	0.236 [0.203, 0.269]	t(52) = 14.40, p < 0.001	1.98
Romance	Passive	Causal	0.244 [0.224, 0.265]	t(54) = 23.99, p < 0.001	3.24

Table S1. Full statistics for one-sample t-tests against zero of semantic and causal centrality effects on event recall, in each condition (Free, Yoked, Passive) for the Adventure and Romance stories. The effect of semantic centrality (semantic influence) on memory was computed as the Pearson correlation between semantic centrality and event-by-event recall (1 = remembered, 0 = forgotten) for each participant; the same procedure was used for causal centrality (causal influence). Mean r reflects the group-average within-participant correlation. All p-values are two-sided. Effect size is Cohen's d. n = 22 (Adventure Free), 45 (Adventure Yoked), 49 (Adventure Passive), 18 (Romance Free), 53 (Romance Yoked), 55 (Romance Passive).

Supplementary Note 9. Semantic and causal centrality separately predict event recall.

In prior work, it was found that semantic centrality and causal centrality tend to be correlated in narratives (r = 0.28); mixed-effects logistic regression analyses showed that each factor predicted recall while controlling for the other [7]. In the current study, participants generated 22 *Adventure* and 100 *Romance* story-paths. By chance, there were a fair number of story-paths for which semantic and causal centrality were uncorrelated or negatively correlated. This allowed us to examine the contributions of each factor to memory without a combined model.

In the *Adventure* story, for 3 of the 22 story-paths, correlations between semantic and causal centrality were in the range $-0.1 < r < 0.1$ (all story-paths mean = 0.203, range: -0.191 to 0.354). From among the participants reported above, 17 in total read these 3 stories; semantic centrality and recall were correlated at r = 0.258, while causal centrality and recall were correlated at r = 0.126. In the *Romance* story, for 14 of the 100 story-paths, correlations between semantic and causal centrality were in the range $0 < r < .1$ (all story-paths mean = 0.147, range: 0.021 to 0.237). 41 participants in total read these 14

stories; semantic centrality and recall were correlated at r=0.196, while causal centrality and recall were correlated at r = 0.285. See Supplementary Table S2.

Overall, these results indicate that semantic centrality and causal centrality can separately predict recall performance in narratives.

story	story-path	subID	semantic effect	causal effect	sem-cent r	cond	conds matched
adventure	3	sub3_1002	0.404	-0.282	-0.075	free	y
adventure	3	to3_sub2026	0.005	0.387	-0.075	yoke	y
adventure	3	to3_sub2033	0.397	0.127	-0.075	yoke	y
adventure	3	to3_sub2037	0.248	-0.129	-0.075	yoke	y
adventure	3	to3_sub3001	0.039	-0.264	-0.075	pasv	y
adventure	3	to3_sub3028	0.264	-0.127	-0.075	pasv	y
adventure	3	to3_sub3044	0.344	-0.132	-0.075	pasv	y
adventure	2	sub2_1001	0.34	0.401	0.083	free	y
adventure	2	to2_sub2023	0.525	0.084	0.083	yoke	y
adventure	2	to2_sub2040	0.435	0.258	0.083	yoke	y
adventure	2	to2_sub3027	0.385	0.303	0.083	pasv	y
adventure	2	to2_sub3042	0.268	0.176	0.083	pasv	y
adventure	13	sub13_1012	0.057	0.293	0.09	free	y
adventure	13	to13_sub2000	0.267	0.298	0.09	yoke	y
adventure	13	to13_sub2058	0.491	0.276	0.09	yoke	y
adventure	13	to13_sub3038	0.074	0.331	0.09	pasv	y
adventure	13	to13_sub3046	-0.15	0.138	0.09	pasv	y
romance	40	sub40_4040	0.134	0.458	0.021	free	y
romance	40	to40_sub6008	0.096	0.305	0.021	pasv	y
romance	40	to40_sub6022	0.019	0.298	0.021	pasv	y
romance	40	to40_sub6024	0.127	0.171	0.021	pasv	y
romance	40	to40_sub5021	0.131	0.349	0.021	yoke	y
romance	40	to40_sub5025	0.282	0.45	0.021	yoke	y
romance	37	sub37_4037	0.145	0.236	0.041	free	y
romance	37	to37_sub6005	0.253	0.266	0.041	pasv	y
romance	37	to37_sub6015	0.205	0.323	0.041	pasv	y
romance	37	to37_sub6016	0.16	0.341	0.041	pasv	y
romance	37	to37_sub5007	0.239	0.367	0.041	yoke	y
romance	37	to37_sub5008	0.211	0.064	0.041	yoke	y
romance	37	to37_sub5012	0.233	0.392	0.041	yoke	y
romance	37	to37_sub5026	0.102	0.438	0.041	yoke	y
romance	14	sub14_4014	-0.02	0.134	0.045	free	y
romance	14	to14_sub6007	0.254	0.28	0.045	pasv	y
romance	14	to14_sub6021	0.348	0.322	0.045	pasv	y

romance	14	to14_sub5010	0.134	0.31	0.045	yoke	y
romance	14	to14_sub5011	0.234	0.212	0.045	yoke	y
romance	14	to14_sub5020	0.091	0.296	0.045	yoke	y
romance	71	sub71_4071	0.146	0.393	0.083	free	y
romance	71	to71_sub6029	0.307	0.249	0.083	pasv	y
romance	71	to71_sub6041	0.206	0.373	0.083	pasv	y
romance	71	to71_sub5030	0.004	0.292	0.083	yoke	y
romance	71	to71_sub5038	0.145	0.431	0.083	yoke	y
romance	81	sub81_4081	0.272	0.152	0.091	free	y
romance	81	to81_sub6026	0.27	0.35	0.091	pasv	y
romance	81	to81_sub6035	0.15	0.296	0.091	pasv	y
romance	81	to81_sub6040	0.215	0.283	0.091	pasv	y
romance	81	to81_sub6050	0.231	0.254	0.091	pasv	y
romance	81	to81_sub5034	0.145	0.219	0.091	yoke	y
romance	81	to81_sub5044	0.243	0.369	0.091	yoke	y
romance	91	sub91_4091	0.153	0.201	0.055	free	n
romance	22	sub22_4022	0.371	0.167	0.069	free	n
romance	88	sub88_4088	0.233	0.278	0.071	free	n
romance	89	sub89_4089	0.216	0.205	0.073	free	n
romance	35	sub35_4035	0.274	0.394	0.077	free	n
romance	34	sub34_4034	0.308	0.357	0.08	free	n
romance	12	sub12_4012	0.199	0.111	0.087	free	n
romance	87	sub87_4087	0.389	0.099	0.09	free	n
romance	75	sub75_4075	0.183	0.204	0.093	free	n

Table S2. Semantic and causal centrality separately predict event recall when they are uncorrelated.

Supplementary Note 10. Relationships between memory divergence, semantic influence, and the neighbor encoding effect.

We plotted the relationship between participants' memory divergence, semantic influence on memory, and neighbor encoding effect. Results showed that the higher one's memory divergence from the group mean, the less the semantic influence on memory, and the more the neighbor encoding effect.

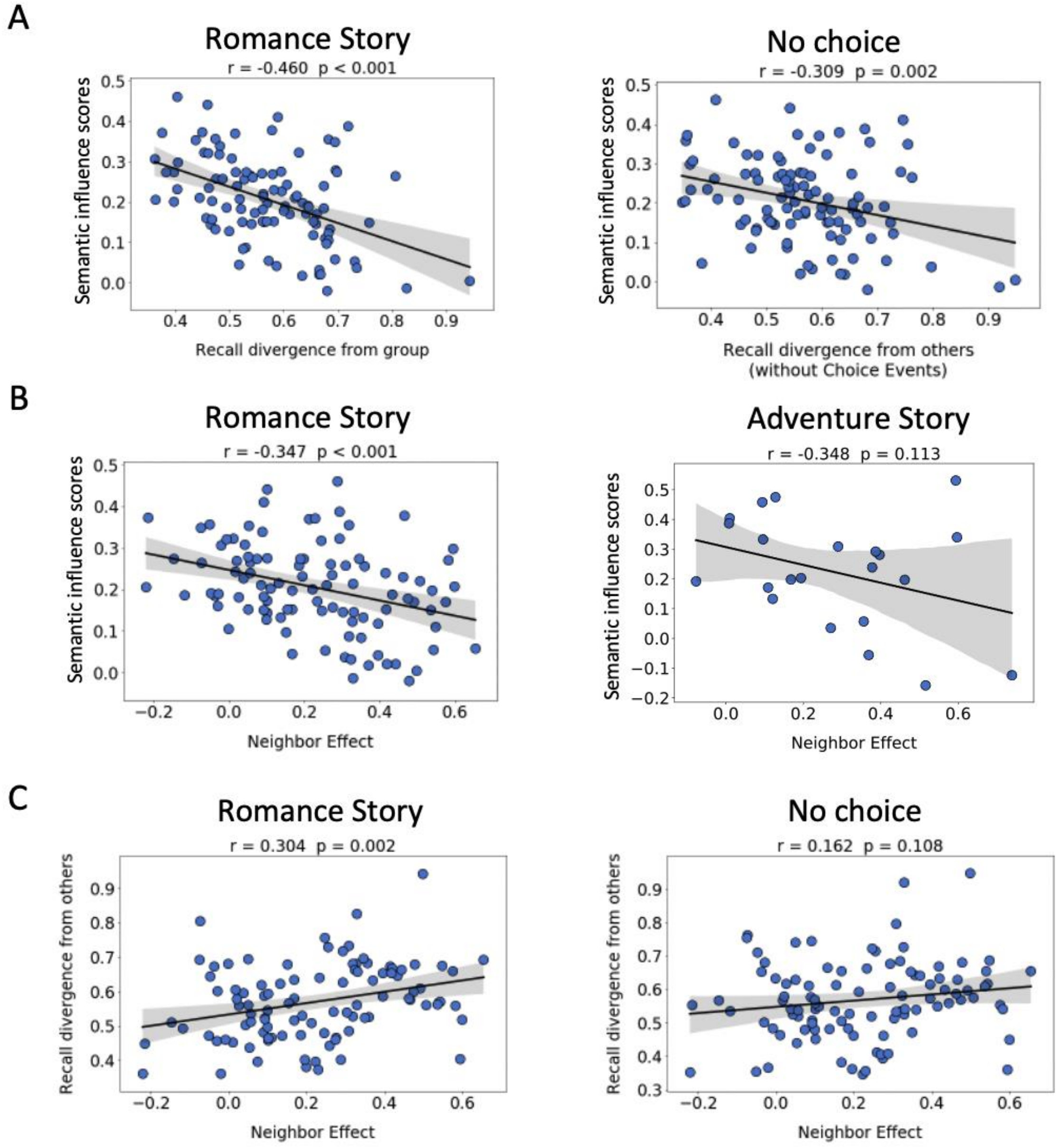

Supplementary Fig. S8| Relations between the multiple effects of agency on memory. A, The scatter plot (left) shows the relationship between participants' recall divergence from the group mean (x-axis) and their semantic influence score (y-axis) using the full group of 100 free condition participants in the Romance story shared sections (64 events). The more a subject's recall deviated from the group mean, the less well semantic centrality predicts the subject's memory. The scatter plot (right) shows the same relationship but using only the 49 non-choice events from the Romance story shared sections. **B,** The scatter plot (left) shows the relationship between participants' neighbor encoding effect (x-axis) and their semantic influence score (y-axis) using the full group of 100 Free participants in the Romance story. The more a subject's event memory can be predicted by its neighbors, the less semantic centrality can predict the subject's memory. Similarly, the scatter plot (right) shows the same relationship but using the 22 Free condition participants in the Adventure story. **C,** The scatter plot (left) shows the relationship between participants' neighbor encoding effect (x-axis; computed on the entire story) and their recall divergence from the group mean (y-axis; computed on the 64 events over the shared sections) using the full group of 100 Free participants in the Romance story. The more a subject's event memory can be predicted by its neighbors, the more the subject's event memory tended to deviate from the group mean. Similarly, the scatter plot (right) shows the same

relationship, but with the recall divergence score computed on the 49 non-choice events from the shared story sections.

Supplementary Note 11. Individual variability in choices and recall for the selected 18 Free participants not different from the full Free sample.

We plotted the selected 18 Free participants' mean ISC against the full distribution of the 100 Free participants' pairwise ISC to demonstrated that the selected subset of Free participants was not significantly different from the full Free sample.

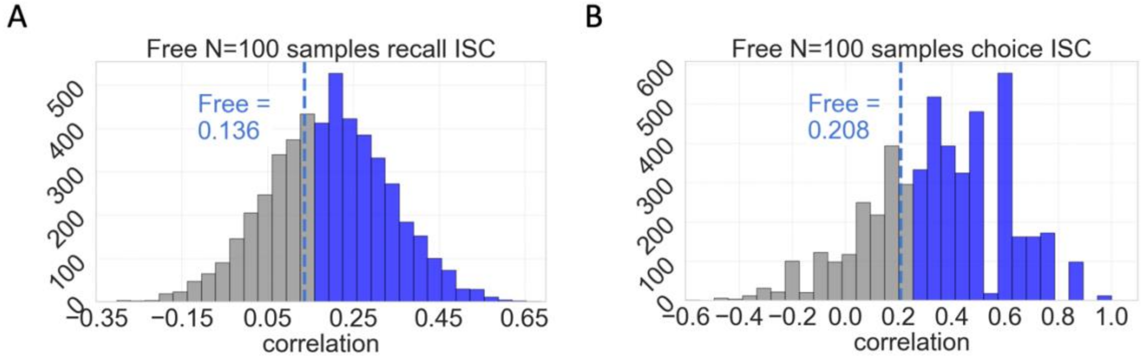

Supplementary Fig. S9| Recall and choice ISC distribution of full Free sample (N=100) with mean for selected N=18. A, Distribution of recall ISC values across all 100 Free participants, calculated from the 64 shared events in the Romance story. The vertical blue dashed line marks the mean recall ISC ($r = 0.136$, $p > 0.1$) for the 18 selected Free participants whose story paths were assigned to the Yoke and Passive conditions. Samples with greater group mean than the selected Free subjects were colored blue. **B,** Distribution of choice ISC values across all 100 Free participants, calculated from the 15 choice events in the shared sections of the Romance story. The vertical blue dashed line marks the mean choice ISC ($r = 0.208$, $p > 0.1$) for the same 18 Free participants. Samples with greater group mean than the selected Free subjects were colored blue.

Supplementary Note S12. Free participants' increased recall ISC not driven by greater choice ISC.

To control for the choice ISC between the Free and Yoked group when comparing their memory ISC, we draw 10,000 samples of 18 Yoked participants, each matching story paths and choice ISC to the 18 Free participants. Results showed that the Free participants still had significantly greater recall ISC than the Yoked participants, suggesting that the Free condition's recall ISC is not driven by its greater choice ISC.

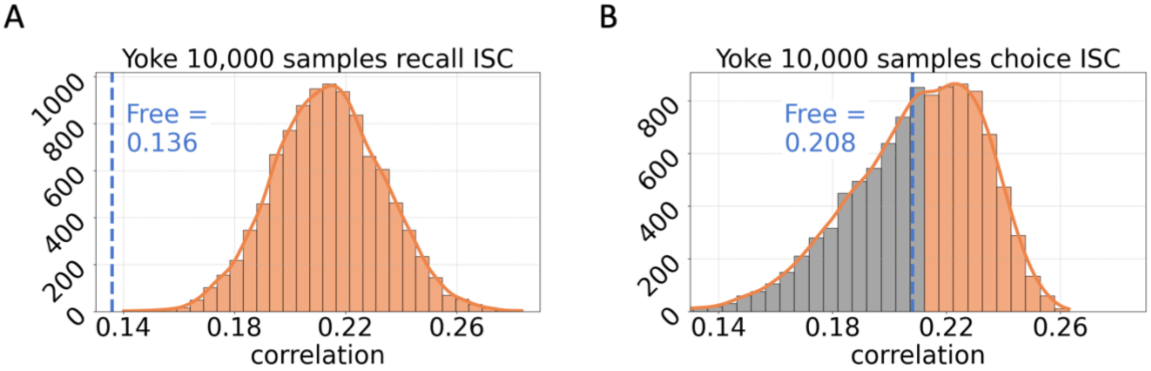

Supplementary Fig. S10| Recall ISC distribution for 10,000 Yoked samples matched on choice ISC to the selected 18 Free participants. A, Distribution of mean recall ISC for the 10,000 yoked samples (orange histogram), each matching the selected Free participants' story paths and choice ISC. The blue dashed line marks the selected Free participants' mean recall ISC ($r = 0.136$, $p = 0.000$). Recall ISC was calculated from the 64 shared story events in the Romance story. Samples with greater group mean than the selected Free subjects were colored orange. **B,** Distribution of mean choice ISC for the same 10,000 yoked samples, with the blue dashed line marking the 18 selected Free participants' mean choice ISC (mean $r = 0.208$, $p = 0.423$). Choice ISC was calculated from the 15 shared choice events in the Romance story. Note that the choice ISC was used as selection criteria to draw samples from the Yoked condition with matching choice behaviors to the 18 Free participants. Samples with greater group mean than the selected Free subjects were colored orange.

Supplementary Note 13. Neighbor encoding effect is sensitive to story length, modulated by agency

Both the *Adventure* and *Romance* stories showed that agency enhanced the neighbor encoding effect. However, this enhancement was not statistically significant in the Adventure story, where Passive participants already exhibited a markedly higher neighbor encoding effect at baseline (*Adventure* > *Romance*: $p = 0.001$). This suggests that story length significantly impacts the neighbor encoding effect. Specifically, the *Adventure* story contained 22–59 events, while the *Romance* story had 128–135 events. The elevated baseline in the Passive condition for the shorter *Adventure* story may indicate a ceiling effect, limiting the potential for agency to further enhance neighbor encoding. In contrast to the *Romance* story, where story-paths were similar in length, the *Adventure* story had considerable variation in path lengths. This allowed us to test how agency's impact on the neighbor encoding effect varies with story length.

First, we selected from all Free and Passive participants in the *Adventure* story those who read shorter *Adventure* stories (~25 events) and those who read longer ones (~50 events, approximately double in length). We then compared the neighbor encoding effect across the two groups (short vs. long) separately for the Passive and the Free condition. Results showed that in the Passive condition, longer stories had a reduced neighbor encoding effect (long mean = 0.201, short mean = 0.330, $p = 0.052$, two-sample t-test). In the Free condition, however, neighbor encoding did not differ significantly between long and short stories (long mean = 0.328, short mean = 0.215, $p = 0.314$, two-sample t-test), supporting that agency can enhance neighbor encoding effect—when given the space to improve. See Supplementary Table S3.

Next, we submitted these participants (N=59) to an ordinary least squares regression to further examine how story length and agency interact to predict individual

neighbor encoding effect. The model included centered story length (to account for the fact that story length cannot be zero), agency condition (Free vs. Passive), and their interaction: *neighbor encoding effect* = $\beta_0 + \beta_1 \cdot (story\ length) + \beta_2 \cdot (agency) + \beta_3 \cdot (story\ length \times agency) + \varepsilon$. Results revealed a significant interaction between story length and agency condition ($p = 0.034$), where longer stories were associated with reduced neighbor encoding effect in the Passive condition (slope = –0.005) but with greater neighbor encoding effect in the Free condition (slope = +0.005). The same result hold true when using all Free and Passive subjects, including ones whose story-path had number of events that fall in between the short and long range (N=71). These results suggest that agency modulates the relationship between story length and neighbor encoding effect. With no agency, longer narrative saw reduced neighbor encoding effect, but agency diminished or even reversed this association.

Overall, these results confirm that agency can enhance neighbor encoding—particularly when the narrative is long enough to allow for such improvements to emerge relative to the Passive condition.

story	cond	subID	Story length (#of event)	Neighbor encoding effect
adventure	free	sub11_1010	22	-0.077
adventure	free	sub3_1002	25	0.01
adventure	free	sub4_1003	25	0.168
adventure	free	sub26_1025	25	0.121
adventure	free	sub6_1005	26	0.516
adventure	free	sub8_1007	27	0.369
adventure	free	sub7_1006	30	0.397
adventure	free	sub14_1013	46	0.387
adventure	free	sub20_1019	46	0.096
adventure	free	sub2_1001	48	0.597
adventure	free	sub19_1018	48	0.378
adventure	free	sub27_1026	49	0.109
adventure	free	sub12_1011	51	0.094
adventure	free	sub28_1027	51	0.29
adventure	free	sub18_1017	52	0.739
adventure	free	sub5_1004	53	0.594
adventure	free	sub23_1022	54	0.194
adventure	free	sub22_1021	59	0.128
adventure	pasv	to11_sub3029	22	0.527
adventure	pasv	to11_sub3037	22	0.599
adventure	pasv	to3_sub3001	25	0.239
adventure	pasv	to26_sub3002	25	0.421
adventure	pasv	to3_sub3028	25	-0.107
adventure	pasv	to4_sub3033	25	0.436

adventure	pasv	to3_sub3044	25	0.378
adventure	pasv	to26_sub3047	25	0.271
adventure	pasv	to4_sub3049	25	-0.051
adventure	pasv	to6_sub3005	26	0.312
adventure	pasv	to6_sub3013	26	0.215
adventure	pasv	to8_sub3025	27	0.297
adventure	pasv	to8_sub3035	27	0.429
adventure	pasv	to7_sub3041	30	0.38
adventure	pasv	to7_sub3045	30	0.6
adventure	pasv	to14_sub3008	46	0.215
adventure	pasv	to20_sub3016	46	0.294
adventure	pasv	to14_sub3018	46	0.153
adventure	pasv	to20_sub3030	46	0.081
adventure	pasv	to14_sub3048	46	0.229
adventure	pasv	to2_sub3027	48	0.508
adventure	pasv	to19_sub3039	48	-0.125
adventure	pasv	to2_sub3042	48	0.215
adventure	pasv	to19_sub3043	48	0.171
adventure	pasv	to27_sub3006	49	-0.157
adventure	pasv	to27_sub3007	49	0.447
adventure	pasv	to27_sub3009	49	0.301
adventure	pasv	to27_sub3014	49	0.248
adventure	pasv	to27_sub3020	49	0.218
adventure	pasv	to28_sub3003	51	0.489
adventure	pasv	to12_sub3004	51	0.097
adventure	pasv	to28_sub3032	51	0.157
adventure	pasv	to12_sub3034	51	0.042
adventure	pasv	to18_sub3019	52	0.434
adventure	pasv	to18_sub3021	52	-0.032
adventure	pasv	to5_sub3011	53	0.524
adventure	pasv	to5_sub3015	53	0.172
adventure	pasv	to23_sub3010	54	0.071
adventure	pasv	to23_sub3024	54	0.135
adventure	pasv	to22_sub3017	59	0.193
adventure	pasv	to22_sub3023	59	0.156

Table S3. Story-paths from Free and Passive participants in the *Adventure* story were divided into short (≤30 events) and long (≥45 events) groups, excluding participants with intermediate story lengths. This resulted in a clear separation in average story length, with short paths averaging 25.7 events and long paths averaging 50.4 events, roughly a twofold difference.

Supplementary References

1. Michelmann, S., Kumar, M., Norman, K. A. & Toneva, M. Large language models can segment narrative events similarly to humans. Preprint at https://doi.org/10.48550/arXiv.2301.10297 (2023).
2. Georgiou, A., Can, T., Katkov, M. & Tsodyks, M. Using large language models to study human memory for meaningful narratives. 2023.11.03.565484 Preprint at https://doi.org/10.1101/2023.11.03.565484 (2023).
3. Bellana, B., Mahabal, A. & Honey, C. J. Narrative thinking lingers in spontaneous thought. *Nat Commun* **13**, 4585 (2022).
4. Engelkamp, J. *Memory for Actions*. vii, 166 (Psychology Press/Taylor & Francis (UK), Hove, England, 1998).
5. Bertsch, S., Pesta, B. J., Wiscott, R. & McDaniel, M. A. The generation effect: A meta-analytic review. *Memory & Cognition* **35**, 201–210 (2007).
6. Tsuji, N. & Imaizumi, S. Sense of agency may not improve recollection and familiarity in recognition memory. *Sci Rep* **12**, 21711 (2022).
7. Lee, H. & Chen, J. Predicting memory from the network structure of naturalistic events. *Nat Commun* **13**, 4235 (2022).
