# Supplementary material for: Interactive narratives reveal the personalizing effect of agency on episodic memory
Source: Nat Commun. 2026 Jun 6;17:7272. doi: 10.1038/s41467-026-73907-2 (PMC13402355; doi:10.1038/s41467-026-73907-2)
Supplement: Supplementary file 2 — Reporting Summary [file 41467_2026_73907_MOESM2_ESM.pdf]

nature portfolio

Corresponding author(s): Xian Li

Last updated by author(s): Mar 22, 2026

Reporting Summary

Nature Portfolio wishes to improve the reproducibility of the work that we publish. This form provides structure for consistency and transparency in reporting. For further information on Nature Portfolio policies, see our Editorial Policies and the Editorial Policy Checklist.

Statistics

For all statistical analyses, confirm that the following items are present in the figure legend, table legend, main text, or Methods section.

n/a	Confirmed	
☐	☒	The exact sample size (*n*) for each experimental group/condition, given as a discrete number and unit of measurement
☐	☒	A statement on whether measurements were taken from distinct samples or whether the same sample was measured repeatedly
☐	☒	The statistical test(s) used AND whether they are one- or two-sided<br>*Only common tests should be described solely by name; describe more complex techniques in the Methods section.*
☒	☐	A description of all covariates tested
☒	☐	A description of any assumptions or corrections, such as tests of normality and adjustment for multiple comparisons
☐	☒	A full description of the statistical parameters including central tendency (e.g. means) or other basic estimates (e.g. regression coefficient) AND variation (e.g. standard deviation) or associated estimates of uncertainty (e.g. confidence intervals)
☐	☒	For null hypothesis testing, the test statistic (e.g. *F*, *t*, *r*) with confidence intervals, effect sizes, degrees of freedom and *P* value noted<br>*Give P values as exact values whenever suitable.*
☒	☐	For Bayesian analysis, information on the choice of priors and Markov chain Monte Carlo settings
☒	☐	For hierarchical and complex designs, identification of the appropriate level for tests and full reporting of outcomes
☐	☒	Estimates of effect sizes (e.g. Cohen's *d*, Pearson's *r*), indicating how they were calculated

Our web collection on statistics for biologists contains articles on many of the points above.

Software and code

Policy information about availability of computer code

Data collection	Participants completed the experiment on the Prolific platform. The experiment presentation code (story delivery, choice interface, and recall collection) was written by the authors and is publicly available on GitHub (see Code Availability).
Data analysis	All data analysis code, including preprocessing, statistical analyses, and figure generation, was written by the authors and is publicly available on GitHub (see Code Availability): https://github.com/xianNeuro/agency-personalizes-episodic-memory.git and linked to Zenodo.

For manuscripts utilizing custom algorithms or software that are central to the research but not yet described in published literature, software must be made available to editors and reviewers. We strongly encourage code deposition in a community repository (e.g. GitHub). See the Nature Portfolio guidelines for submitting code & software for further information.

Data

Policy information about availability of data

All manuscripts must include a data availability statement. This statement should provide the following information, where applicable:

- Accession codes, unique identifiers, or web links for publicly available datasets
- A description of any restrictions on data availability
- For clinical datasets or third party data, please ensure that the statement adheres to our policy

Data are publicly available on GitHub (see Data Availability), including raw behavioral data, derived variables, and all statistical output files used in the analyses.

Research involving human participants, their data, or biological material

Policy information about studies with human participants or human data. See also policy information about sex, gender (identity/presentation), and sexual orientation and race, ethnicity and racism.

Reporting on sex and gender	Sex/gender was determined by participant self-report on Prolific. Gender (male/female) was balanced across experimental conditions using Prolific's recruitment filters. Sex/gender was not a theoretical variable of interest, and no sex- or gender-based analyses were performed. This is justified because the study hypotheses do not concern sex or gender differences.
Reporting on race, ethnicity, or other socially relevant groupings	Race/ethnicity information was collected via participant self-report on Prolific. Race/ethnicity was neither used for quota sampling nor analyzed, as these variables were not relevant to the study hypotheses. Race/ethnicity data are included only in the publicly available dataset for transparency.
Population characteristics	Participants were English-speaking adults recruited through Prolific. No additional covariate-relevant clinical, diagnostic, or genotypic information was collected.
Recruitment	Participants were recruited online via Prolific. Recruitment included filters to balance gender across conditions. No self-selection biases beyond standard online research practices are expected.
Ethics oversight	Informed consent was obtained in accordance with procedures approved by the Johns Hopkins University Institutional Review Board.

Note that full information on the approval of the study protocol must also be provided in the manuscript.

Field-specific reporting

Please select the one below that is the best fit for your research. If you are not sure, read the appropriate sections before making your selection.

☐ Life sciences ☒ Behavioural & social sciences ☐ Ecological, evolutionary & environmental sciences

For a reference copy of the document with all sections, see nature.com/documents/nr-reporting-summary-flat.pdf

Behavioural & social sciences study design

All studies must disclose on these points even when the disclosure is negative.

Study description	This was a quantitative experimental study examining how agency affects memory for naturalistic narratives. Participants read interactive "choose-your-own-adventure" stories with varying levels of agency (Free, Yoked, Passive) and then completed free recall (and recognition, in one story) to assess memory organization.
Research sample	Participants were English-proficient adults recruited via Prolific (N = 324; ages 28 ± 8.8 years; 183 females, 139 males, 2 non-binary). Gender was balanced across conditions using Prolific recruitment filters. Race/ethnicity was collected via self-report but not used for sampling or analysis, as it was not relevant to the study hypotheses. No clinical, diagnostic, or genotypic characteristics were collected.
Sampling strategy	Participants were recruited via Prolific using quota sampling, with quotas applied to balance age and gender. No formal a priori sample-size calculation was performed. Sample sizes were determined based on prior naturalistic narrative-memory studies and practical design constraints, including the requirement to match Yoked and Passive participants to specific Free story-paths so that narrative content was held constant across conditions. These sample sizes were sufficient to detect reliable differences in memory organization across conditions.
Data collection	Participants completed the study online. Stories were presented one sentence at a time in a self-paced reading paradigm, with choices selected using mouse or keyboard inputs. Free recall was typed into a constrained text window that prevented editing of prior sentences, paralleling spoken recall procedures. No researchers were present during data collection, and participants were unaware of the study hypotheses. Causal-relation ratings were obtained separately from trained research assistants using standardized lab protocols.
Timing	Data were collected in single-session experiments for each participant; no multi-day procedures were used. The Adventure story sessions lasted approximately 20–50 minutes, and the Romance story sessions lasted approximately 1.5–2.5 hours.
Data exclusions	No participants were excluded from the main analyses.
Non-participation	No participants dropped out or terminated the study early. All participants who began the session completed it.
Randomization	Participants were randomly assigned to the Free, Yoked, or Passive conditions. For Yoked and Passive participants, assignment to specific story-paths was predetermined to match Free participants' story-paths, ensuring identical stimulus exposure; this non-random allocation was used solely to control for content-related covariates.

Reporting for specific materials, systems and methods

We require information from authors about some types of materials, experimental systems and methods used in many studies. Here, indicate whether each material, system or method listed is relevant to your study. If you are not sure if a list item applies to your research, read the appropriate section before selecting a response.

Materials & experimental systems

n/a	Involved in the study
☒	☐ Antibodies
☒	☐ Eukaryotic cell lines
☒	☐ Palaeontology and archaeology
☒	☐ Animals and other organisms
☒	☐ Clinical data
☒	☐ Dual use research of concern
☒	☐ Plants

Methods

n/a	Involved in the study
☒	☐ ChIP-seq
☒	☐ Flow cytometry
☒	☐ MRI-based neuroimaging

Plants

Seed stocks	Not applicable. No plant materials or seed stocks were used in this study.
Novel plant genotypes	Not applicable. No plant genotypes, genetic modifications, or plant-based experimental procedures were involved in this study.
Authentication	Not applicable. No plant materials or genotypes required authentication, as the study did not involve plants.
