# Supplementary material for: Interactive narratives reveal the personalizing effect of agency on episodic memory
Source: Nat Commun. 2026 Jun 6;17:7272. doi: 10.1038/s41467-026-73907-2 (PMC13402355; doi:10.1038/s41467-026-73907-2)
Supplement: Supplementary file 3 — Transparent Peer Review file [file 41467_2026_73907_MOESM3_ESM.pdf]

nature portfolio

Peer Review File

Interactive narratives reveal the personalizing effect of agency on episodic memory

Corresponding Author: Dr Xian Li

This file contains all reviewer reports in order by version, followed by all author rebuttals in order by version.

Version 1:

Reviewer comments:

Reviewer #1

(Remarks to the Author)

The authors conducted an experiment that consisted of the presentation of two interactive stories, which participants were required to read the story at their own pace. In the Free condition, participants were presented with 2-4 options at specific choice points regarding the next events, thus generating subject-unique story paths. In the Yoked condition, the selections were sometimes ignored (the path was 'yoked' to a Free participant), and in the Passive condition, the participants were not provided the opportunity to choose. After reading the stories, a free memory recall task was administered, and memory was scored for accuracy and semantic and causal relations.
Results
• No significant memory differences were found between groups in recall performance.
• Inter-participant correlations (ISC) were computed for recall vectors (for similar story segments). These correlations were above zero in all conditions (Free, Yoked, Passive), indicating that individuals remembered roughly the same events regardless of conditions.
• The Free condition generated larger variability among participants and therefore lower ISC.
• Semantic centrality – a measure of how strongly inter-connected a given event was with other events in the narrative in terms of meaning.
• Causal centrality – a measure of how strong each event was connected to other events using Causal narrative network analysis (raters).
• Both semantic and causal centrality were correlated with recall performance.
• Semantic influence on memory performance was lower in the Free condition compared to Yoked and Passive.
• The agency condition enhanced the tendency for temporally neighboring events to share the same subsequent memory status (either remembered or forgotten).

The connection between agency and memory formation is a very interesting and appealing topic, and the naturalistic experiment devised by the authors is creative and well thought out. The analyses performed are creative as well, yet I velieve important aspects should be addressed before a clear decision can be made reagrding the interpretations of the results.

Specific comments:
1. Memory performance for the choice events themselves seems to be very low, can the authors explain the reason for this? (Figure 2A).
2. Several analyses use Pearson correlations between binary vectors (0/1), which may not be statistically appropriate. Non-parametric measures such as the Phi coefficient or Chi-squared tests would likely be more suitable for assessing relationships between binary variables.
3. The network visualizations in Figure 3 are intriguing, but I could not find a clear explanation of what the shapes, layout, or edge weights represent, and how these networks were constructed. Please provide more detail on the methodology behind these graphs.
4. There seems to be quite a lot of variability in the experiences of each participant, in terms of story length, number of events, and of course the content of the story lines. Thus, although the analysis was performed on shared sections, the other encoded material may still have influenced the memory performance.
5. There's plenty of reliance on subjective ratings of experimenters, including the process of event segmentation, matching recalled content to story paths, identifying causal relationships between events, etc.
6. Chat-GPT's results in Figure S1 actually look different from the human raters. I didn't quite understand the analysis there.

7. Please indicate the obtained range of semantic centrality values.
8. In general, I find the conclusions not quite strong enough to be stated. The first statement in the discussion is that agency personalized memory in the sense that Free participants were less likely to recall the same shared events as one another. It's still possible that since Free participants experienced different story lines from one another, their memory performance for specific events (the shared events) was less homogeneous than in the other conditions, which were more similar among participants. Thus, it is not necessarily due to the agency component, but rather to the order of events and different interference to memories from other events.
I believe that the appropriate way to examine the data throughout the study would be to correlate performance between pairs of participants with identical storylines, and not between each participant and all the others.
Minor points
Line 307, should be – Figure 3A (not Figure 3A-B top row). Similarly, in line 308, should be – Figure 3B.
Figures 3C & 3D are not referred to in the text.
The supplementary figures are not organized in order, and sometimes the legends have different numbers than the titles.
What qualifications do the 'experienced raters' possess?

(Remarks on code availability)

Reviewer #2

(Remarks to the Author)

This study investigates how agency – specifically, having control over narrative outcomes – modifies episodic memory for complex event sequences. Through interactive "choose-your-own-adventure" stories with varying levels of agency (depending on whether participants were assigned to the Free, Yoked, or Passive conditions), the authors examined how agency influenced recall of narrative events. They found that agency did not merely enhance memory performance, but rather reshaped memory structure to produce more idiosyncratic memory traces.

Overall, this is a very interesting paper, with an interesting study design and a strong analytical approach. It is clearly written and all analyses are well motivated and described. However, given the variety of how agency might influence several other factors along with the subsequent different analyses, the Introduction and Results section feels incredibly dense. I was therefore wondering whether some research questions and their underlying analyses could be moved to the Supplemental Results. In addition to this generic comment, I have a few further major comments, which are summarized below.

1) Throughout the Introduction, the authors assert that past research involving agency has typically considered memory for simplistic stimuli (e.g., item lists). Although this claim is somewhat true, it also overlooks studies that have used virtual environments, object exploration games, and/or spatial mazes to show that active control over movement or exploration enhances spatial memory and the temporal organization of experience in naturalistic contexts [research on active vs. passive spatial exploration may be particularly relevant here; Houser et al., 2022 (which is referenced but not discussed in much detail) may also be useful to expand on in that it used a very similar methodology]. I do agree that this study is unique in the sense that it explores memory for a causally-connected series of events with clear narrative structure; however, I think that the Introduction's current framing makes it sound as though this is the first study to explore the effects of agency in the context of complex, naturalistic stimuli, which isn't quite the case.

2) I found it interesting that the authors included two 'non-agentic' conditions: the Yoked condition, wherein choices were sometimes granted through chance, and the Passive condition, wherein participants had no opportunity to make choices at all. To me, the predictions surrounding these two conditions weren't completely clear, which made it a bit confusing as to why both were included. On the one hand, one might expect to see a clear gradient of agentic effects across the three conditions (full agency in the Free condition; partial agency in the Yoked condition; no agency in the Passive condition). On the other hand, I could also imagine participants in the Yoked condition feeling as though they had no agency (because choices were rarely granted), or possibly even experiencing more frustration and more disengagement than participants in the passive group (meaning that, in cases where agency benefits memory, participants in the Yoked condition might even display worse memory than participants in the Passive condition).

Overall, I think that the introduction would be strengthened by justifying the inclusion of (and potentially making more concrete predictions around) the Yoked and Passive conditions. Relatedly, throughout the Results section, it wasn't clear to me whether there were no differences between the Yoked and Passive conditions, or whether potential differences weren't statistically analysed. I think this could be made more explicit (even just through a blanket statement early in the Results section to say that all three groups were compared, but that only significant differences between groups are listed).

Related to the above points:
a. In the yoked condition, how often were choices granted (by chance) on average? How variable was the range of % choices granted across participants? I could imagine that participants for which choices were granted more often might show memory patterns closer to the 'free' condition whereas participants for which choices were granted only on very few occasions might perform similar to the 'passive' or actually even worse than the 'passive' condition (see my comment above).
b. Therefore, in the Yoked condition, did performance vary as a function of whether or not choices were granted? (e.g., Maybe memories would be stronger and/or more idiosyncratic among participants for whom a higher % of choices were

granted, relative to participants for whom a lower % of choices were granted?)
c. Given that movement through the story was self-paced, were there differences in reading time across the three conditions (and, if so, is it possible that these differences might influence the other between-group differences that were observed?) I wonder whether Yoked and/or Passive group differences might have had less engagement with the narratives overall, such that shorter reading times might mediate the observed relationships between condition and memory.

3) If I understand correctly, in the recall test, participants were explicitly instructed that completeness was more important than accurate sequencing; additionally, they were only able to delete/edit the most recent 10 typed words (lines 615-623). With this structure in mind, I'm not completely convinced that the analyses surrounding temporal contiguity (lines 348 – 360) are valid. It seems that, by explicitly instructing participants not to worry about event sequencing (and by structuring the recall task such that they can't backtrack and insert later-remembered events into earlier sections of their responses), it would be difficult to draw strong conclusions about whether participants are able to successfully recall temporal event order. In this analysis, there were no-between group differences, but I think that this finding is difficult to interpret (and perhaps better omitted) based on how the recall test instructions were delivered. Alternatively, it would be good to assure the reader based on previous literature whether this approach is indeed justified to investigate temporal contiguity.

Minor points:

1) Did participants pick which story (adventure vs. romance) they wanted to read, or were they assigned to one condition?

2) The stories roughly had equal numbers of participants in the yoked and passive condition, but quite an imbalance in the free condition (Adventure: N=22; Romance: N=100). Was there a specific reason for this imbalance? And is it possible to add analyses that show that the findings don't depend heavily on the 'Romance' story and therefore bias the findings.

3) In the section on 'Individual Variability in Recalled Events' (starting on line 205), were ISCs calculated against all participants (regardless of condition), or just all other participants in the same condition?

4) This is a bit pedantic, but I wasn't completely convinced by the examples used to illustrate how agency might activate self-specific knowledge. In particular, the Introduction uses the example of conditioned taste aversion to chocolate ice cream – but, conditioned taste aversions are famously quite strong and automatic, even in cases where participants have no real agency over their choices (e.g., in experiments where rats are administered solutions that will make them sick). I wasn't completely clear on why agency might be expected to influence such a 'fundamental' associative process, and would find it helpful if the authors could highlight specific mechanisms and/or past research that might help strengthen this argument.

(Remarks on code availability)
I did not review the code.

Reviewer #3

(Remarks to the Author)
I co-reviewed this manuscript with one of the reviewers who provided the listed reports. This is part of the Nature Communications initiative to facilitate training in peer review and to provide appropriate recognition for Early Career Researchers who co-review manuscripts.

(Remarks on code availability)
I may be missing it, but was not able to find the codes through this link or on the OSF repository

Reviewer #4

(Remarks to the Author)
The authors investigated how control over an episodic narrative affected different aspects of memory (specifically, for temporal, causal, and semantic relations within the narrative). Participants read stories, with within-story "events" presented sequentially and the degree of control (agency) being manipulated gradually by allowing some participants to make active choices regarding the story progression (that were either always granted or sometimes granted and sometimes not) or not providing participants the chance to make active choices. While agency did not affect overall recall performance or the impact of causal connections between events on memory, it increased interindividual differences in narrative recall, suggesting that agency reorganized memories to more strongly reflect personal experiences.

The manuscript addresses a contemporary topic – the interplay between agency and (episodic) memory – while distinguishing between different memory aspects, which is novel considering previous research. I also think that the agency manipulation is an interesting one that, for example, extends on Ruiz et al. (2023). The presented results are likely interesting and relevant for researchers in the domains of memory, action control, social cognition, and control experience more generally.

Previous literature is referenced appropriately, and the manuscript gives an adequate overview of relevant research. I have suggested some additional literature that may be interesting to consider below.

I have some concerns regarding the reliability and validity of some of the analyses (see below) that should be addressed.

Regarding data & methodology, I have the following concerns and questions:
• Why was there such a big discrepancy in the sample sizes in the Free condition between the Adventure and Romance stories.
• From my understanding, the Adventure story was less well controlled than the Romance story, and the corresponding results do not add much to the manuscript. Consider moving the Adventure story results to the Supplement. This would also make the manuscript more concise.
• The way the different conditions are set up, the predictions or expectations regarding the event following a choice trial were sometimes violated only in the Yoked condition. This factor may affect memory. Based on this, could one infer alternative explanations for the observed data patterns? Please discuss.
• A more serious concern that I have is whether the temporal dependency measure may be confounded with interindividual variations in memory performance. The authors generate a vector of neighbor recall performance, including person- and event-specific average recalls scores for recalling previous and subsequent events, and correlate this vector with the original recall performance vector for each participant. If I have a participant that performs pretty well overall, this participant is likely to have high entries in both the neighbor recall performance and the original recall vector. resulting in a high correlation. However, this is not necessarily indicative of temporal dependency but could simply be attributed to high memory performance more generally (a similar argument can be made for lower levels of performance). The authors should elaborate on this issue (for a discussion on the role of performance when analyzing dependencies for item sets see also Horner & Burgess, 2013 , JEP: General, Schreiner & Meiser, 2023, Behavior Research Methods, and Yu et al. (2025, Memory & Cognition).
• In the paragraph starting on line 186 (section "Agency did not improve recall performance"), it is mentioned that independent raters compared each sentence. I assume this relates to "Matching recall to story-paths" in the Methods section. However, I am missing the information how many raters there were. Also, what was the interrater reliability and how were inconsistencies in ratings resolved? (this is also relevant for other ratings)

The authors report a comprehensive set of analyses that mostly appear to be sound. However, I see the following issues:
• Error bars are not defined (e.g., in Figure 2B, Figure S3).
• Test statistics should be reported for all tests (e.g., in lines 211 and 212 no t values are reported).
• Many analyses rely on Pearson correlations that are subjected to t tests. Correlations tend to be skewed, which is why Fisher's Z transformation is usually applied when averaging correlations. Are the reported results robust when rerunning the analyses based on Z-transformed correlation coefficients?

Inferences and conclusion adequately follow the reported results. I found the Discussion to be comprehensible. However, the authors could extend on the theoretical implications of their findings.

In part, I found the manuscript somewhat difficult to access, particularly the Results section, though I did like the Discussion. Some comments:
• I found the Results section in part hard to follow. I suggest better matching the reporting to the overarching research question and matching reporting to the order of the different subquestions/memory aspects in the Introduction.
• The authors could make a bit clearer which results they initially anticipated and what were more exploratory tests. This is perhaps related to the previous point.
• I believe the paragraph starting on line 99 could be better connected to the previous one.
• I found the paragraph starting on line 128, for the preliminary results summary at the end of the introduction, hard to understand without first reading the Results section. Could this paragraph be made more comprehensible without having to refer to the results section?

(Further) comments to the authors:
• The relationship between events and event sequences or episodic narratives could be spelled out a bit more clearly. In this context, the framework by Andermane et al. (2021, Current Opinion in Neurobiology) may be useful to consider.
• (Minor) It may be interesting to connect the present research to the enactment effect and to the generation effect.
• (Minor) Note that, for Ref. 8 (Hon & Yeo, 2021), participants could not "choose" the identity of items they produced, but what was manipulated was the compatibility of a spatial effect dimension to a participant's action, which was then followed by the item identity, and items in compatible action-effect episodes were remembered better than items in incompatible ones (see also Schreiner et al., 2025, Memory & Cognition, but see Tsuji & Imaizumi, 2022, Scientific Reports). One may refer to this as control, but I think "choosing" the item identity does not quite capture it.
• (Minor) In the context of the finding that agency did not affect recall performance it may also be interesting to look at findings by Tsuji and Imaizumi (2022, Scientific Reports, and Schreiner et al., 2024, Quarterly Journal of Experimental Psychology). I saw that the findings by Tsuji and Imaizumi were mentioned in the Supplement though.
• Please explain the different colors in Figure S7 in the figure note.
• (Minor) Line 406 is missing an "of" before "causal relations".

(Remarks on code availability)
The linked repository contains python code to reproduce the figures. However, please double-check that the file paths match the structure of the repository, for example, in "1_plot_bars.ipynb" I had to remove "data" from file_path =

'./1_bar_plots/data/bar_data.xlsx'. This would increase usability.

I only found code to reproduce the figures but not code for the different statistical tests. Is this code available somewhere?

Version 2:

Reviewer comments:

Reviewer #1

(Remarks to the Author)
I appreciate the authors' thorough and thoughtful responses to the comments raised in my original review. Here are remaining issues with the authors' responses:

Comment1:
In the original review, I wrote that "Memory performance for the choice events themselves seems to be very low, can the authors explain the reason for this? (Figure 2A)."
The authors replied -
We thank the reviewer for highlighting this important detail. The choice events in our paradigm differ in nature from the other (non-choice) narrative events—they describe specific actions that participants can select and thus are much shorter (around 5 words) than non-choice events (~40–50 words). As the reviewer noticed, this brevity makes them less likely to be recalled in absolute terms. However, when recall is normalized by the number of words in each event, we see the opposite pattern: participants recall choice events more often per word than normal story events (see our new analysis in the figure below). This suggests that, although brief, choice events are encoded relatively well, consistent with the idea that making a choice improves memory.
We have added this new figure to the Supplement in section S4, Figure S3: "Memory for choice and non-choice events controlling for word count".

I appreciate the authors' response and the additional analysis; however, I am not fully convinced that it directly addresses the concern raised in my original comment. As described in the text, the stories were segmented into events, and participants' recall responses were subsequently assigned to these events. In the revised analysis, the authors appear to assume a direct relationship between the number of words in the original narrative and the likelihood of later recall, normalizing recall (binary) by word count. However, text length may not be a primary determinant of subsequent memory performance. Rather, I would expect that words associated with the opportunity to make a choice would be better remembered than words from non-choice periods, largely independent of text length. Demonstrating such an effect would more directly support the interpretation that agency enhances memory, which was the central issue raised in my original comment.

Comment3:
The network visualizations in Figure 3 are intriguing, but I could not find a clear explanation of what the shapes, layout, or edge weights represent, and how these networks were constructed. Please provide more detail on the methodology behind these graphs.

We appreciate this helpful request for clarification. The networks in Figure 3 are constructed from event-by-event relation matrices (semantic similarity or causal connection strength). Each node corresponds to a story event. Edges connect pairs of events if their similarity exceeds a fixed threshold of $\tau = 0.1$, with the edge thickness and color intensity proportional to the similarity weight. Node size is scaled by weighted degree centrality (sum of connected edge weights). Node positions are determined by a force-directed layout (Fruchterman–Reingold in MATLAB), which places more strongly connected events closer together.

I thank the authors for their clarifications. However, I remain unclear about several aspects of the network visualizations. First, do the spatial positions of the nodes carry specific meaning—that is, does the area where the networks are placed correspond to particular events? Second, what do the node colors (orange and blue) represent?
In addition, what do the color scales in the causal network matrices indicate (Figure 3B)? Please add a color mapping.

Line 780 – Figure 2B should be Figure 2A.

(Remarks on code availability)

Reviewer #2

(Remarks to the Author)
All previously raised concerns have been appropriately addressed in the revisions.

(Remarks on code availability)

Reviewer #3

(Remarks to the Author)
I co-reviewed this manuscript with one of the reviewers who provided the listed reports. This is part of the Nature Communications initiative to facilitate training in peer review and to provide appropriate recognition for Early Career Researchers who co-review manuscripts.

(Remarks on code availability)

Reviewer #4

(Remarks to the Author)
I am satisfied with the authors' responses to my previous comments and appreciate the level of detail of the responses. I believe the manuscript has become much clearer and better streamlined. I have no further comments.

(Remarks on code availability)
I was able to run all_analyses.py and could reproduce the complete report (after providing my local path in base_path). I also randomly checked some of the individual run files and was able to create the corresponding output.

Version 3:

Reviewer comments:

Reviewer #1

(Remarks to the Author)
I appreciate the authors' responses, which answered the topics I raised in a satisfactory manner.

(Remarks on code availability)

Open Access This Peer Review File is licensed under a Creative Commons Attribution 4.0 International License, which permits use, sharing, adaptation, distribution and reproduction in any medium or format, as long as you give appropriate credit to the original author(s) and the source, provide a link to the Creative Commons license, and indicate if changes were made.
In cases where reviewers are anonymous, credit should be given to 'Anonymous Referee' and the source.
The images or other third party material in this Peer Review File are included in the article’s Creative Commons license, unless indicated otherwise in a credit line to the material. If material is not included in the article’s Creative Commons

license and your intended use is not permitted by statutory regulation or exceeds the permitted use, you will need to obtain permission directly from the copyright holder.
To view a copy of this license, visit https://creativecommons.org/licenses/by/4.0/

We greatly appreciate the reviewers' thoughtful and constructive comments. Their insights substantially strengthened the clarity, theoretical framing, methodological transparency, and overall rigor of the manuscript. We have carefully considered each of the reviewers' points, and all original and newly added analyses converge on the same core conclusions. We hope that the revised manuscript and accompanying materials are now suitable for publication.

Throughout the response letter, we use consistent color-coding and formatting to indicate added text, revisions made in response to reviewer comments, and changes made for clarity: black fonts are the verbatim comments from reviewers, blue fonts are our responses, *italicized black fonts* are quotes of our documents from the original submission (manuscript, supplement), and *italicized red fonts* are used to mark new additions.

Reviewer #1 (Remarks to the Author):

The authors conducted an experiment that consisted of the presentation of two interactive stories, in which participants were required to read the story at their own pace. In the Free condition, participants were presented with 2-4 options at specific choice points regarding the next events, thus generating subject-unique story paths. In the Yoked condition, the selections were sometimes ignored (the path was 'yoked' to a Free participant), and in the Passive condition, the participants were not provided the opportunity to choose. After reading the stories, a free memory recall task was administered, and memory was scored for accuracy and semantic and causal relations.

Results

• No significant memory differences were found between groups in recall performance.
• Inter-participant correlations (ISC) were computed for recall vectors (for similar story segments). These correlations were above zero in all conditions (Free, Yoked, Passive), indicating that individuals remembered roughly the same events regardless of conditions.
• The Free condition generated larger variability among participants and therefore lower ISC.
• Semantic centrality – a measure of how strongly inter-connected a given event was with other events in the narrative in terms of meaning.
• Causal centrality – a measure of how strong each event was connected to other events using Causal narrative network analysis (raters).
• Both semantic and causal centrality were correlated with recall performance.
• Semantic influence on memory performance was lower in the Free condition compared to Yoked and Passive.
• The agency condition enhanced the tendency for temporally neighboring events to share the same subsequent memory status (either remembered or forgotten).

The connection between agency and memory formation is a very interesting and appealing topic, and the naturalistic experiment devised by the authors is creative and well thought out. The analyses performed are creative as well, yet I believe important aspects should be addressed before a clear decision can be made regarding the interpretations of the results.

> We thank the reviewer for their positive assessment of the topic, creativity, and planning of our study.

Specific comments:
1. Memory performance for the choice events themselves seems to be very low, can the authors explain the reason for this? (Figure 2A).

We thank the reviewer for highlighting this important detail. The choice events in our paradigm differ in nature from the other (non-choice) narrative events—they describe specific actions that participants can select and thus are much shorter (around 5 words) than non-choice events (~40–50 words). As the reviewer noticed, this brevity makes them less likely to be recalled in absolute terms. However, when recall is normalized by the number of words in each event, we see the opposite pattern: participants recall choice events *more often per word* than normal story events (see our new analysis in the figure below). This suggests that, although brief, choice events are encoded relatively well, consistent with the idea that making a choice improves memory.

We have added this new figure to the Supplement in section S4, Figure S3: "Memory for choice and non-choice events controlling for word count".

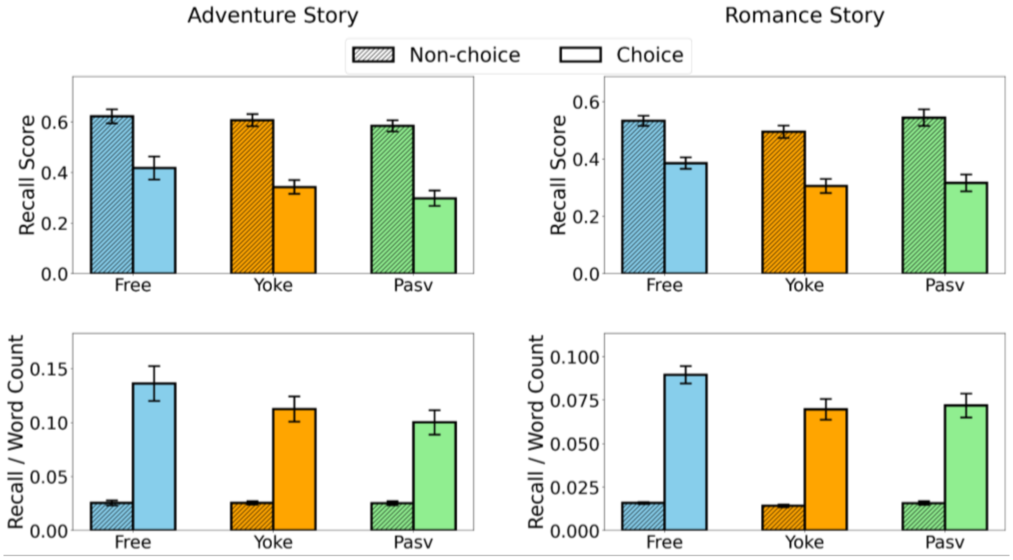

Supplementary Figure S3| Recall and recall per word split by choice and non-choice event. *The figure displays two measures organized in rows: Recall Score (top row) and Recall per Word (bottom row). For each measure, the left column shows data from the Adventure story, and the right column shows data from the Romance story. Each panel shows six bars representing the three agency conditions (Free: sky blue, Yoked: orange, Passive: light green) × two event types (Non-choice: hatched bars with diagonal slashes, Choice: solid bars). Error bars represent standard error of the mean across subjects. The figure demonstrates that choice events showed higher recall per word than non-choice events across all conditions and both stories, with agency condition differentially affecting memory for choice versus non-choice events in the Romance story.*

Importantly, while choice events may differ from non-choice events, this does not drive our main findings. Our main results on the agency's idiosyncratic effects were conducted both including and excluding these choice events to make sure that the memory effects we saw were not driven by choice events alone. This point was addressed in the analysis accompanying Figure 2B-C where we repeated idiosyncrasy analyses both including and excluding choice events (page 7-8 lines 224-235 in the revised manuscript):

"In the Yoked and Passive conditions, multiple participants followed the story-path corresponding to each of the 18 unique Free participant story-paths. To ensure that the above-reported higher inter-participant memory similarity in the Yoked and Passive

conditions was not due to participants sharing the same story-path, we conducted non-parametric tests of Recall ISC (Figure 2B-C). We randomly sampled one Yoked and one Passive participant from each of the 18 story-paths to form a sample of 18 Yoked and 18 Passive participants; thus, no pairs within these samples read the same story-path. This process was repeated 10,000 times to generate distributions of Recall ISC for both the Yoked and Passive conditions. The analysis confirmed that the Free condition's Recall ISC was significantly lower than that of the Passive ($p < 0.001$; excluding choice events, $p < 0.001$) and the Yoked ($p < 0.001$; excluding choice events, $p = 0.006$) conditions (Figure 2B-C, right)."

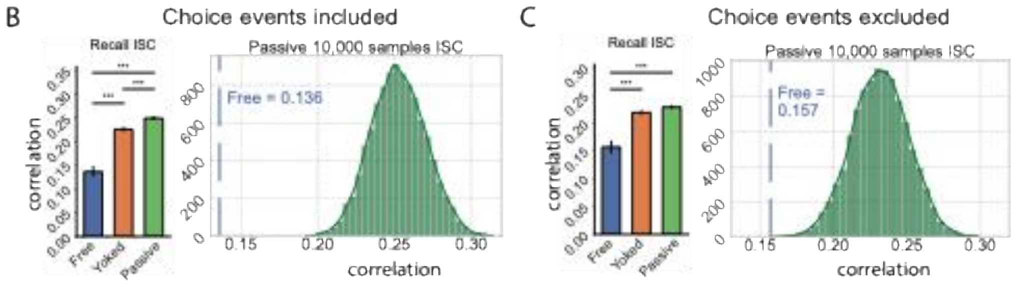

2. Several analyses use Pearson correlations between binary vectors (0/1), which may not be statistically appropriate. Non-parametric measures such as the Phi coefficient or Chi-squared tests would likely be more suitable for assessing relationships between binary variables.

We thank the reviewer for raising an important statistical point. Our goal in these analyses was to capture the *similarity in which events are recalled across subjects*—that is, whether two participants tended to recall the same events, not simply whether their overall recall rates were different. For this purpose, Pearson correlation is a standard similarity metric that can be applied to binary vectors.

For two binary vectors, Pearson's r is mathematically identical to the Phi coefficient. Suppose we compare two participants' recall of the same story, event by event. Each participant's recall can be represented as a binary vector X, Y ∈ {0,1}, where 1 = event recalled and 0 = event not recalled. Their joint recall patterns can be summarized in a 2×2 table:

	Participant B recalled (1)	Participant B not recalled (0)
Participant A recalled (1)	n11: both recalled	n10: recalled by A only
Participant A not recalled (0)	n01: recalled by B only	n00: not recalled by either

Variable definitions and mathematical deductions:

- n_{11}: number of events recalled by both participants
- n_{10}: number of events recalled only by participant A
- n_{01}: number of events recalled only by participant B
- n_{00}: number of events not recalled by either participant
- Total number of events: $N = n_{11} + n_{10} + n_{01} + n_{00}$

Step 1. Pearson correlation definition:

$$r_{XY} = \frac{\mathrm{Cov}\ (X, Y)}{\sigma_X \sigma_Y}$$

Step 2. Expand covariance for binary data:

$$\mathrm{Cov}\,(X, Y) = \frac{n_{11}n_{00} - n_{10}n_{01}}{N^2}$$

Step 3. Standard deviations of binary variables:

$$\sigma_X = \sqrt{\frac{n_{1\cdot}}{N} \cdot \frac{n_{0\cdot}}{N}}, \sigma_Y = \sqrt{\frac{n_{\cdot 1}}{N} \cdot \frac{n_{\cdot 0}}{N}}$$

Step 4. Substitute into Pearson formula:

$$r_{XY} = \frac{n_{11}n_{00} - n_{10}n_{01}}{\sqrt{n_{1\cdot}\, n_{0\cdot}\, n_{\cdot 1}\, n_{\cdot 0}}}$$

Step 5. Compare with Phi coefficient:

$$\Phi = \frac{n_{11}n_{00} - n_{10}n_{01}}{\sqrt{n_{1\cdot}\, n_{0\cdot}\, n_{\cdot 1}\, n_{\cdot 0}}}$$

Thus, Pearson correlation is mathematically equivalent to Phi coefficient for binary data ($r_{XY} = \Phi$).

In contrast, the Chi-squared test addresses a different question—testing independence at the group level—rather than measuring pairwise similarity of recall patterns. Alternatively, one could also measure raw overlap (e.g., proportion of events both recalled by a pair of subjects). However, this measure does not normalize for base-rate differences. Pearson, or Φ, provides a normalized measure more statistically suited for our goal.

Overall, for the purpose of comparing inter-subj recalled events similarity, we feel that Pearson correlation between two binary vectors best captures our variances of interest. We have added the following explanatory text to Methods:

> *"While Phi coefficient or Chi-squared tests are often used for assessing relationships between binary variables, here we chose to use Pearson correlation because for two binary vectors, the Pearson correlation coefficient is mathematically identical to the Phi coefficient. We chose not to use a Chi-squared test as it tests independence at the group level rather than measuring pairwise similarity of recall patterns."*

3. The network visualizations in Figure 3 are intriguing, but I could not find a clear explanation of what the shapes, layout, or edge weights represent, and how these networks were constructed. Please provide more detail on the methodology behind these graphs.

We appreciate this helpful request for clarification. The networks in Figure 3 are constructed from event-by-event relation matrices (semantic similarity or causal connection strength). Each node corresponds to a story event. Edges connect pairs of events if their similarity exceeds a fixed threshold of $\tau = 0.1$, with the edge thickness and color intensity proportional to the similarity weight. Node size is scaled by weighted degree centrality (sum of connected edge weights). Node positions are determined by a force-directed layout (Fruchterman–Reingold in MATLAB), which places more strongly connected events closer together.

These visualizations are therefore intended to provide an intuitive overview: clusters of strongly related events appear spatially close, node size highlights central events, and edge weights convey the strength of pairwise similarity. We have added the following explanations to the paper in Methods and the legend for Figure 3:

> *"Network visualization (Figure 3A-B) in matrix format showed the event-by-event pairwise Pearson similarity; those in the graph format (top right corners) are constructed from the*

similarity matrices: each node corresponds to a story event; edges connect pairs of events if their similarity exceeds a fixed threshold of τ = 0.1, with the edge thickness and color intensity proportional to the similarity weight; node size is scaled by weighted degree centrality (sum of connected edge weights); node positions are determined by a force-directed layout (Fruchterman–Reingold in MATLAB), which places more strongly connected events closer together."

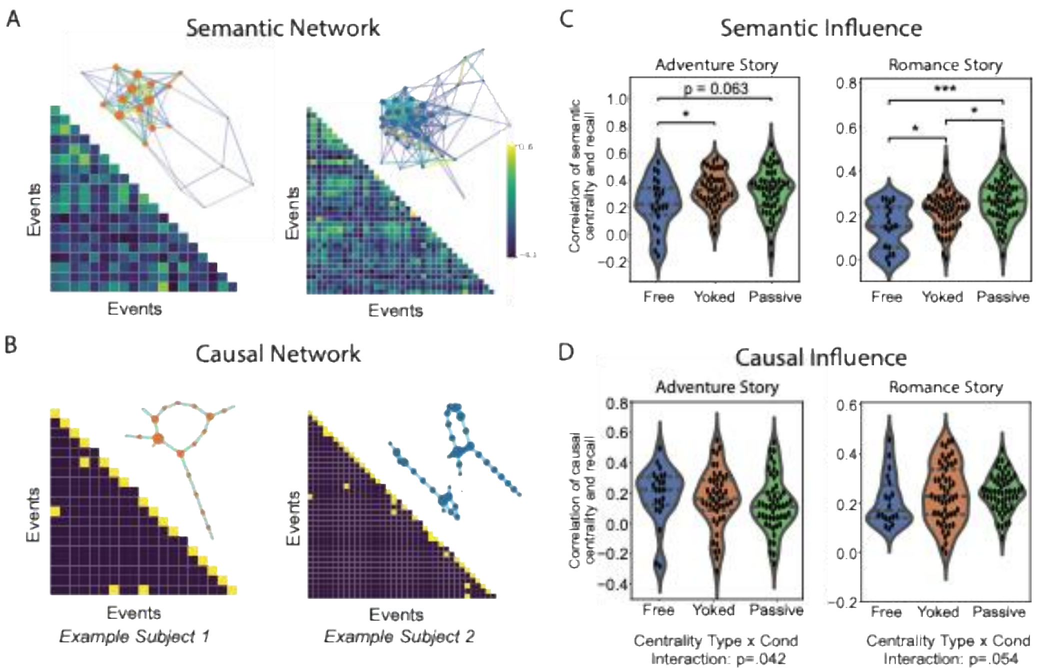

Fig. 3| Semantic and causal centrality effects on memory. A, Semantic similarity matrix showing the USE cosine similarity between pairs of events for example Adventure (left) and Romance (right) stories, and the corresponding semantic network plots for the same stories. Each node in the network graphs (top right corners) represents an event; edges (τ > 0.1) scale in thickness and color by similarity; node size reflects degree centrality. B, Causal matrix showing the human-judged causal connection between pairs of events for example Adventure (left) and Romance (right) stories, and the corresponding causal network plots for the same stories (network graphs constructed using the same methods as in A). C, Distributions of semantic influence, i.e., the correlation between semantic centrality and recall performance, in the Adventure story (left) and the Romance story (right) in each condition. Each dot represents one participant. D, Distributions of causal influence, i.e., the correlation between causal centrality and recall performance, in the Adventure story (left) and the Romance story (right) in each condition. Each dot represents one participant.

4. There seems to be quite a lot of variability in the experiences of each participant, in terms of story length, number of events, and of course the content of the story lines. Thus, although the analysis was performed on shared sections, the other encoded material may still have influenced the memory performance.

We thank the reviewer for this thoughtful point and would like to clarify the design. For every Free story-path, there were at least two Yoked and two Passive participants who experienced identical story text, word for word – not only in the shared sections but across the entire story. This ensures that the narrative context (the "other encoded material") outside of the shared sections was exactly the same across conditions. To further verify that results were not driven by story-path differences, we performed permutation tests in which we drew samples of Passive participants whose story paths were exact 1-to-1 matches with those in the Free condition (as

discussed in our response above to your question 1, referring to Fig. 2B-C; also pasted below for your convenience). These analyses confirmed the increased memory idiosyncrasy in the Free condition, ruling out variability in surrounding material as an explanation.

Page 7-8 lines 224-235 in the revised manuscript:

> *"In the Yoked and Passive conditions, multiple participants followed the story-path corresponding to each of the 18 unique Free participant story-paths. To ensure that the above-reported higher inter-participant memory similarity in the Yoked and Passive conditions was not due to participants sharing the same story-path, we conducted non-parametric tests of Recall ISC (Figure 2B-C). We randomly sampled one Yoked and one Passive participant from each of the 18 story-paths to form a sample of 18 Yoked and 18 Passive participants; thus, no pairs within these samples read the same story-path. This process was repeated 10,000 times to generate distributions of Recall ISC for both the Yoked and Passive conditions. The analysis confirmed that the Free condition's Recall ISC was significantly lower than that of the Passive ($p < 0.001$; excluding choice events, $p < 0.001$) and the Yoked ($p < 0.001$; excluding choice events, $p = 0.006$) conditions (Figure 2B-C, right)."*

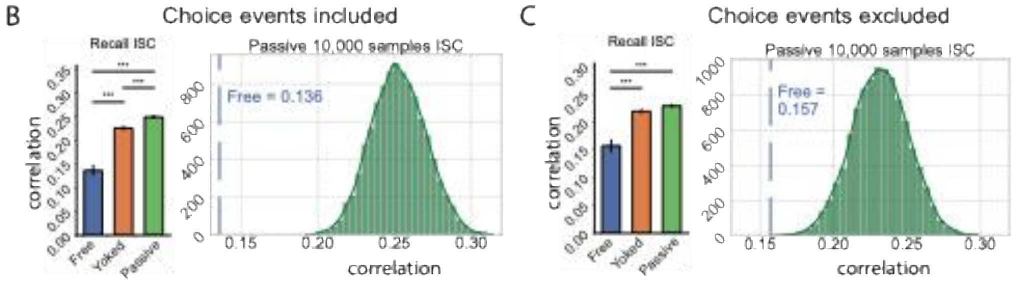

In order to clarify this issue for readers, we have added the following text to Results:

> *"Overall, this design allowed participants to explore a naturalistic choose-your-own-adventure narrative with varying degrees of agency. Critically, variability in story paths was fully matched across conditions: each unique path generated by a Free participant was read verbatim by multiple Yoked and Passive participants, including the same physical action of advancing screens (e.g., mouse clicks). Thus, potential differences in memory across conditions cannot be attributed to any stimulus variation other than the degree of perceived agency."*

5. There's plenty of reliance on subjective ratings of experimenters, including the process of event segmentation, matching recalled content to story paths, identifying causal relationships between events, etc.

We thank the reviewer for highlighting this important issue. While these annotation steps inevitably involve human raters, we took several measures to maximize objectivity and reliability.

Regarding event segmentation: For both the Adventure and Romance stories event boundaries were determined via discussion among the experimenters and story authors until agreement was achieved. These delineations were finalized before any memory data were collected.

Regarding matching recalled content to story paths: Across the two stories, we collected recall from a total of 324 participants (Adventure: N = 116; Romance: N = 208), spanning 22 Adventure versions and 100 Romance versions. Recall matching was performed by the team of trained raters. Raters first read the story in full, then read each recall file, and matched recall segments to the corresponding story events. This was a time-intensive process: rating one Adventure recall typically took 30–60 minutes, while rating a Romance recall took 60–180 minutes, amounting to roughly 500 hours of coding in total.

Our laboratory maintains a team of 5-8 raters (undergraduate research assistants) who perform tasks such as this routinely, as we have similar needs across many of our experiments (speech transcription, stimulus labeling, matching, etc.). The raters are trained by a graduate-student team leader who also regularly reviews the raters' work for quality and provides feedback.

To maximize consistency, each story-path's recall files (Free, Yoked, Passive) were assigned to the same rater for the Romance dataset, ensuring that cross-condition comparisons were not confounded by rater differences. Raters were blind to experimental conditions, and their only task was to align recall segments with story events.

The task of matching depends largely on objective features (e.g., did the participant mention that the protagonist went to a coffee shop, yes or no?) and the type of hand-coding described above is currently the standard approach in the literature for recall-to-story content matching and event segmentation (Baldassano et al., 2017; Chen et al., 2017; Flores et al., 2017; Furman et al., 2007; Gold et al., 2017; Zadbood et al., 2017). In fact, this type of labeling is considered objective enough that it is tractable for automation via LLMs; publications in just the last 2 years have started to demonstrate that LLMs can perform the task with comparable-to-human performance, e.g., recall-to-story content matching (Chen & Bornstein, 2024; Georgiou et al., 2025; Panela et al., 2025), and event segmentation (Michelmann et al., 2025). As the field moves toward adopting such automated annotation pipelines in replacement of human judgments, the high-quality human-rated data from the current study will serve as a valuable resource for training and validating these future systems.

Regarding causal relationship judgments: Each pair of events was rated by multiple raters for the Romance story and analyses were performed on the average across raters, reducing idiosyncratic bias. This procedure was adopted from prior papers (Antony et al., 2024; Lee & Chen, 2022).

We agree with the reviewer that subjectivity of ratings is an important consideration in this field, and we have added a paragraph to the Discussion in which explicitly acknowledges the current reliance on human coding in narrative memory research. We also highlight the exciting potential of large language models to provide scalable, automated annotation in future work, while noting that questions of objectivity and bias will remain important to evaluate:

> *"A necessary aspect of research using naturalistic stimuli such as narratives is that labeling of many stimulus and behavioral response features relies on human judgments: e.g., in the current study, delineation of event boundaries, causal links between events, segmentation of participants' recall and assignment of those segments to the story events. For each such variable, researchers must choose between 1) having the features "curated" by the researchers themselves or by trained labelers, 2) estimating population-level human judgments by running a separate experiment (commonly performed for*

event boundary selection), or 3) some combination. The "curation" approach avoids some of the messiness which comes with human data, while the "separate experiment" approach may allow for scientific claims arising from the human judgment data. In our study, we largely took the curation approach (see Methods). Interestingly, recent publications have demonstrated that LLMs can perform some of these judgment tasks with comparable-to-human performance (e.g., recall-to-story content matching (Georgiou et al., 2025; Raccah et al., 2024) and event segmentation (Michelmann et al., 2025; Panela et al., 2025)). As the field moves toward adopting such automated annotation pipelines, the high-quality human-rated data from the current study will serve as a valuable resource for training and validating these future systems. However, it is also important to note that the judgments of LLMs may not be more "objective" than those of humans; certain judgments have no correct answer, but rather should be expected to generate a distribution of responses from humans. It would be a mistake to replace human ratings with automated tools if doing so creates an illusion that the cognition underlying those judgments is well-understood (Crockett & Messeri, 2023)."

We have also added the following text to the Methods, prior to the subsection *Event Segmentation*:

"Human ratings for naturalistic stimuli

Our laboratory maintains a team of raters (research assistants) who routinely perform tasks pertaining to processing and ratings of naturalistic stimuli, as we have similar needs across many of our experiments (speech transcription, stimulus labeling, matching, etc.). The raters are trained by a graduate-student team leader who also regularly reviews the raters' work for quality and provides feedback. They followed a standardized written instruction set (publicly available in the "instruct-raters" folder on GitHub; see Data Availability), ensuring consistency across coders."

For more detailed information about the instructions given to raters, please see the instruction sheets included in the "instruct-rater" folder under our Github repository in our original and current submission (https://github.com/xianNeuro/agency-personalizes-episodic-memory).

6. Chat-GPT's results in Figure S1 actually look different from the human raters. I didn't quite understand the analysis there.

We appreciate that the reviewer carefully went through the Supplementary Materials, and we are happy to clarify Figure S1. In this figure, the blue curve reflects agreement between pairs of human raters, the red curve reflects agreement between GPT runs with itself, and the green curve reflects agreement between human raters and GPT. The key result is that the green and blue curves overlap closely, showing that GPT's ratings are as similar to human ratings as human ratings are to each other. The red curve is higher because GPT is internally more consistent than two independent humans, which is expected. Importantly, consistency across trials of queries with a limited variation is exactly what we want in such research studies to serve as a tool that captures a generic casual relationship between events in a narrative. Overall, GPT agrees with itself more than two human raters agree with one another, but when compared with humans, GPT falls within the same range of reliability as human–human agreement.

We have revised Figure S1 and its legend in order to provide a more thorough description of what it depicts:

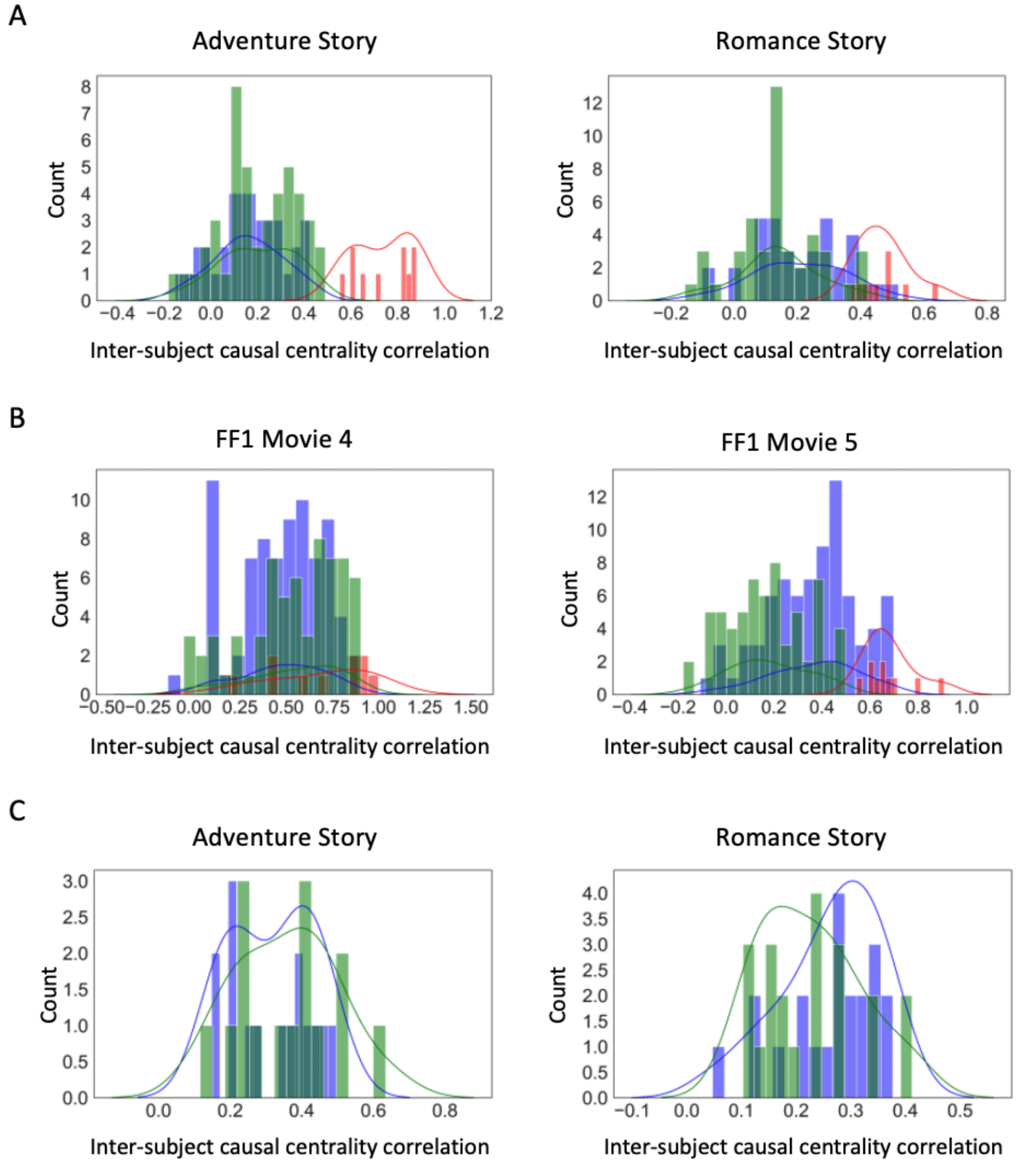

Supplementary Fig. S1| GPT-4 agent performance on identifying causally related events in narratives compared to human raters. A, *Left: The similarity of causal centrality vectors among human raters (blue, n=9), among iterations of GPT4 agent's responses (red, n=5), and between the human raters and the GPT4 agent iterations (green) in one Adventure story path (story1020). The blue curve reflects inter-human reliability, the red curve reflects GPT-4's internal consistency, and the green curve reflects human–GPT-4 similarity. Overlap between the blue and green distributions indicates that GPT-4's causal-centrality estimates are as similar to human raters as human raters are to each other. Right: Same as Left for one Romance story path (story4003).* ***B,*** *The same three distributions were plotted for a short movie narrative (FF1 movie 4; left) and a long movie narrative (FF1 movie 5; right; Humans n=13, GPT iterations = 5). GPT-4's causal-centrality estimates closely matched human ratings for the short movie and fell within the human-human reliability range for the long movie.* ***C,*** *The two distributions in the plot (left) compares the predictability on 15 participants' event memory across 3 Adventure story paths using the causal centrality derived from human raters and that derived from GPT4 agent. Similarly, the two distributions in the plot (right) compare the predictability of 21 participants' event memory across three Romance story paths. In both cases, GPT-4–derived causal-centrality predicted memory performance comparably to human-derived measures.*

7. Please indicate the obtained range of semantic centrality values.

Semantic centrality values generally ranged from 0.02–0.25 for the Adventure story and from 0–0.30 for the Romance story. To illustrate this, we have plotted the range of semantic centrality values across events for each Free subject below. Importantly, the semantic centrality score is determined solely by the story text itself (see Methods lines 804-811) and thus is identical across the Free, Yoked, and Passive conditions.

This figure has been added to the paper as Supplementary Figure S6:

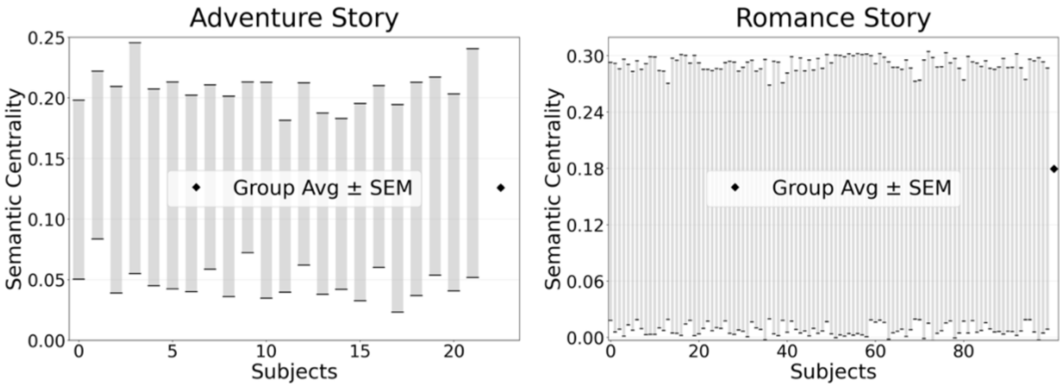

Supplementary Figure S6| Range of semantic centrality values per subject. *The figure displays the range of semantic centrality values for individual subjects in both the Adventure Story (left panel, n=22) and Romance Story (right panel, n=100). Each vertical light gray bar represents one subject, with black horizontal lines capping the minimum and maximum semantic centrality values for that subject across all events in their story path. The black diamond marker with error bars positioned to the right of the individual subject data shows the group average ± standard error of the mean (SEM) across all subjects and events for each story (Adventure: mean = 0.126, SEM = 0.001; Romance: mean = 0.180, SEM = 0.001). This visualization demonstrates the variability in semantic centrality values both within individual subjects (range) and across the group (group average with SEM) for both narrative contexts.*

8. In general, I find the conclusions not quite strong enough to be stated. The first statement in the discussion is that agency personalized memory in the sense that Free participants were less likely to recall the same shared events as one another. It's still possible that since Free participants experienced different story lines from one another, their memory performance for specific events (the shared events) was less homogeneous than in the other conditions, which were more similar among participants. Thus, it is not necessarily due to the agency component, but rather to the order of events and different interference to memories from other events.
I believe that the appropriate way to examine the data throughout the study would be to correlate performance between pairs of participants with identical storylines, and not between each participant and all the others.

The reviewer raised the possibility that the variability of different story-paths (different contexts for the shared story sections) across Free subjects could have resulted in their less homogeneous memory. This is an important point, and we believe it arises from a miscommunication of the design, so we are grateful for the opportunity to clarify. Our design fully controls for variability in story paths across conditions: for every Free story-path, at least two Yoked and two Passive participants experienced the identical text word by word, ensuring that the shared events were always embedded in the same narrative context across groups. In other words, the Free

participants experienced different story lines from one another, and the Yoked and Passive participants also experienced different story lines from one another to the exact same extent.

This specific point was addressed by the analysis in which we performed permutation tests that drew equal-sized samples of Yoked and Passive participants whose story paths were exact 1-to-1 matches with those in the Free condition (see lines 228–239 in the original submission and Fig. 2B–C). These analyses confirmed the increased memory idiosyncrasy in the Free condition, indicating that the effect is attributable to agency rather than variability in event order or interference from differing contexts. We pasted the analysis and results below for your convenience (page 7-8 lines 224-235 in the revised manuscript):

> *"In the Yoked and Passive conditions, multiple participants followed the story-path corresponding to each of the 18 unique Free participant story-paths. To ensure that the above-reported higher inter-participant memory similarity in the Yoked and Passive conditions was not due to participants sharing the same story-path, we conducted non-parametric tests of Recall ISC (Figure 2B-C). We randomly sampled one Yoked and one Passive participant from each of the 18 story-paths to form a sample of 18 Yoked and 18 Passive participants; thus, no pairs within these samples read the same story-path. This process was repeated 10,000 times to generate distributions of Recall ISC for both the Yoked and Passive conditions. The analysis confirmed that the Free condition's Recall ISC was significantly lower than that of the Passive ($p < 0.001$; excluding choice events, $p < 0.001$) and the Yoked ($p < 0.001$; excluding choice events, $p = 0.006$) conditions (Figure 2B-C, right)."*

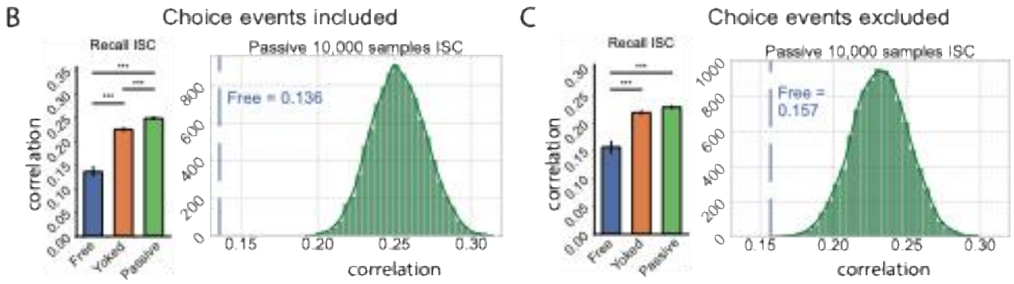

Therefore, the increased idiosyncrasy in the Free condition (as compared to the Yoked and Passive condition) cannot be attributed to context differences across Free subjects, because the same story contexts were experienced by Yoked and Passive subjects. In order to clarify this issue for readers, we have added the following text to Results:

> *"Overall, this design allowed participants to explore a naturalistic choose-your-own-adventure narrative with varying degrees of agency. Critically, variability in story paths was fully matched across conditions: each unique path generated by a Free participant was read verbatim by multiple Yoked and Passive participants, including the same physical action of advancing screens (e.g., mouse clicks). Thus, potential differences in memory across conditions cannot be attributed to any stimulus variation other than the degree of perceived agency."*

We also appreciate the reviewer's suggestion to restrict comparisons to pairs with identical storylines. In our design, however, no two Free participants share identical story paths (because there were so many choices that it never happened by chance), so an "identical-storyline only"

analysis cannot be conducted for Free-Free subject pairs. While it is possible to pair a Free participant with their context-matched Passive counterparts (we have ≥2 Passive per Free path), a Free-Passive analysis would re-use the same Passive participants across multiple pairs, introducing non-independence and unequal weighting that confounds inference. The most statistically proper and dependency-free comparison is therefore Free-Free vs. Passive-Passive, with context matched 1-on-1 across the two conditions; this is the analysis we report in the results as quoted above. This permutation sampling approach directly addresses the reviewer's intent—testing similarity when story context is held constant—while preserving independence of observations.

Minor points
Line 307, should be – Figure 3A (not Figure 3A-B top row). Similarly, in line 308, should be – Figure 3B. Figures 3C & 3D are not referred to in the text.

Thank you for pointing these out! We have corrected these texts.

The supplementary figures are not organized in order, and sometimes the legends have different numbers than the titles.

Thank you for noting this. We have now ordered the supplementary figures and tables sequentially, following the journal guideline.

What qualifications do the 'experienced raters' possess?

Our laboratory maintains a team of 5-8 raters (undergraduate research assistants) who perform tasks such as this routinely, as we have similar needs across many of our experiments (speech transcription, stimulus labeling, matching, etc.). The raters are trained by a graduate-student team leader who also regularly reviews the raters' work for quality and provides feedback.

Instructions of rating remained consistent. For more detailed information about the instructions given to raters, please see the instruction sheets included in the "instruct-rater" folder under our Github repository in our original and current submission (https://github.com/xianNeuro/agency-personalizes-episodic-memory).

Reviewer #2 (Remarks to the Author):

This study investigates how agency – specifically, having control over narrative outcomes – modifies episodic memory for complex event sequences. Through interactive "choose-your-own-adventure" stories with varying levels of agency (depending on whether participants were assigned to the Free, Yoked, or Passive conditions), the authors examined how agency influenced recall of narrative events. They found that agency did not merely enhance memory performance, but rather reshaped memory structure to produce more idiosyncratic memory traces.

Overall, this is a very interesting paper, with an interesting study design and a strong analytical approach. It is clearly written and all analyses are well motivated and described. However, given the variety of how agency might influence several other factors along with the subsequent different analyses, the Introduction and Results section feels incredibly dense. I was therefore wondering whether some research questions and their underlying analyses could be moved to the Supplemental Results. In addition to this generic comment, I have a few further major comments, which are summarized below.

> Thank you for this helpful suggestion. In the revised manuscript, we have substantially streamlined the Introduction. We removed the detailed discussions of temporal and causal theories and reorganized the Introduction around a single, clearer through-line: that agency may personalize memory by shifting reliance from generic semantic relations to individualized semantic associations.
>
> Regarding the Results section, we added a new subsection that provides a clear, high-level summary of the Yoked condition. This was added specifically to address your later questions about the Yoked group and to make the role of denied choices easier to understand without requiring readers to parse the more detailed analyses presented in the Supplement. In combination with the streamlined Introduction, we hope that this addition improves the accessibility of the Results.

1) Throughout the Introduction, the authors assert that past research involving agency has typically considered memory for simplistic stimuli (e.g., item lists). Although this claim is somewhat true, it also overlooks studies that have used virtual environments, object exploration games, and/or spatial mazes to show that active control over movement or exploration enhances spatial memory and the temporal organization of experience in naturalistic contexts [research on active vs. passive spatial exploration may be particularly relevant here; Houser et al., 2022 (which is referenced but not discussed in much detail) may also be useful to expand on in that it used a very similar methodology]. I do agree that this study is unique in the sense that it explores memory for a causally-connected series of events with clear narrative structure; however, I think that the Introduction's current framing makes it sound as though this is the first study to explore the effects of agency in the context of complex, naturalistic stimuli, which isn't quite the case.

> Thank you for raising this important point. In the revised Introduction, we expanded our discussion of prior studies using virtual environments, spatial mazes, and object-exploration tasks to provide a more balanced and thorough representation of the agency literature. We now explicitly acknowledge that these paradigms have demonstrated important effects of active control on spatial memory and temporal organization.

At the same time, we clarified why our research question requires a narrative structure with dense and interdependent causal, semantic, and temporal relations—features that these exploratory paradigms typically do not provide. As the reviewer noted, these studies involve naturalistic settings but do not afford the type of richly connected event sequences necessary to examine how agency reshapes which relations among events are encoded and later recalled.

> *"What is known about the role of agency in memory? When people have some level of control over the items that they learn about, they tend to remember more (Gruber & Ranganath, 2019; Hon & Yeo, 2021; Kornell & Metcalfe, 2006; Markant et al., 2014; Murty et al., 2015; Rotem-Turchinski et al., 2019; Ruiz et al., 2023; Voss, Galvan, et al., 2011; Voss, Gonsalves, et al., 2011). While compelling, these findings come largely from studies using lists of isolated and unrelated words, images, or videos as memoranda(Houser et al., 2022; Rotem-Turchinski et al., 2019). Life events, however, do not occur in isolation, nor are they remembered in isolation. Naturalistic experiences unfold as sequences of events, assembling into rich semantic and causal structures that shape later memory (Antony et al., 2024; Chen & Bornstein, 2024; Cohn-Sheehy et al., 2022; Lee & Chen, 2022; Song et al., 2025; Trabasso & Sperry, 1985; Trabasso & Van Den Broek, 1985). As a result, the influence of agency in naturalistic contexts may extend beyond a mere increase in the amount of information remembered. Indeed, active control has been shown to enhance memory for specific aspects of an experience, such as its spatial (Chrastil & Warren, 2012; Qin & Karimi, 2024), temporal (Houser et al., 2022), or causal structure (Kushnir & Gopnik, 2005; Sobel & Kushnir, 2006). Such studies point to the possibility that agency affects which across-event relations we attend to during encoding – potentially changing precisely what we remember about an experience rather than how much (Koriat & Goldsmith, 1996)."*

We believe the revised framing both presents the existing literature more fairly and positions our contribution accurately: not as the first demonstration of agency effects in naturalistic contexts, but as opening new space for understanding how agency influences memory for sequences of richly interconnected naturalistic events.

2) I found it interesting that the authors included two 'non-agentic' conditions: the Yoked condition, wherein choices were sometimes granted through chance, and the Passive condition, wherein participants had no opportunity to make choices at all. To me, the predictions surrounding these two conditions weren't completely clear, which made it a bit confusing as to why both were included. On the one hand, one might expect to see a clear gradient of agentic effects across the three conditions (full agency in the Free condition; partial agency in the Yoked condition; no agency in the Passive condition). On the other hand, I could also imagine participants in the Yoked condition feeling as though they had no agency (because choices were rarely granted), or possibly even experiencing more frustration and more disengagement than participants in the passive group (meaning that, in cases where agency benefits memory, participants in the Yoked condition might even display worse memory than participants in the Passive condition).

We thank the reviewer for raising this thoughtful point about the Yoked condition. Our rationale for including both Yoked and Passive was to separate two different aspects of "non-agency." Both conditions preserve the structural presence of choice points, where the Passive condition provides a baseline of no opportunity to influence the outcome (e.g., students attending lectures, following instructions to turn the book to a certain page, etc.), while the Yoked condition provides an indefinite opportunity to influence the outcome (e.g., requesting to change dorm room which may be granted or denied). The former provides a baseline of no control over the narrative, while

the latter provides a context lacking full control with considerable variability of agency–both were to simulate certain episodes of our real life experiences. We agree with the reviewer that this distinction raises interesting possibilities that warrant more elaboration in the Introduction.

Regarding the reviewer's suggestion that "one might expect to see a clear gradient of agentic effects across the three conditions": We did see support for this idea. Throughout the main results, the Yoked condition exhibited a similar pattern to the Passive condition, with its numerical values falling in between the Free and the Passive condition—consistent with a 'partial agency' or a "clear gradient" prediction. For example, the semantic and causal centrality effect on memory, the idiosyncratic effect, and the neighbor encoding effect across two stories all had the Yoked condition's group mean falling in between the Free and Passive group mean. In all these cases, the Yoked condition aligns more closely with the Passive condition when there is a group difference, i.e., when there's a group difference, there's typically no statistical difference between Yoked and Passive, while Free was the one that stands out.

Regarding the reviewer's suggestion that "participants in the Yoked condition [might feel] as though they had no agency", and thus "might even display worse memory than participants in the Passive condition": Supplementary Results S6 (Supplementary Results S3 in the original submission) includes an analysis focusing on the choice events which were denied, versus choice events which were granted, in the Yoked condition. We used each Yoked subjects' Free condition counterparts (who had all choice events granted) as a comparison group because this is the condition that matched with the Yoked condition across both stories, where subjects can choose either of the two options (passive conditions subjects had no choose but to follow the pre-assigned one). We showed that Yoked subjects' recall performance for choice-denied events was selectively reduced compared to their Free counterparts' recall for the same events, while Yoked subjects' recall performance for choice-granted events was not different from their Free counterparts. This is consistent with what the reviewer raised as a potential frustration or emotional disengagement when one's agency is denied. These results are reported in Supplementary Results S6 "Consequences of having one's choices denied".

Since these are valuable points to make, we added a new results section to the main manuscript to provide more clarity on Yoked condition results as a 'partial agency' (along a gradient of agency) overall, but demonstrates unique memory feature at the local choice events where their agency was denied:

> ***"Consequences of having your choices denied.*** *In the results reported above, Yoked subjects generally exhibited similar memory performance to the Passive subjects—similar idiosyncrasy in event recall, in semantic and causal centrality effect on memory, and in neighbor encoding effects, with their group mean falling in between that of the Free and Passive condition. Given that the Yoked condition had a varied amount of choice granted/denied (see Supplementary Figure S4), these results aligned with the design of 'partial agency' for the Yoked condition in a gradient of agency.*
>
> *Nonetheless, having one's agency denied can have unique memory effects at local choice events: one's memory for the denied choice events is selectively reduced compared to its choice-granted counterparts in the Free condition (Adventure: $p = 0.015$; Romance: $p = 0.017$, two-sample t-test); individual differences contribute to a variation of tendency to selective recall or forget the denied choice events (see Supplement S6 for details).*

The percentage of choices granted in the Yoked participants was not predictive of individual's recall performance, recall similarity to their Free and Passive condition counterparts, semantic centrality effects on memory, nor their neighbor encoding effects (all ps>.3; see Supplement S7 for details); however, higher percentage of choices granted predicted greater individual tendency to forget the choice-denied events (Adventure: r(42) = .335, p = .026. Romance: r(49) = .137, p = .337; see Supplement S6 and S7 for details).

Together, these results suggest that in a context lacking full agentive control, perceived agency and their effects on memory could vary across individuals in non-systematic ways. The one exception is that with more control in such agency-uncertain contexts, the more one has reduced recall for the agency-denied events."

We also pasted the associated supplementary materials below for your convenience (red fonts were new analysis and additions we've made to address your questions):

"S6. Consequences of having one's choices denied.

The number of choices granted to each Yoked participant across their story-path varied depending on the degree of alignment between their choices and those of their Free counterparts. In both stories, there was considerable variation in the percentage of choices granted in Yoked participants. Adventure: range=.29-.83; mean=.53; SE=.02. Romance: range=.45-.83; mean=.62; SE=.01. (Supplementary Figure S4)

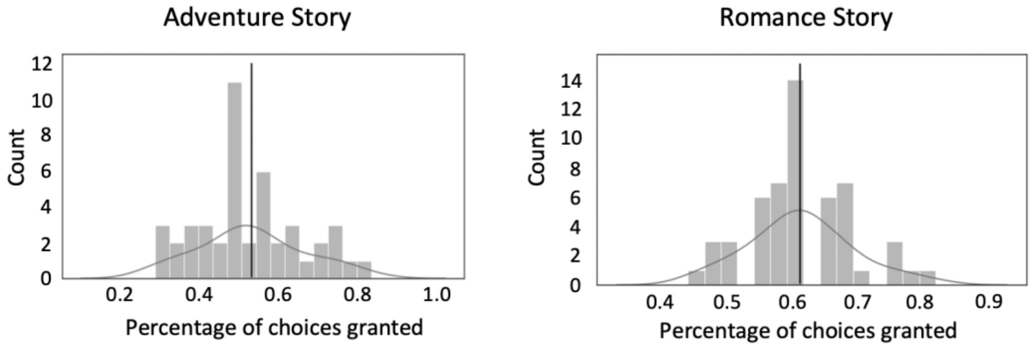

Supplementary Figure S4| Percentage of choices granted to the Yoked participants. *Distributions of overall percentage of choices granted for Yoked participants in the Adventure story (mean=0.53; left) and in the Romance story (mean=0.62; right).*

We examined whether having choices granted vs. denied had consequences for event memory. We separately computed the average recall scores of choice-denied and choice-granted events for each Yoked participant, and of the exact same events for their Free counterparts. For both stories, recall performance was lower for choice-denied events in Yoked participants compared to their Free counterparts (Adventure: p = 0.015; Romance: p = 0.017, two-sample t-test), while recall performance for choice-granted events was not different (ps > 0.2; Supplementary Figure S5).

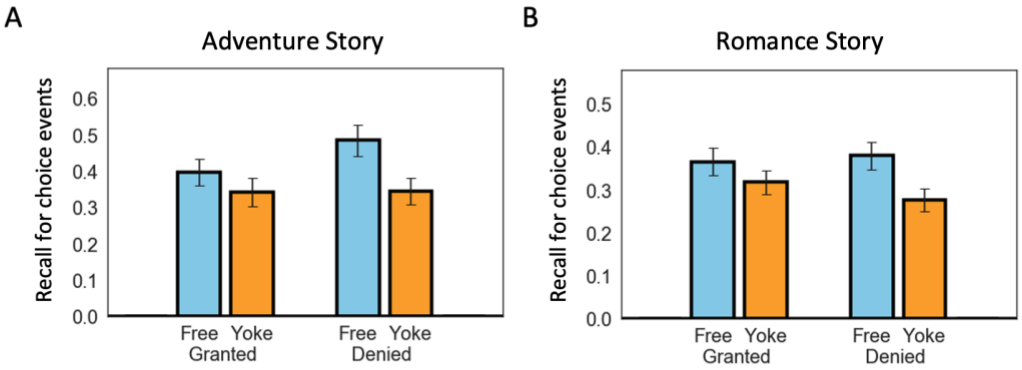

Supplementary Figure S5| Recall performance for choice events that are denied in the yoke condition vs. their free condition counterparts. A, *Adventure story participants' recall performance for granted choice events (left two bars), and for the denied choice events (right two bars). Each Yoked subject has a different collection of choice events denied. We computed each Yoked subject's average recall for events where their choice was denied and computed the average recall of the same events in their matching Free counterparts (where these choice events were always granted). This ensured that the events we compared were exactly the same. The same analysis was performed for granted choice events in Yoked participants and in their Free counterparts. Events for which Yoked participants were denied their choice were less well recalled compared to their Free counterparts, for whom choices were granted; meanwhile, recall for the granted choice events in Yoked participants was comparable to that of their Free counterparts.* ***B,*** *Same as A, for the Romance story.*

We quantified each Yoked participant's tendency to recall or forget denied events as the Pearson correlation between a binary vector (denied/granted = 0/1) and their event-by-event recall vector (0/1). One Adventure participant and two Romance participants were excluded from this analysis for recalling none of the choice events. Correlations centered near zero in both stories (Adventure: $t(43) = -0.076$, $p = 0.939$; Romance: $t(50) = 1.53$, $p = 0.133$; two-sample t-test), indicating substantial individual differences.

To probe these differences, we classified Yoked participants by whether they tended to forget denied events or recall them (Adventure: forget-denied, N = 21; recall-denied, N = 20. Romance: forget-denied, N = 29; recall-denied, N = 22). For denied events, the forget-denied subgroup showed reduced recall relative to Free (Adventure: $t(40) = -2.68$, $p = 0.011$; Romance: $t(56) = -3.66$, $p < .001$), whereas the recall-denied subgroup did not differ from Free (Adventure: $t(38) = -1.10$, $p = 0.279$; Romance: $t(42) = 1.16$, $p = 0.252$). Thus, the overall reduction in Yoked recall for denied events was driven by participants who tended to forget denied events.

These results suggested that the overall reduced memory for choice-denied events among Yoked subjects, compared to their Free counterparts, was likely driven by the sub-sample that tended to forget choice-denied events. These subjects had a significantly greater memory reduction for choice-denied events than the memory improvement in subjects who tended to recall these events, contributing to the overall reduced memory in Yoked subjects for the choice-denied events compared to Free.

S7. Agency effects not as a function of percentage of choices granted.

Since the Yoked subjects had a considerable variation in the percentage of choices granted, could the various memory effects of agency be a function of the percentage of choices granted?

We examined whether the percentage of choices granted across the whole story-path was correlated with overall recall performance, recall of choice-granted events, or recall of choice-denied events. We found no consistent relationship between the

percentage of choices granted and these memory scores across the two stories. All ps>.1 except Adventure: percentage of choices granted vs. choice-denied events' recall r(43)=-.309, p=.039.

We then investigated whether Yoked participants with a higher percentage of granted choices had event-by-event recall more similar to their Free counterparts in the shared sections of the Romance story; however, no significant correlation emerged. r(51)=.100, p=.476. We also examined whether Yoked participants with a lower percentage of granted choices had event-by-event recall more similar to their Passive counterparts in the shared sections of the Romance story; however, no significant correlation emerged. r(51)=.117, p=.404.

Next, we tested whether the percentage of choices granted to Yoked participants was predictive of the magnitude of either the semantic centrality effect or the neighbor encoding effect on memory. We found that the percentage of choices granted to Yoked participants did not correlate with these two effects on memory across the two stories (all ps>.3).

Furthermore, we tested whether the percentage of choices granted to Yoked participants affected their tendency to either forget or recall choice-denied events. One subject from the Adventure story and two from the Romance story were dropped from this correlation analysis due to not recalling any choice events. For both stories, there was a positive correlation between the percentage of choices granted to participants and their tendency to forget choice-denied events, i.e., participants who were granted a higher percentage of choices tended to forget more of the choice-denied events. Adventure: r(42) = .335, p = .026. Romance: r(49) = .137, p = .337. These findings implied that in contexts lacking full agentive control, a higher frequency of granted choices (higher overall agency) was associated with a more pronounced memory reduction for denied choices (choice-denied events).

Overall, these results suggest that the effects of agency on memory is not a function of the percentage of choices granted in the Yoked subjects. This implies that in a context lacking full agentive control, perceived agency and their effects on memory could vary across individuals in non-systematic ways. The one exception is that with more control in such agency-uncertain contexts, the more one has reduced recall for the agency-denied events."

Overall, I think that the introduction would be strengthened by justifying the inclusion of (and potentially making more concrete predictions around) the Yoked and Passive conditions.

We thank the reviewer for this helpful suggestion. In response, we revised the Introduction to more clearly justify the inclusion of both the Yoked and Passive conditions and to articulate the conceptual predictions motivating each. The revised text now explains that these two "non-agentic" conditions capture distinct real-world experiences of lacking control: (1) a baseline condition in which no opportunities for action are available (Passive), and (2) an uncertain control context in which opportunities exist but may be granted or denied (Yoked) that acts as a middle ground (texts pasted below for your convenience):

"While our main comparisons centered on full versus no agency, the Yoked condition provided a meaningful middle ground, enabling us to investigate the effects of degree of control and denied choices on recall."

In addition to clarifying these predictions in the Introduction, we also added a new high-level summary section at the end of the Results specifically for the Yoked condition (as our last response also mentioned; also pasted below for your convenience). This section synthesizes the full pattern of findings: globally, Yoked participants show a “partial agency” profile that numerically sits between Free and Passive and aligns with a gradient-of-agency prediction; at the same time, the Yoked condition exhibits distinct, localized effects at denied-choice events that differ from both Free and Passive. Presenting these features together in a concise summary makes the logic of the Yoked condition—and how it fits into the overall design—much clearer to readers.

> ***“Consequences of having your choices denied.*** *In the results reported above, Yoked subjects generally exhibited similar memory performance to the Passive subjects—similar idiosyncrasy in event recall, in semantic and causal centrality effect on memory, and in neighbor encoding effects, with their group mean falling in between that of the Free and Passive condition. Given that the Yoked condition had a varied amount of choice granted/denied (see Supplementary Figure S4), these results aligned with the design of ‘partial agency’ for the Yoked condition in a gradient of agency.*
>
> *Nonetheless, having one’s agency denied can have unique memory effects at local choice events: one’s memory for the denied choice events is selectively reduced compared to its choice-granted counterparts in the Free condition (Adventure: $p = 0.015$; Romance: $p = 0.017$, two-sample t-test); individual differences contribute to a variation of tendency to selective recall or forget the denied choice events(see Supplement S6 for details).*
>
> *The percentage of choices granted in the Yoked participants was not predictive of individual’s recall performance, recall similarity to their Free and Passive condition counterparts, semantic centrality effects on memory, nor their neighbor encoding effects (all $ps>.3$; see Supplement S7 for details); however, higher percentage of choices granted predicted greater individual tendency to forget the choice-denied events (Adventure: $r(42) = .335$, $p = .026$. Romance: $r(49) = .137$, $p = .337$; see Supplement S6 and S7 for details).*
>
> *Together, these results suggest that in a context lacking full agentive control, perceived agency and their effects on memory could vary across individuals in non-systematic ways. The one exception is that with more control in such agency-uncertain contexts, the more one has reduced recall for the agency-denied events.”*

Together, these revisions provide clearer conceptual grounding for both conditions and more concrete summaries for how Yoked conditions differ from the Passive and the Free conditions. We believe this strengthens the logic of our design and improves the reader’s understanding of why both Yoked and Passive were necessary in addressing our research questions.

Relatedly, throughout the Results section, it wasn’t clear to me whether there were no differences between the Yoked and Passive conditions, or whether potential differences weren’t statistically analyzed. I think this could be made more explicit (even just through a blanket statement early in the Results section to say that all three groups were compared, but that only significant differences between groups are listed).

Thank you for this thoughtful comment. Yes, the Yoked condition aligned with the Passive condition results (i.e., no statistical difference between Yoked and Passive conditions) for most of the memory effects we showed in the results. The results section focused on comparison between the Free and Passive condition, because their group differences were robustly replicated

across stories and methods of analysis. The Yoked condition rarely statistically differed from the Passive condition, and when there is a difference, it's either inconsistent across the two stories (e.g., the semantic centrality effect was reduced in Yoked compared to Passive in the Romance story but not the Adventure story; see Figure 3C), or the methods of analysis (memory idiosyncrasy was enhanced in Yoked compared to Passive when including choice events, but was not different between the two when excluding the choice events; see Figure 2B-C).

To provide full information, we've added a "Statistical tests and reports" paragraph under the Methods section to clarify the analysis conducted and the way of report, pasted below:

> *"**Statistical tests and reports.** Comparisons across conditions included all three conditions (Free, Yoked, Passive) unless otherwise noted. Group mean differences were tested using one-way ANOVAs, with follow-up t-tests conducted only when the overall effect was significant. For analyses involving multiple factors (e.g., semantic vs. causal network effects across conditions), repeated-measures ANOVAs were used, again followed by targeted t-tests when warranted by significant main or interaction effects. Only follow-up comparisons that were significant, consistent across measures, and replicated across both stories are reported in the Results. All t-tests were two-sided. All figures display error bars representing the standard error of the group mean. Significant between-group differences are marked on figures using asterisks, with * for $p < .05$, ** for $p < .01$, and *** for $p < .001$, and trends ($p < .08$) indicated numerically. Full analysis code and test statistic, including all parametric test statistic (e.g. F, t, r) with confidence intervals, effect sizes, degrees of freedom, p-value, and non-parametric tests are provided on GitHub (see Code Availability)."*

Related to the above points:
a. In the yoked condition, how often were choices granted (by chance) on average? How variable was the range of % choices granted across participants?

In the Yoked condition, the proportion of choices granted by chance varied substantially across participants, ranging from approximately 30% to 80% (see Supplementary Fig. S6-1). This variability reflects the probabilistic design of the Yoked condition.

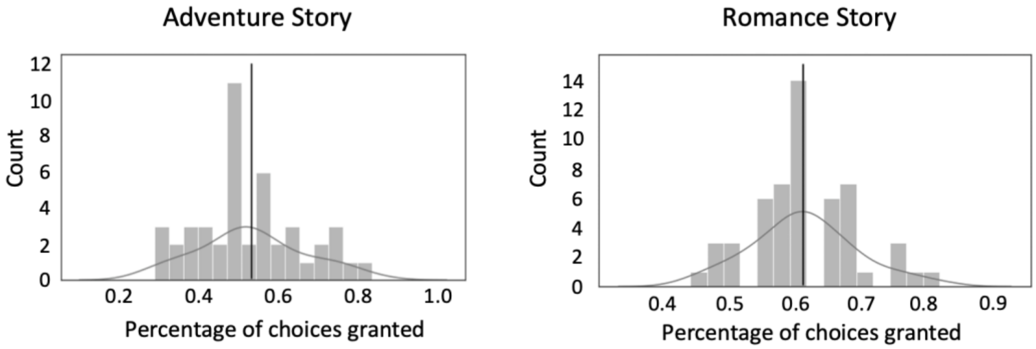

Supplementary Figure S4| Percentage of choices granted to the Yoked participants. Distributions of overall percentage of choices granted for Yoked participants in the Adventure story (mean=0.53; left) and in the Romance story (mean=0.62; right).

I could imagine that participants for which choices were granted more often might show memory patterns closer to the ‘free’ condition whereas participants for which choices were granted only on very few occasions might perform similar to the ‘passive’ or actually even worse than the ‘passive’ condition (see my comment above).
b. Therefore, in the Yoked condition, did performance vary as a function of whether or not choices were granted? (e.g., Maybe memories would be stronger and/or more idiosyncratic among participants for whom a higher % of choices were granted, relative to participants for whom a lower % of choices were granted?)

We thank the reviewer for raising this interesting possibility. In the original manuscript, we tested whether the percentage of granted choices in the Yoked condition predicted memory outcomes, including overall recall, recall for local choice events, recall similarity to their Free counterparts, and the semantic and temporal effects on memory. These analyses did not reveal consistent evidence that the proportion of granted choices accounted for memory performance or recall similarity (see original Supplementary Results, lines 200–224 under S5).

While the results showed that the percentage of choices granted overall did not act as an individual predictor of agency’s effect on memory, we felt it worthwhile to add these null results to the manuscript for clarity. Therefore, we added Supplementary S7 section, which examines the possibility of the percentage of choices granted in Yoked condition as a predictor for agency’s effects on memory (also pasted below for your convenience):

“S7. Agency effects not as a function of percentage of choices granted.

Since the Yoked subjects had a considerable variation in the percentage of choices granted, could the various memory effects of agency be a function of the percentage of choices granted?

We examined whether the percentage of choices granted across the whole story-path was correlated with overall recall performance, recall of choice-granted events, or recall of choice-denied events. We found no consistent relationship between the percentage of choices granted and these memory scores across the two stories. All ps>.1 except Adventure: percentage of choices granted vs. choice-denied events’ recall r(43)=-.309, p=.039.

We then investigated whether Yoked participants with a higher percentage of granted choices had event-by-event recall more similar to their Free counterparts in the shared sections of the Romance story; however, no significant correlation emerged. r(51)=.100, p=.476. We also examined whether Yoked participants with a lower percentage of granted choices had event-by-event recall more similar to their Passive counterparts in the shared sections of the Romance story; however, no significant correlation emerged. r(51)=.117, p=.404.

Next, we tested whether the percentage of choices granted to Yoked participants was predictive of the magnitude of either the semantic centrality effect or the neighbor encoding effect on memory. We found that the percentage of choices granted to Yoked participants did not correlate with these two effects on memory across the two stories (all ps>.3).

Furthermore, we tested whether the percentage of choices granted to Yoked participants affected their tendency to either forget or recall choice-denied events. One subject from the Adventure story and two from the Romance story were dropped from this correlation analysis due to not recalling any choice events. For both stories, there

was a positive correlation between the percentage of choices granted to participants and their tendency to forget choice-denied events, i.e., participants who were granted a higher percentage of choices tended to forget more of the choice-denied events. Adventure: $r(42) = .335$, $p = .026$. Romance: $r(49) = .137$, $p = .337$. These findings implied that in contexts lacking full agentive control, a higher frequency of granted choices (higher overall agency) was associated with a more pronounced memory reduction for denied choices (choice-denied events).

Overall, these results suggest that the effects of agency on memory is not a function of the percentage of choices granted in the Yoked subjects. This implies that in a context lacking full agentive control, perceived agency and their effects on memory could vary across individuals in non-systematic ways. The one exception is that with more control in such agency-uncertain contexts, the more one has reduced recall for the agency-denied events."

c. Given that movement through the story was self-paced, were there differences in reading time across the three conditions (and, if so, is it possible that these differences might influence the other between-group differences that were observed?) I wonder whether Yoked and/or Passive group differences might have had less engagement with the narratives overall, such that shorter reading times might mediate the observed relationships between condition and memory.

We thank the reviewer for this important question. We found no significant group differences in average reading time across the three conditions. Consistent with this, there were also no group differences in overall memory performance, and participants did not differ in their overall engagement, i.e., responses to the transportation survey administered post-experiment. Together, these results indicate that the observed memory effects are not attributable to differences in time on task or general engagement with the narratives.

We added these control analyses to the supplement S3, pasted below:

"For the Romance story, we additionally recorded the reading time for each subject and measured individual engagement over the course of their reading via a 13-item modified version of the Narrative Transportation scale[3]. There were no significant differences across the three agency conditions on participants' transportation score ($F(2,123) = 1.82$, $p = 0.167$), average reading time per story sentence ($F(2,123) = 0.40$, $p = 0.668$), or the overall reading time for the entire story-path they experienced ($F(2,123) = 0.42$, $p = 0.656$). These results suggest that the overall engagement for the story remained roughly the same across the three agency conditions."

We also added a sentence to the results section of the main manuscript, under "Agency did not improve recall performance" section:

"The Romance story additionally recorded individual reading speed and showed no difference across the three agency conditions ($ps > .3$; see Supplement S3 for details)."

3) If I understand correctly, in the recall test, participants were explicitly instructed that completeness was more important than accurate sequencing;

We thank the reviewer for carefully noting the recall instructions. Participants were indeed told that completeness was prioritized over sequencing. The exact instructions were: "Describe the story you read in as much detail as possible. If at any time you realize that you forgot to mention something earlier, go ahead and mention it at that time. Completeness is more important than the order of the story." This instruction ensured that participants focused on recalling as much content as possible, while allowing us to separately analyze recall structure without constraining their responses.

To provide clarity, we have uploaded a demonstration for the recall task page (post detailed instruction for the task) on the GitHub repository (https://github.com/xianNeuro/agency-personalizes-episodic-memory/blob/main/cyoa_demo/recall_instr_demo.html). This is the page where subjects type down what they remember about the story; key reminders stayed on the page as shown.

We also added detailed descriptions under the "Experimental produces" in the Methods section:

> *"Participants typed in a one-line window in which they could see approximately the last 10 words of their recall. Participants were not able to click or scroll through their recall, and they could not backspace past a period, meaning they could only edit the current sentence they were typing. We have implemented an identical procedure in previous work (Lee & Chen, 2022), with the goal of minimizing participants' ability to reorganize, polish or otherwise edit their memories after typing them. This strategy forces typed recall to better approximate spoken recall, where participants cannot change what they have already said aloud."*

additionally, they were only able to delete/edit the most recent 10 typed words (lines 615-623). With this structure in mind, I'm not completely convinced that the analyses surrounding temporal contiguity (lines 348 – 360) are valid. It seems that, by explicitly instructing participants not to worry about event sequencing (and by structuring the recall task such that they can't backtrack and insert later-remembered events into earlier sections of their responses), it would be difficult to draw strong conclusions about whether participants are able to successfully recall temporal event order.

We thank the reviewer for raising this question. We indeed did limit their typed recall such that they were only able to edit the current sentence they were typing (~10 words). We have implemented this strategy in past work as well (Lee & Chen, 2022) as a way to minimize participants' ability to edit or rework their memories after typing them out. Limiting this kind of post-retrieval editing, particularly when dealing with typed memories, is important for two reasons:

(1) We are interested in as faithful an assay as possible of how their experience is spontaneously organized in memory. If we provided participants with a full paragraph-sized text box and only saved whatever was written once they indicated that they were finished, we would lose access to any information about the order in which the retrieved story content came to mind. If participants had been allowed to freely backtrack and reinsert later-remembered events into earlier positions, their written output might have appeared more temporally accurate than their actual recall process, making it impossible to detect out-of-sequence retrievals. To preserve the signal of the spontaneous organization of their memories, we need to maintain some information of the sequence in which this information came to mind. With typed recall, limiting editing to recent words is one way to do this.

(2) This interest in the spontaneous organization in memory is implicit in any task that uses spoken recall. When you recall something, whether a list of words or a complex event, you cannot retract what you have already said. In classic and contemporary free recall paradigms, participants cannot revise earlier responses, yet temporal contiguity is one of the most robust and well-replicated signatures of episodic memory (Bousfield & Sedgewick, 1944; Healey et al., 2014; Kahana, 1996). In piloting typed recall experiments in the lab, we have tried preventing editing entirely, but this was too disruptive for participants. Instead, limiting it to the most recent 10 words was a way to balance preserving spontaneous organization and fluency in participants' ability to perform the task.

We would like to also note that, in spite of our instructions and the limitations we placed on editing recall, participants nevertheless preserved temporal structure to a very high degree: over 90% of recalled events were produced in the correct temporal sequence, with fewer than 10% recalled out of order (see Fig. 4C-D pasted below for your convenience).

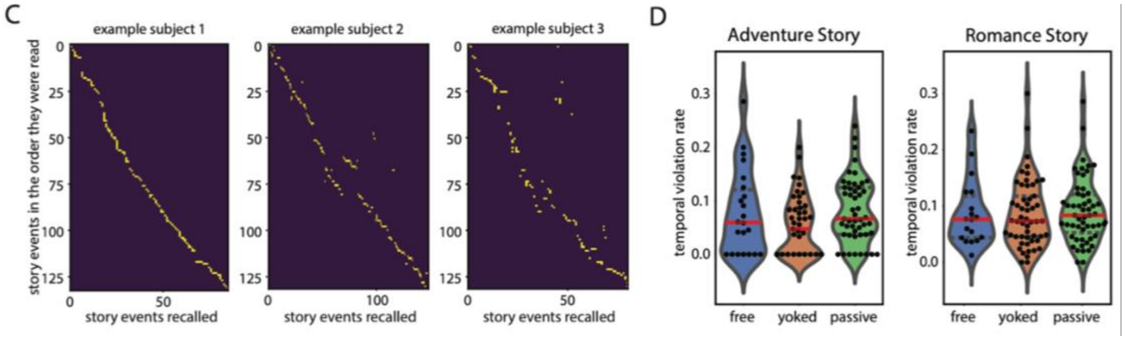

Overall, we believe the back-edits constraints we imposed on this narrative recall task are an essential methodological/manipulation improvement from past approaches, where any edits at any time were allowed, because doing so maximally retains the temporal sequence for the recalled information without much space for post-hoc edits.

In this analysis, there were no-between group differences, but I think that this finding is difficult to interpret (and perhaps better omitted) based on how the recall test instructions were delivered.

We thank the reviewer for raising this concern. We would like to clarify that the recall instructions were identical across all three conditions, so any potential influence of instructions would have applied equally to Free, Yoked, and Passive participants. This consistency ensures that the absence of between-group differences in this analysis cannot be attributed to differences in how the recall task was administered.

Alternatively, it would be good to assure the reader based on previous literature whether this approach is indeed justified to investigate temporal contiguity.

Thank you for this helpful suggestion. Accordingly, we note that the empirical literature on temporal contiguity relies almost exclusively on recall procedures that (i) prioritize completeness over strict sequencing and (ii) do not allow participants to revise earlier responses, particularly in spoken recall. In classic and contemporary free recall paradigms, participants are typically

instructed to recall as many studied items or events as possible “in any order,” and yet strong temporal contiguity effects reliably emerge in the output sequence (Healey et al., 2014; Howard & Kahana, 2002a; Kahana, 1996; Sederberg et al., 2010). In this sense, our typed recall procedure—with limited back-editing—directly mirrors the constraints inherent in spoken recall, for which post-hoc reordering of earlier responses is not possible. Thus, the combination of our instructions (emphasizing completeness) and our recall interface (which preserves the online order in which content comes to mind) is well aligned with standard methods used to investigate temporal contiguity in the free recall literature. The fact that participants in our study nonetheless produced over 90% of recalled events in the correct temporal sequence suggests that our procedure provides a conservative but appropriate assay of temporal organization in memory.

Incorporating this suggestion to the manuscript, we have added the texts below under the “Experimental produces” in the Methods section:

> *“This procedure is consistent with standard free-recall paradigms, in which participants study lists and are later asked to recall as many studied items as possible “in any order,” and robust temporal contiguity effects reliably emerge in the resulting output sequences (Healey et al., 2014; Howard & Kahana, 2002b; Kahana, 1996; Sederberg et al., 2010).”*

Minor points:

1) Did participants pick which story (adventure vs. romance) they wanted to read, or were they assigned to one condition?

We thank the reviewer for asking this clarifying question. The two stories were implemented as separate experiments, rather than as participant choices within the same experiment.

2) The stories roughly had equal numbers of participants in the yoked and passive condition, but quite an imbalance in the free condition (Adventure: N=22; Romance: N=100). Was there a specific reason for this imbalance? And is it possible to add analyses that show that the findings don’t depend heavily on the ‘Romance’ story and therefore bias the findings.

We thank the reviewer for this question and would like to clarify. For all cross-condition comparisons, the Free sample sizes were balanced across stories (Adventure: N = 22; Romance: N = 18). For both stories, we set out to have around 20 Free participants, each of whom was matched to 2–3 Yoked and Passive participants experiencing the identical story path word by word.

The larger Free sample in the Romance dataset (N = 100) that the reviewer noted was collected to ensure adequate coverage of its six structurally distinct story versions (3 branches × 2 endings), and to provide greater power for within-group correlational analyses. From this pool, a subset (N = 18) was selected to form the cross-condition sample directly comparable to the Yoked and Passive groups. All cross-condition comparisons were made with these N=18 Free subjects in the Romance story, because only their story-paths were matched by the Yoked and Passive subjects so that we can fully control for the story texts to be the same across groups.

Thus, there is no imbalance in sample size across conditions for the between-group analyses reported in the manuscript. Moreover, the main effects (matching recall performance, reduced semantic effects on memory, unchanged causal effects, and enhanced neighbor-encoding

effects) observed in the Adventure dataset were independently replicated in the Romance dataset, supporting the robustness and generalizability of our conclusions.

3) In the section on 'Individual Variability in Recalled Events' (starting on line 205), were ISCs calculated against all participants (regardless of condition), or just all other participants in the same condition?

We thank the reviewer for this important clarification question. The ISCs were calculated within each condition only, that is, against all other participants in the same condition. Averaging across all participants regardless of condition would implicitly assume that memory is equally homogeneous both within and across conditions. Because our key research question is precisely whether agency systematically alters which events individual participants remember, it would not be appropriate to mix participants across conditions for this measure.

4) This is a bit pedantic, but I wasn't completely convinced by the examples used to illustrate how agency might activate self-specific knowledge. In particular, the Introduction uses the example of conditioned taste aversion to chocolate ice cream – but, conditioned taste aversions are famously quite strong and automatic, even in cases where participants have no real agency over their choices (e.g., in experiments where rats are administered solutions that will make them sick). I wasn't completely clear on why agency might be expected to influence such a 'fundamental' associative process, and would find it helpful if the authors could highlight specific mechanisms and/or past research that might help strengthen this argument.

Thank you for raising this point. We revised the Introduction to remove the taste-aversion example and replaced it with an example showing how a narrative choice can activate a person's own background knowledge in a way that meaningfully shapes the semantic relations they form during encoding. We believe the new example aligns better with the mechanism we intend to highlight (pasted below for your convenience).

"To illustrate this idea, consider how semantic relations shape episodic memory. Semantic similarity influences both the order and probability of recall, such that semantically related items tend to be retrieved consecutively in free recall tasks (Bousfield, 1953; Manning & Kahana, 2012; Sederberg et al., 2010). Extending this to event memory, Lee & Chen (Lee & Chen, 2022) showed that events that occupied more central positions (i.e., had stronger or more numerous connections) within a story's semantic network were more likely to be recalled. However, in contexts with agency—such as a choose-your-own-adventure narrative—the semantic associations that come to mind during a given event can vary substantially from person to person. For example, chocolate ice cream is often considered a tasty dessert, but if I had recently chosen to poison a batch as a part of an elaborate plot to take down an enemy, it now takes on particular associations for me. This choice would change my semantic associations with "chocolate ice cream", pulling it away from its usual position in generic semantic space towards "poison" instead, and altering how story events featuring chocolate ice cream relate to other events (Bornstein et al., 2017; Yee & Thompson-Schill, 2016). In this way, agency may highlight an individual's idiosyncratic experiences and knowledge, causing memory to rely less on the kinds of generic semantic relations typically derived from the large corpora(Barsalou, 1987; Cer et al., 2018; Mikolov et al., 2013; Yee & Thompson-Schill, 2016) and more on personalized semantic associations unique to each individual."

Reviewer #2 (Remarks on code availability):

I did not review the code.

Reviewer #3 (Remarks to the Author):

I co-reviewed this manuscript with one of the reviewers who provided the listed reports. This is part of the Nature Communications initiative to facilitate training in peer review and to provide appropriate recognition for Early Career Researchers who co-review manuscripts.

Reviewer #3 (Remarks on code availability):

I may be missing it, but was not able to find the codes through this link or on the OSF repository

Thank you for checking. In our original submission, we provided the rater instructions, story stimuli, raw experimental data, and scripts for reproducing the manuscript figures on our GitHub repository (https://github.com/xianNeuro/cyoa.git). Reviewer #4 was able to reproduce the figures using these scripts, though noted that several file paths needed to be adjusted to match the repository structure.

Since then, in response to comments raised during review, we have re-run all analyses in multiple ways to confirm the robustness of the results. To ensure full transparency and ease of access, we have consolidated all materials in a new repository (https://github.com/xianNeuro/agency-personalizes-episodic-memory), which now includes separate **figures** and **stats_code** folders containing the updated figure-generation scripts, complete statistical analysis code, and test outputs (using Pearson *r* values as reported in the manuscript, as well as Fisher's *z*–transformed values).

Reviewer #4 (Remarks to the Author):

The authors investigated how control over an episodic narrative affected different aspects of memory (specifically, for temporal, causal, and semantic relations within the narrative). Participants read stories, with within-story "events" presented sequentially and the degree of control (agency) being manipulated gradually by allowing some participants to make active choices regarding the story progression (that were either always granted or sometimes granted and sometimes not) or not providing participants the chance to make active choices. While agency did not affect overall recall performance or the impact of causal connections between events on memory, it increased interindividual differences in narrative recall, suggesting that agency reorganized memories to more strongly reflect personal experiences.

The manuscript addresses a contemporary topic – the interplay between agency and (episodic) memory – while distinguishing between different memory aspects, which is novel considering previous research. I also think that the agency manipulation is an interesting one that, for example, extends on Ruiz et al. (2023). The presented results are likely interesting and relevant for researchers in the domains of memory, action control, social cognition, and control experience more generally.

Previous literature is referenced appropriately, and the manuscript gives an adequate overview of relevant research. I have suggested some additional literature that may be interesting to consider below.

> We sincerely thank the reviewer for these encouraging words about our work.

I have some concerns regarding the reliability and validity of some of the analyses (see below) that should be addressed.

Regarding data & methodology, I have the following concerns and questions:

• Why was there such a big discrepancy in the sample sizes in the Free condition between the Adventure and Romance stories.

> We thank the reviewer for this question and would like to clarify. While there were differences in the number of Free participants across both the Adventure and Romance stories, the Free sample sizes were similar across stories for all cross-condition comparisons (Adventure: N = 22; Romance: N = 18).
>
> The Romance story was designed with 3 main branches and 2 possible endings (6 combinations), with the goal of diversifying the story content across participants (i.e., branches 1, 2, and 3 had the protagonist visiting different locations with different co-characters). We wanted to have a balance of participants across these 6 combinations, but because participants in the Free condition determined their own story paths, we could not pre-assign them to a specific branch-ending combination. Therefore, to ensure adequate coverage of all branch-ending combinations, we needed to collect a larger Free sample for the Romance story (N = 100). From this pool, we then selected three participants from each branch-ending combination to form a balanced Free sample (N = 18); these 18 story-paths were then used to generate the perfectly-matched-text Yoked and Passive conditions. Thus, while the total Free pool was larger in the Romance dataset, the Free samples used for cross-condition analyses were comparable in size across the two stories.

The larger sample of N=100 was used for analyses that did not require comparison across conditions, such as the correlation analysis under results section “Greater memory divergence was associated with weaker semantic influence and stronger temporal dependence”, where correlation tests were conducted for both the N=18 selected Free subjects as well as the N=100 Free condition full subjects pool for greater statistical power to detect significant association (e.g., between individual’s semantic centrality effect and memory idiosyncrasy).

• From my understanding, the Adventure story was less well controlled than the Romance story, and the corresponding results do not add much to the manuscript. Consider moving the Adventure story results to the Supplement. This would also make the manuscript more concise.

We thank the reviewer for raising this point. While it’s true that the Adventure story lacks some key control features, we believe it is important to present both datasets in the main text. The Adventure story differed from the Romance story in narrative context, the overall story length, story-path variations, number of choices available, etc. These differences between the two experiments help demonstrate that the observed effects are generalizable and robust across different narrative contexts, as opposed to being specific to an individual story. For these reasons, we prefer to retain both datasets in the main text.

• The way the different conditions are set up, the predictions or expectations regarding the event following a choice trial were sometimes violated only in the Yoked condition. This factor may affect memory. Based on this, could one infer alternative explanations for the observed data patterns? Please discuss.

We thank the reviewer for this insightful point. A very similar question was asked by Reviewer #2. Indeed, in the Yoked condition participants sometimes experienced denied choices, where the outcome violated their expectation. This was precisely why we included three levels of agency in the design: Free (full agency), Yoked (partial agency with occasional denial), and Passive (no agency at all). The Yoked condition allowed us to ask whether such moments of denied choice might affect memory.

Supplementary Results S6 (Supplementary Results S3 in the original submission) includes an analysis focusing on the choice events which were denied, versus choice events which were granted, in the Yoked condition. We used each Yoked subject’s Free condition counterpart (who had all choice events granted) as a comparison group because these counterparts read identical stories. We showed that Yoked subjects’ recall performance for choice-denied events was selectively reduced compared to their Free counterparts' recall for the same events, while Yoked subjects’ recall performance for choice-granted events was not different from their Free counterparts. This is consistent with what the reviewer raised as a potential frustration or emotional disengagement when one’s agency is denied. These results are reported in Supplementary Results S6 "Consequences of having one’s choices denied".

Since these are valuable points to make, we added a new results section to the main manuscript to provide more clarity on Yoked condition, explicitly framing it as a condition with ‘partial agency’ (along a gradient of agency). In it, we further unpack the consequences of denied vs. granted choices on memory, as suggested by the reviewer:

> ***“Consequences of having your choices denied.*** *In the results reported above, Yoked subjects generally exhibited similar memory performance to the Passive subjects—similar idiosyncrasy in event recall, in semantic and causal centrality effect on memory,*

and in neighbor encoding effects, with their group mean falling in between that of the Free and Passive condition. Given that the Yoked condition had a varied amount of choice granted/denied (see Supplementary Figure S4), these results aligned with the design of 'partial agency' for the Yoked condition in a gradient of agency.

Nonetheless, having one's agency denied can have unique memory effects at local choice events: one's memory for the denied choice events is selectively reduced compared to its choice-granted counterparts in the Free condition (Adventure: p = 0.015; Romance: p = 0.017, two-sample t-test); individual differences contribute to a variation of tendency to selective recall or forget the denied choice events(see Supplement S6 for details).

The percentage of choices granted in the Yoked participants was not predictive of individual's recall performance, recall similarity to their Free and Passive condition counterparts, semantic centrality effects on memory, nor their neighbor encoding effects (all ps>.3; see Supplement S7 for details); however, higher percentage of choices granted predicted greater individual tendency to forget the choice-denied events (Adventure: r(42) = .335, p = .026. Romance: r(49) = .137, p = .337; see Supplement S6 and S7 for details).

Together, these results suggest that in a context lacking full agentive control, perceived agency and their effects on memory could vary across individuals in non-systematic ways. The one exception is that with more control in such agency-uncertain contexts, the more one has reduced recall for the agency-denied events."

We also pasted the associated supplementary materials below for your convenience (red fonts were new analysis and additions we've made to fully address your question):

"S6. Consequences of having one's choices denied.

The number of choices granted to each Yoked participant across their story-path varied depending on the degree of alignment between their choices and those of their Free counterparts. In both stories, there was considerable variation in the percentage of choices granted in Yoked participants. Adventure: range=.29-.83; mean=.53; SE=.02. Romance: range=.45-.83; mean=.62; SE=.01. (Supplementary Figure S4)

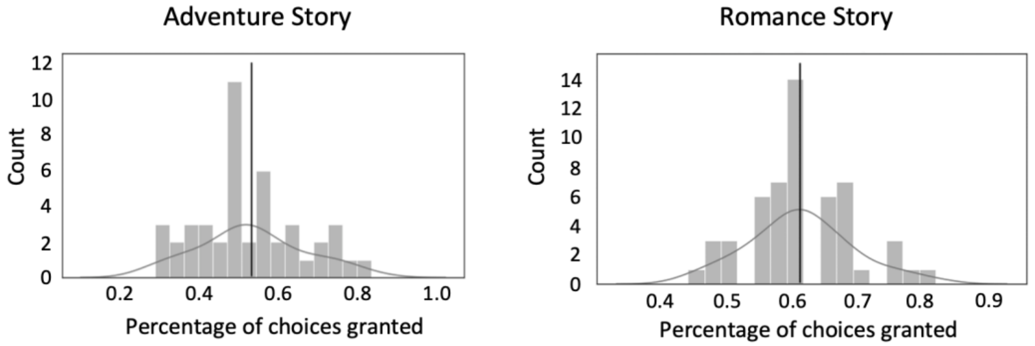

Supplementary Figure S4| Percentage of choices granted to the Yoked participants. *Distributions of overall percentage of choices granted for Yoked participants in the Adventure story (mean=0.53; left) and in the Romance story (mean=0.62; right).*

We examined whether having choices granted vs. denied had consequences for event memory. We separately computed the average recall scores of choice-denied and choice-granted events for each Yoked participant, and of the exact same events for their Free counterparts. For both stories, recall performance was lower for choice-denied events

in Yoked participants compared to their Free counterparts (Adventure: $p = 0.015$; Romance: $p = 0.017$, two-sample t-test), while recall performance for choice-granted events was not different (ps > 0.2; Supplementary Figure S5).

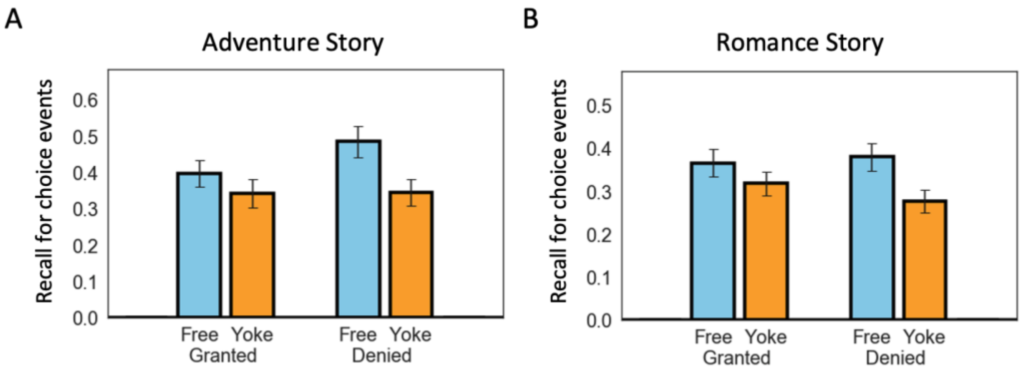

Supplementary Figure S5| Recall performance for choice events that are denied in the yoke condition vs. their free condition counterparts. A, *Adventure story participants' recall performance for granted choice events (left two bars), and for the denied choice events (right two bars). Each Yoked subject has a different collection of choice events denied. We computed each Yoked subject's average recall for events where their choice was denied and computed the average recall of the same events in their matching Free counterparts (where these choice events were always granted). This ensured that the events we compared were exactly the same. The same analysis was performed for granted choice events in Yoked participants and in their Free counterparts. Events for which Yoked participants were denied their choice were less well recalled compared to their Free counterparts, for whom choices were granted; meanwhile, recall for the granted choice events in Yoked participants was comparable to that of their Free counterparts.* ***B,*** *Same as A, for the Romance story.*

We quantified each Yoked participant's tendency to recall or forget denied events as the Pearson correlation between a binary vector (denied/granted = 0/1) and their event-by-event recall vector (0/1). One Adventure participant and two Romance participants were excluded from this analysis for recalling none of the choice events. Correlations centered near zero in both stories (Adventure: $t(43) = -0.076$, $p = 0.939$; Romance: $t(50) = 1.53$, $p = 0.133$; two-sample t-test), indicating substantial individual differences.

To probe these differences, we classified Yoked participants by whether they tended to forget denied events or recall them (Adventure: forget-denied, N = 21; recall-denied, N = 20. Romance: forget-denied, N = 29; recall-denied, N = 22). For denied events, the forget-denied subgroup showed reduced recall relative to Free (Adventure: $t(40) = -2.68$, $p = 0.011$; Romance: $t(56) = -3.66$, $p < .001$), whereas the recall-denied subgroup did not differ from Free (Adventure: $t(38) = -1.10$, $p = 0.279$; Romance: $t(42) = 1.16$, $p = 0.252$). Thus, the overall reduction in Yoked recall for denied events was driven by participants who tended to forget denied events.

These results suggested that the overall reduced memory for choice-denied events among Yoked subjects, compared to their Free counterparts, was likely driven by the sub-sample that tended to forget choice-denied events. These subjects had a significantly greater memory reduction for choice-denied events than the memory improvement in subjects who tended to recall these events, contributing to the overall reduced memory in Yoked subjects for the choice-denied events compared to Free.

S7. Agency effects not as a function of percentage of choices granted.

Since the Yoked subjects had a considerable variation in the percentage of choices granted, could the various memory effects of agency be a function of the percentage of choices granted?

We examined whether the percentage of choices granted across the whole story-path was correlated with overall recall performance, recall of choice-granted events, or recall of choice-denied events. We found no consistent relationship between the percentage of choices granted and these memory scores across the two stories. All ps>.1 except Adventure: percentage of choices granted vs. choice-denied events' recall r(43)=-.309, p=.039.

We then investigated whether Yoked participants with a higher percentage of granted choices had event-by-event recall more similar to their Free counterparts in the shared sections of the Romance story; however, no significant correlation emerged. r(51)=.100, p=.476. We also examined whether Yoked participants with a lower percentage of granted choices had event-by-event recall more similar to their Passive counterparts in the shared sections of the Romance story; however, no significant correlation emerged. r(51)=.117, p=.404.

Next, we tested whether the percentage of choices granted to Yoked participants was predictive of the magnitude of either the semantic centrality effect or the neighbor encoding effect on memory. We found that the percentage of choices granted to Yoked participants did not correlate with these two effects on memory across the two stories (all ps>.3).

Furthermore, we tested whether the percentage of choices granted to Yoked participants affected their tendency to either forget or recall choice-denied events. One subject from the Adventure story and two from the Romance story were dropped from this correlation analysis due to not recalling any choice events. For both stories, there was a positive correlation between the percentage of choices granted to participants and their tendency to forget choice-denied events, i.e., participants who were granted a higher percentage of choices tended to forget more of the choice-denied events. Adventure: r(42) = .335, p = .026. Romance: r(49) = .137, p = .337. These findings implied that in contexts lacking full agentive control, a higher frequency of granted choices (higher overall agency) was associated with a more pronounced memory reduction for denied choices (choice-denied events).

Overall, these results suggest that the effects of agency on memory is not a function of the percentage of choices granted in the Yoked subjects. This implies that in a context lacking full agentive control, perceived agency and their effects on memory could vary across individuals in non-systematic ways. The one exception is that with more control in such agency-uncertain contexts, the more one has reduced recall for the agency-denied events."

Regarding whether the above findings might provide alternative explanations for the observed data patterns: The central results of this manuscript focus on the contrast between Free and Passive conditions, examing the effect of having agency versus having none at all. The consistent finding that Free participants' memories were more idiosyncratic than Passive participants' cannot be attributed to denied choices (expectation violation), because this only occurred in the Yoked condition, not Passive. Thus, while the Yoked condition provides an interesting intermediate case, the key results of the manuscript rest on the Free vs. Passive comparison.

• A more serious concern that I have is whether the temporal dependency measure may be confounded with interindividual variations in memory performance. The authors generate a vector of neighbor recall performance, including person- and event-specific average recalls scores for recalling previous and subsequent events, and correlate this vector with the original recall performance vector for each participant. If I have a participant that performs pretty well overall, this participant is likely to have high entries in both the neighbor recall performance and the original recall vector. resulting in a high correlation. However, this is not necessarily indicative of temporal dependency but could simply be attributed to high memory performance more generally (a similar argument can be made for lower levels of performance). The authors should elaborate on this issue (for a discussion on the role of performance when analyzing dependencies for item sets see also Horner & Burgess, 2013 , JEP: General, Schreiner & Meiser, 2023, Behavior Research Methods, and Yu et al. (2025, Memory & Cognition).

> We thank the reviewer for this excellent point. Intuitively, participants who recall more events overall are more likely to show higher apparent temporal contiguity, simply because with more recalled events there are more opportunities for recalled events to fall next to each other. This is a valid concern for interpreting neighbor-encoding effects in isolation.
>
> However, in our datasets, overall recall performance was matched across the Free, Yoked, and Passive conditions in both stories. Participants in all three groups recalled, on average, the same number of events, meaning they had comparable opportunities for neighboring events to be recalled. Despite this equivalence in recall quantity, Free participants showed significantly stronger neighbor-encoding effects than Yoked or Passive participants. In other words, while a Free and a Passive participant might each recall ~30 events from the same narrative, the Free participant's recalls tended to cluster next to one another in temporal order more than the Passive participant's recalls. This pattern indicates that the agency increased the organization of recall beyond what would be expected from memory performance alone.
>
> To provide clarity, we've added a short paragraph to explain our additional control measures before we performed the neighbor encoding effect comparisons across conditions:
>
>> *"Because the neighbor encoding effect is computed using correlations, a potential concern is that it might be biased by individual differences in their overall memory performance. To rule out this possibility, we checked that the overall recall performance was statistically equivalent across the Free, Yoked, and Passive conditions in both stories (see Supplementary Figure S2)."*

• In the paragraph starting on line 186 (section "Agency did not improve recall performance"), it is mentioned that independent raters compared each sentence. I assume this relates to "Matching recall to story-paths" in the Methods section. However, I am missing the information on how many raters there were. Also, what was the interrater reliability and how were inconsistencies in ratings resolved? (this is also relevant for other ratings)

> We thank the reviewer for this question and are glad to clarify. Our laboratory maintains a team of 5-8 raters (undergraduate research assistants) who perform tasks such as this routinely, as we have similar needs across many of our experiments (speech transcription, stimulus labeling, matching, etc.). The raters are trained by a graduate-student team leader who also regularly reviews the raters' work for quality and provides feedback.

Across the two stories, we collected recall from a total of 324 participants (Adventure: N = 116; Romance: N = 208), spanning 22 Adventure versions and 100 Romance versions. Recall matching was performed by the team of trained raters. Raters first read the story in full, then read each recall file, and matched recall segments to the corresponding story events. This was a time-intensive process: rating one Adventure recall typically took 30–60 minutes, while rating a Romance recall took 60–180 minutes, amounting to roughly 500 hours of coding in total.

To maximize consistency, each story-path's recall files (Free, Yoked, Passive) were assigned to the same rater for the Romance dataset, ensuring that cross-condition comparisons were not confounded by rater differences. Raters were blind to experimental conditions, and their only task was to align recall segments with story events. All raters received the same recall rating instructions (available on Github under "instruct-raters" in the original submission).

Regarding reliability, recall matching is considered largely objective (whether a recalled phrase corresponds to a given story event); thus, as in prior studies using similar procedures (Chen et al., 2017; Zadbood et al., 2017), a single rater is used for each story. In our case, when training raters, we reviewed examples with raters before assigning stories, and any ambiguities in labeling were discussed until consensus was reached. Because the same rater scored all conditions of a given story-path (Free, Yoked, Passive), rater variability in judgments is unlikely to explain the condition-level differences we report.

To provide clarity, we've added a short paragraph to explain rater training and instructions:

> *"Human ratings for naturalistic stimuli*
> *Our laboratory maintains a team of raters (research assistants) who routinely perform tasks pertaining to processing and ratings of naturalistic stimuli, as we have similar needs across many of our experiments (speech transcription, stimulus labeling, matching, etc.). The raters are trained by a graduate-student team leader who also regularly reviews the raters' work for quality and provides feedback. They followed a standardized written instruction set (publicly available in the "instruct-raters" folder on GitHub; see Data Availability), ensuring consistency across coders."*

The reviewer's point is also relevant to a new paragraph of the Discussion which explicitly acknowledges the current reliance on human coding in narrative memory research. We also highlight the exciting potential of large language models to provide scalable, automated annotation in future work, while noting that questions of objectivity and bias will remain important to evaluate:

> *"A necessary aspect of research using naturalistic stimuli such as narratives is that labeling of many stimulus and behavioral response features relies on human judgments: e.g., in the current study, delineation of event boundaries, causal links between events, segmentation of participants' recall and assignment of those segments to the story events. For each such variable, researchers must choose between 1) having the features "curated" by the researchers themselves or by trained labelers, 2) estimating population-level human judgments by running a separate experiment (commonly performed for event boundary selection), or 3) some combination. The "curation" approach avoids some of the messiness which comes with human data, while the "separate experiment" approach may allow for scientific claims arising from the human judgment data. In our study, we largely took the curation approach (see Methods). Interestingly, recent publications have demonstrated that LLMs can perform some of these judgment tasks with comparable-to-human*

performance (e.g., recall-to-story content matching (Georgiou et al., 2025; Raccah et al., 2024) and event segmentation (Michelmann et al., 2025; Panela et al., 2025)). As the field moves toward adopting such automated annotation pipelines, the high-quality human-rated data from the current study will serve as a valuable resource for training and validating these future systems. However, it is also important to note that the judgments of LLMs may not be more "objective" than those of humans; certain judgments have no correct answer, but rather should be expected to generate a distribution of responses from humans. It would be a mistake to replace human ratings with automated tools if doing so creates an illusion that the cognition underlying those judgments is well-understood (Crockett & Messeri, 2023)."

The authors report a comprehensive set of analyses that mostly appear to be sound. However, I see the following issues:

• Error bars are not defined (e.g., in Figure 2B, Figure S3).

Thank you for pointing this out. All figures include error bars representing the standard error of the group mean, and we have now added a sentence in the Methods under a new section "Statistical tests and reports" clarifying this:

"All figures display error bars representing the standard error of the group mean."

• Test statistics should be reported for all tests (e.g., in lines 211 and 212 no t values are reported).

Thank you for noting this. Reporting full statistics for all tests across two stories and three conditions for each analysis would substantially lengthen the Results section and reduce readability. To maintain clarity and narrative flow, we follow the reporting style commonly used in Nature Communications, where one-sample tests are often summarized by test type and p-value rather than by listing t-statistics for each instance. For example, Reagh and Ranganath (Reagh & Ranganath, 2023) describe the use of one-sample t-tests in their Methods but summarize the corresponding results in the main text through effect estimates and p-values rather than explicit t-values.

For transparency and readers' full information, we have made all analysis code publicly available on GitHub (https://github.com/xianNeuro/agency-personalizes-episodic-memory), including scripts that reproduce every statistical test reported in the manuscript. These scripts print out the full statistical results—including all t-values.

To provide clarity, we have added a sentence in the Methods:

"Full analysis code and test statistic, including all parametric test statistic (e.g. F, t, r) with confidence intervals, effect sizes, degrees of freedom, p-value, and non-parametric tests are provided on GitHub (see Code Availability)."

• Many analyses rely on Pearson correlations that are subjected to t tests. Correlations tend to be skewed, which is why Fisher's Z transformation is usually applied when averaging correlations. Are the reported results robust when rerunning the analyses based on Z-transformed correlation coefficients?

Thank you for the thoughtful comment. We followed the reviewer's suggestion and reran all analyses in the Results section that involve parametric tests on Pearson correlation values using Fisher's Z-transformed coefficients. All statistical conclusions reported in the manuscript remain unchanged. For example, inter-subject memory similarity remained significantly above zero across all conditions when tested on Fisher Z-transformed values (e.g., original Free condition mean r = .136, t = 11.746, p < .001; fisher z-transformed mean z = .140, t = 11.672, p < .001), where Free condition was significantly reduced compared to the Yoked (original Free vs. Yoked: t = -7.285, p < .001; fisher z-transformed t = -7.190, p < .001) and the Passive condition (original Free vs. Passive: t = -9.731, p < .001; fisher z-transformed t = -9.496, p < .001).

A comprehensive report is shared on GitHub (https://github.com/xianNeuro/agency-personalizes-episodic-memory/tree/main) under "*stats_code/output/comprehensive_report/*" in case you would like to further examine the statistical outputs using the fisher z-transformed values pertaining to parametric tests on Pearson correlations. The report provides the corresponding Z-means, t-stats, F-stats, and p-values for all correlation-based parametric tests and shows that each result is consistent with the statistical conclusions reported in the current manuscript using raw Pearson r values.

We have added a section to the Methods:

> *"Averaging correlations.*
> *In all instances where we performed statistical tests involving Pearson correlation, we additionally conducted the same tests but with a Fisher z-transformation to stabilize the variance across the distribution of correlation values. These additional analyses confirmed the results reported in the current manuscript. Full analysis code and statistical outputs, including the Fisher r-to-z transformed Z-test results on the correlation values are provided on GitHub (see Data Availability)."*

Inferences and conclusions adequately follow the reported results. I found the Discussion to be comprehensible. However, the authors could extend on the theoretical implications of their findings.

In part, I found the manuscript somewhat difficult to access, particularly the Results section, though I did like the Discussion. Some comments:

• I found the Results section in part hard to follow. I suggest better matching the reporting to the overarching research question and matching reporting to the order of the different subquestions/memory aspects in the Introduction.

Thank you for this suggestion. To address the concern that parts of the Results were hard to follow, we rewrote the Introduction so that it now sets up a clear expectation for the key analyses that appear first in the Results—namely, the personalized memory effects and the semantic analyses. The revised Introduction focuses on establishing why these aspects are central to our research question and prepares readers for these results in a more direct way.

Because the Introduction and Results now follow the same conceptual order, readers can enter the Results section with a clearer sense of what to expect and why each analysis is presented. We believe this alignment improves the accessibility and overall flow of the Results without changing the analytic structure itself.

• The authors could make a bit clearer which results they initially anticipated and what were more exploratory tests. This is perhaps related to the previous point.

Thank you for this helpful comment. In the revised manuscript, we clarified which results were central to our initial predictions and how the remaining analyses relate to those predictions. The Introduction now explicitly sets up our main expectations regarding personalized memory and semantic influences, which are the analyses presented first in the Results.

At the same time, we also had several additional predictions that could not be fully developed in the streamlined Introduction without making it dense again. For example: (a) we predicted that memory for denied-choice events in the Yoked condition might increase (if denied choices created a prediction error) or might decrease (if reduced agency led to disengagement or suppression); (b) we predicted that agency could increase the influence of causal centrality on memory, since having control over what happens next might prompt participants to focus more on causal chains between events; and (c) we considered that agency might strengthen temporal sequencing at recall, although in richly connected narratives temporal order was already very strong in all conditions, including Passive.

Because these predictions were not the main conceptual focus of the current manuscript, we instead unpacked these aspects of the data in the Discussion and, where appropriate, provided further analyses in the Supplement. This structure allows the main theoretical through-line to remain clear while still giving readers full access to the reasoning and results behind these additional, preregistered predictions.

• I believe the paragraph starting on line 99 could be better connected to the previous one.

Thank you for pointing this out. In the original submission, the paragraph introducing personalized memory did not transition smoothly from its previous paragraph regarding semantic associations. In the revised Introduction, we rewrote this section to more clearly link the two ideas (texts from introduction pasted below for your convenience).

> *"To illustrate this idea, consider how semantic relations shape episodic memory. Semantic similarity influences both the order and probability of recall, such that semantically related items tend to be retrieved consecutively in free recall tasks (Bousfield, 1953; Manning & Kahana, 2012; Sederberg et al., 2010). Extending this to event memory, Lee & Chen (Lee & Chen, 2022) showed that events that occupied more central positions (i.e., had stronger or more numerous connections) within a story's semantic network were more likely to be recalled. However, in contexts with agency—such as a choose-your-own-adventure narrative—the semantic associations that come to mind during a given event can vary substantially from person to person. For example, chocolate ice cream is often considered a tasty dessert, but if I had recently chosen to poison a batch as a part of an elaborate plot to take down an enemy, it now takes on particular associations for me. This choice would change my semantic associations with "chocolate ice cream", pulling it away from its usual position in generic semantic space towards "poison" instead, and altering how story events featuring chocolate ice cream relate to other events (Bornstein et al., 2017; Yee & Thompson-Schill, 2016). In this way, agency may highlight an individual's idiosyncratic experiences and knowledge, causing memory to rely less on the kinds of generic semantic relations typically derived from the*

large corpora(Barsalou, 1987; Cer et al., 2018; Mikolov et al., 2013; Yee & Thompson-Schill, 2016) and more on personalized semantic associations unique to each individual.

The extent to which agency changes what or how much we remember, particularly in the context of richly interconnected naturalistic events, remains unclear. In traditional paradigms, agency has been shown to boost the quantity of remembered details, possibly because making choices is inherently rewarding (Fujiwara et al., 2013; Leotti & Delgado, 2011) and such motivational factors can enhance memory encoding (Shohamy & Adcock, 2010). Additionally, agency may increase the self-relevance of the current input (Hon & Yeo, 2021), which could boost episodic memory by encouraging integration of the input with existing networks of self-relevant knowledge – a form of deeper elaborative processing (Catrambone et al., 1996; Markus, 1977; Rogers et al., 1977; Symons & Johnson, 1997). What do these ideas suggest about how agency might alter memory for sequences of inter-connected events? Since making choices requires agency, one may predict enhanced memory for events that involve making a choice. Furthermore, since agency activates self-relevant knowledge, it could amplify inter-individual differences in memory. For example, two friends exploring the main street of a new city together might visit shops together in the same sequence, but have different motivations for doing so (e.g., historical vs. food tour). The two friends would experience distinct framings for how the shops relate to one another; two "personalized" networks of associations across time would be generated, making different shops memorable or forgettable for each person (Anderson & Pichert, 1978). In this way, agency need not increase the amount of information remembered wholesale, but could instead shift which relations among events are formed or attended to in the process of organizing them. This idea that agency might personalize individuals' memory has yet to be tested."

Overall, we now explain how semantic relations normally guide memory and then directly connect this to how agency can shift those semantic associations in a personalized direction. The revised paragraph explicitly ties semantic centrality, individualized semantic associations, and the expected personalizing effect of agency into a single, continuous line of reasoning. We believe this restructuring improves conceptual flow and makes the connection between semantic influences and personalized memory much clearer.

• I found the paragraph starting on line 128, for the preliminary results summary at the end of the introduction, hard to understand without first reading the Results section. Could this paragraph be made more comprehensible without having to refer to the results section?

Thank you for pointing this out. In the original submission, the preliminary summary paragraph at the end of the Introduction was harder to interpret because the preceding sections did not clearly prepare the reader for the types of analyses that followed. In the revised manuscript, we rewrote the Introduction so that it now lays out the conceptual motivations, predictions, and analytic logic much more explicitly. Per noted in the previous response, the sections leading up to the summary now introduce (i) why agency may personalize memory, (ii) why semantic influences might change under agency, and (iii) why the three conditions (Free, Yoked, Passive) were included.

Because these ideas are now clearly established earlier in the Introduction, the final summary paragraph—which briefly previews the main findings—is much easier to understand on its own

and no longer requires readers to have already seen the Results. We believe the rewritten version resolves the issue by making the paragraph self-contained and aligned with the expectations set by the new Introduction.

(Further) comments to the authors:

• The relationship between events and event sequences or episodic narratives could be spelled out a bit more clearly. In this context, the framework by Andermane et al. (2021, Current Opinion in Neurobiology) may be useful to consider.

Thank you for this helpful suggestion. In the revised Introduction, we clarified the distinction between individual events and the larger event sequences that make up an episodic narrative. We now explicitly emphasize that naturalistic experiences involve interconnected events embedded within broader semantic and causal structures, and that agency may influence memory not only for individual moments but also for how those moments relate to one another within the unfolding narrative. Strengthening this explanation helps clarify why a narrative-based paradigm is necessary for testing our central question about personalized memory.

• (Minor) It may be interesting to connect the present research to the enactment effect and to the generation effect.

Thank you for the interesting suggestion. The enactment effect (enhancement of memory when actions are performed oneself, as opposed to when observed) and the generation effect (enhancement of memory when one generates information oneself, as opposed to when it is merely presented) seem most relevant to the "choice" events, wherein participants make a choice (Free condition) or view the same choice being made outside their control (Passive, and to a lesser extent, Yoked). In our new analysis focusing on choice events (Supplementary section S4; pasted below for your convenience), we found that for choice events in the Romance story, memory was better for the Free as compared to Yoked or Passive conditions [Free > Yoked (t = 2.43, p = 0.016), Free > Passive (t = 1.99, p = 0.048)].

"S4. Recall for choice and non-choice events

Memory for choice events is significantly reduced compared to the other events ($p < .001$). The choice events in our paradigm differ in nature from the other (non-choice) narrative events—they describe specific actions that participants can select and thus are much shorter (around 5 words) than non-choice events (~40–50 words), making them less likely to be recalled in absolute terms. However, when recall is normalized by the number of words in each event, we see the opposite pattern: participants recall choice events more often per word than normal story events. This was true across all agency conditions, even for the Passive condition.

We compared recall score for non-choice versus choice events across the three agency conditions (Free, Yoked, Passive). Importantly, because Yoked and Passive participants read the story paths generated by Free participants, event content was matched across conditions; only the degree of perceived agency varied. For both stories, memory performance did not differ significantly across conditions (Adventure: $F(2,113) = 1.76$, $p = 0.177$; Romance: $F(2,205) = 1.80$, $p = 0.167$). Recall performance for non-choice events was significantly higher than for choice events across all conditions for both stories (Adventure: $F(1,113) = 430.23$, $p < .001$; Romance: $F(1,205) = 722.06$, $p < .001$). There

was a significant agency condition × event type interaction for the Romance story ($F(2, 205) = 12.77$, $p < .001$), and a trend for the Adventure story ($F(2, 113) = 2.76$, $p = 0.068$). For the Romance story, post-hoc comparisons for choice events revealed: Free > Yoked ($t = 2.43$, $p = 0.016$), Free > Passive ($t = 1.99$, $p = 0.048$).

We next compared recall by word performance for non-choice versus choice events across the three agency conditions (Free, Yoked, Passive). Across the two stories, we did not observe consistent differences across conditions (Adventure: $F(2,113) = 1.37$, $p = 0.258$; Romance: $F(2,205) = 3.48$, $p = 0.033$). Recall by word performance for choice events was significantly higher than for non-choice events (Adventure: $F(1,113) = 165.41$, $p < .001$; Romance: $F(1,205) = 459.21$, $p < .001$). There was a significant agency condition × event type interaction for the Romance story ($F(2, 205) = 4.45$, $p = 0.013$), but not for the Adventure story ($F(2,113) = 1.85$, $p = 0.163$). For the Romance story, post-hoc comparisons for choice events revealed: Free > Yoked in recall per word ($t = 2.46$, $p = 0.015$), Free > Passive in recall per word ($t = 2.09$, $p = 0.038$).

Together, these results showed that the choice events were better recalled than non-choice events when we normalized recall performance by word count. This is consistent with the idea that making a choice could improve memory—even in the passive condition, the simple physical action of clicking a choice button but not having an actual ability to choose otherwise could still improve memory. Further, the enhanced memory for choice events was most prominent in the free condition, where subjects had the most agency. This suggests that perceived agency may further boost recall per word beyond the physical action of selection (clicking or dragging a button).

The result that Free participants recalled choice events better than matched participants in Yoked and Passive conditions echoes classic findings in the study of human memory: the enactment effect, wherein memory is enhanced when actions are performed oneself, as opposed to when observed (Engelkamp, 1998); and the generation effect, wherein memory is enhanced when one generates information oneself, as opposed to when it is merely presented (Bertsch et al., 2007).

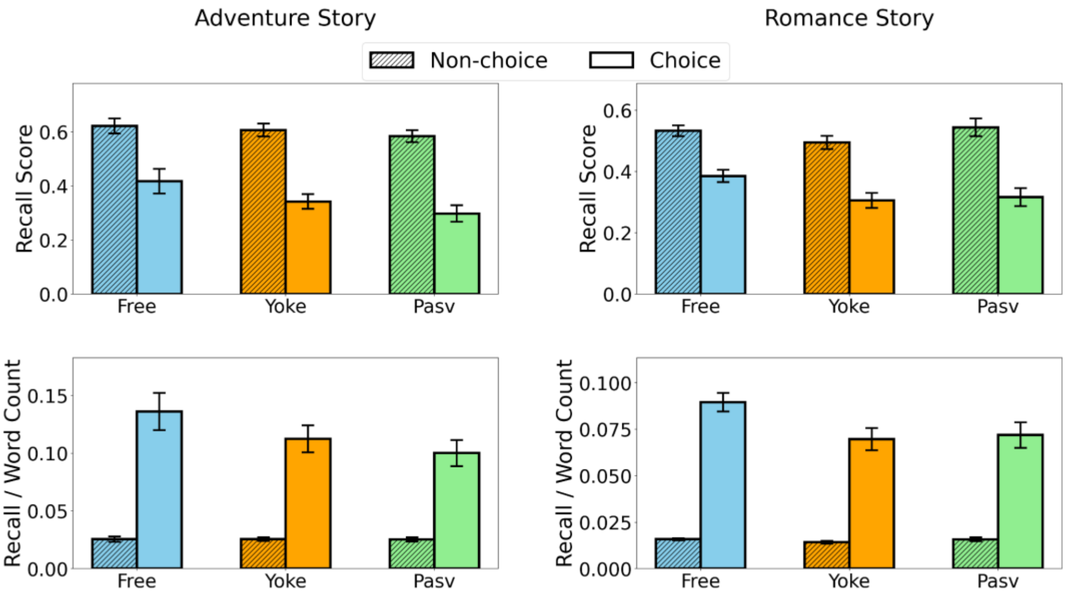

***Supplementary Figure S3| Recall and recall per word split by choice and non-choice event.** The figure displays two measures organized in rows: Recall Score (top row) and Recall per Word (bottom row). For each measure, the left column shows data from the Adventure story, and the right column shows data from the*

Romance story. Each panel shows six bars representing the three agency conditions (Free: sky blue, Yoked: orange, Passive: light green) × two event types (Non-choice: hatched bars with diagonal slashes, Choice: solid bars). Error bars represent standard error of the mean across subjects. The figure demonstrates that choice events showed higher recall per word than non-choice events across all conditions and both stories, with agency condition differentially affecting memory for choice versus non-choice events in the Romance story."

• (Minor) Note that, for Ref. 8 (Hon & Yeo, 2021), participants could not "choose" the identity of items they produced, but what was manipulated was the compatibility of a spatial effect dimension to a participant's action, which was then followed by the item identity, and items in compatible action-effect episodes were remembered better than items in incompatible ones (see also Schreiner et al., 2025, Memory & Cognition, but see Tsuji & Imaizumi, 2022, Scientific Reports). One may refer to this as control, but I think "choosing" the item identity does not quite capture it.

Thank you for noting this. We updated the language in the Introduction to more accurately describe the body of literature on agency that covers the study of Hon & Yeo (Hon & Yeo, 2021), ensuring that our phrasing reflects the forms of control that can also encompass this study while describing other agency studies cited (pasted below for your convenience).

"When people have some level of control over the items that they learn about, they tend to remember more (Gruber & Ranganath, 2019; Hon & Yeo, 2021; Kornell & Metcalfe, 2006; Markant et al., 2014; Murty et al., 2015; Rotem-Turchinski et al., 2019; Ruiz et al., 2023; Voss, Galvan, et al., 2011; Voss, Gonsalves, et al., 2011)."

• (Minor) In the context of the finding that agency did not affect recall performance it may also be interesting to look at findings by Tsuji and Imaizumi (2022, Scientific Reports, and Schreiner et al., 2024, Quarterly Journal of Experimental Psychology). I saw that the findings by Tsuji and Imaizumi were mentioned in the Supplement though.

We appreciate the reviewer's point here, and have added mention of this to the Results:

"Agency did not improve recall performance. *For each participant, events were binned according to whether they were remembered or forgotten. Independent raters compared each sentence of recall to the story path read by the participant; if any part of a given event was mentioned in any recall sentence, it was counted as remembered. There were no significant differences in recall performance across conditions in either story (Adventure: $F(2,113)=1.43$, $p=0.243$; Romance: $F(2,123)=0.67$, $p=0.513$; Supplementary Figure S2). The Romance story additionally recorded individual reading speed and showed no difference across the three agency conditions (ps> .3; see Supplement S3 for details). See Supplement S4 for details about memory for choice and non-choice events; See Supplement S5 for details about recognition memory performance; See Supplement S6 for details about memory for denied and granted choice events. Overall, increased agency did not result in a greater number of remembered details, unlike previous work using random-item list-learning paradigms (Gruber & Ranganath, 2019; Hon & Yeo, 2021; Kornell & Metcalfe, 2006; Markant et al., 2014; Murty et al., 2015; Rotem-Turchinski et al., 2019; Ruiz et al., 2023; Voss, Galvan, et al., 2011; Voss, Gonsalves, et al., 2011); instead, this result adds to the set of studies showing that agency's enhancing effects on memory depend on details of the content, experimental paradigm, and population (Schreiner et al., 2024; Tani et al., 2025; Tsuji & Imaizumi, 2022)."*

• Please explain the different colors in Figure S7 in the figure note.

The Figure S7 in the original submission corresponds to S11 in this submission–we added in the caption clarification for the colors in both S11 and S12.

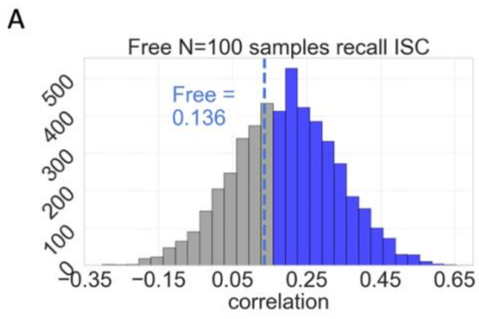

Supplementary Fig. S9| Recall and choice ISC distribution of full Free sample (N=100) with mean for selected N=18. A, *Distribution of recall ISC values across all 100 Free participants, calculated from the 64 shared events in the Romance story. The vertical blue dashed line marks the mean recall ISC (r = 0.136, p > 0.1) for the 18 selected Free participants whose story paths were assigned to the Yoke and Passive conditions. Samples with greater group mean than the selected Free subjects were colored blue.* ***B,*** *Distribution of choice ISC values across all 100 Free participants, calculated from the 15 choice events in the shared sections of the Romance story. The vertical blue dashed line marks the mean choice ISC (r = 0.208, p > 0.1) for the same 18 Free participants. Samples with greater group mean than the selected Free subjects were colored blue.*

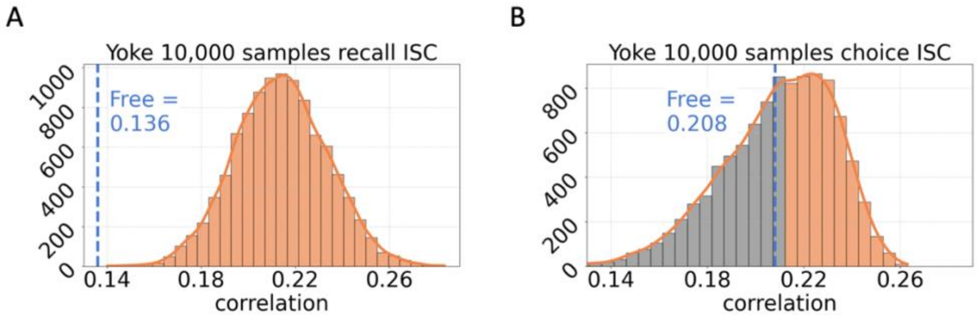

Supplementary Fig. S12| Recall ISC distribution for 10,000 Yoked samples matched on choice ISC to the selected 18 Free participants. A, *Distribution of mean recall ISC for the 10,000 yoked samples (orange histogram), each matching the selected Free participants' story paths and choice ISC. The blue dashed line marks the selected Free participants' mean recall ISC (r = 0.136, p = 0.000). Recall ISC was calculated from the 64 shared story events in the Romance story. Samples with greater group mean than the selected Free subjects were colored orange.* ***B,*** *Distribution of mean choice ISC for the same 10,000 yoked samples, with the blue dashed line marking the 18 selected Free participants' mean choice ISC (mean r = 0.208, p = 0.423). Choice ISC was calculated from the 15 shared choice events in the Romance story. Note that the choice ISC was used as selection criteria to draw samples from the Yoked condition with matching choice behaviors to the 18 Free participants. Samples with greater group mean than the selected Free subjects were colored orange.*

• (Minor) Line 406 is missing an "of" before "causal relations".

Thank you for noticing–we have corrected it.

Reviewer #4 (Remarks on code availability):

The linked repository contains python code to reproduce the figures. However, please double-check that the file paths match the structure of the repository, for example, in "1_plot_bars.ipynb" I had to remove "data" from file_path = './1_bar_plots/data/bar_data.xlsx'. This would increase usability.

Thank you for noticing–we have corrected them in the new repository:
https://github.com/xianNeuro/agency-personalizes-episodic-memory

I only found code to reproduce the figures but not code for the different statistical tests. Is this code available somewhere?

In addressing several statistical comments from the reviewers, we have re-run all our analysis in several different ways to confirm the results reported in the current manuscript. Please see "stats_code" under our new github repository (https://github.com/xianNeuro/agency-personalizes-episodic-memory) for the statistical analysis code and full test reports (using Pearson r-values as reported in the manuscript, and using Fisher's Z transformed values) to reproduce results reported in the manuscript.

Footnote:

A small number of statistical values changed slightly (all within < .01) due to differences across software packages used in the original manuscript and the revision. For example, the Free vs. Yoked memory ISC comparison changed from $p = 0.006$ to $p = 0.007$. All such updates are clearly noted in the revised manuscript.

References:

For the convenience of the reviewers, we provide below a separate list of all papers cited in the current response letter in APA style. Relevant references have been incorporated into the revised manuscript with updated reference numbers in Nature style.

Anderson, R. C., & Pichert, J. W. (1978). Recall of previously unrecallable information following a shift in perspective. *Journal of Verbal Learning and Verbal Behavior*, *17*(1), 1–12. https://doi.org/10.1016/S0022-5371(78)90485-1

Antony, J., Lozano, A., Dhoat, P., Chen, J., & Bennion, K. (2024). Causal and chronological relationships predict memory organization for nonlinear narratives. *Journal of Cognitive Neuroscience*, 1–18. https://doi.org/10.1162/jocn_a_02216

Baldassano, C., Chen, J., Zadbood, A., Pillow, J. W., Hasson, U., & Norman, K. A. (2017). Discovering Event Structure in Continuous Narrative Perception and Memory. *Neuron*, *95*(3), 709-721.e5. https://doi.org/10.1016/j.neuron.2017.06.041

Barsalou, L. W. (1987). The instability of graded structure: Implications for the nature of concepts. In *Concepts and conceptual development: Ecological and intellectual factors in categorization* (pp. 101–140). Cambridge University Press.

Bertsch, S., Pesta, B. J., Wiscott, R., & McDaniel, M. A. (2007). The generation effect: A meta-analytic review. *Memory & Cognition*, *35*(2), 201–210. https://doi.org/10.3758/BF03193441

Bornstein, A. M., Khaw, M. W., Shohamy, D., & Daw, N. D. (2017). Reminders of past choices bias decisions for reward in humans. *Nature Communications*, *8*(1), 15958. https://doi.org/10.1038/ncomms15958

Bousfield, W. A. (1953). The occurrence of clustering in the recall of randomly arranged associates. *The Journal of General Psychology*, *49*(2), 229–240. https://doi.org/10.1080/00221309.1953.9710088

Bousfield, W. A., & Sedgewick, C. H. W. (1944). An Analysis of Sequences of Restricted Associative Responses. *The Journal of General Psychology*. https://www.tandfonline.com/doi/abs/10.1080/00221309.1944.10544467

Catrambone, R., Beike, D., & Niedenthal, P. (1996). Is the self-concept a habitual referent in judgments of similarity? *Psychological Science*, *7*(3), 158–163. https://doi.org/10.1111/j.1467-9280.1996.tb00349.x

Cer, D., Yang, Y., Kong, S., Hua, N., Limtiaco, N., St. John, R., Constant, N., Guajardo-Cespedes, M., Yuan, S., Tar, C., Strope, B., & Kurzweil, R. (2018). Universal sentence encoder for english. In E. Blanco & W. Lu (Eds.), *Proceedings of the 2018 Conference on Empirical Methods in Natural Language Processing: System Demonstrations* (pp. 169–174). Association for Computational Linguistics. https://doi.org/10.18653/v1/D18-2029

Chen, J., & Bornstein, A. M. (2024). The causal structure and computational value of narratives. *Trends in Cognitive Sciences*, *28*(8), 769–781. https://doi.org/10.1016/j.tics.2024.04.003

Chen, J., Leong, Y. C., Honey, C. J., Yong, C. H., Norman, K. A., & Hasson, U. (2017). Shared memories reveal shared structure in neural activity across individuals. *Nature Neuroscience*, *20*(1), 115–125. https://doi.org/10.1038/nn.4450

Chrastil, E. R., & Warren, W. H. (2012). Active and passive contributions to spatial learning. *Psychonomic Bulletin & Review*, *19*(1), 1–23. https://doi.org/10.3758/s13423-011-0182-x

Cohn-Sheehy, B. I., Delarazan, A. I., Crivelli-Decker, J. E., Reagh, Z. M., Mundada, N. S., Yonelinas, A. P., Zacks, J. M., & Ranganath, C. (2022). Narratives bridge the divide between distant events in episodic memory. *Memory & Cognition*, *50*(3), 478–494. https://doi.org/10.3758/s13421-021-01178-x

Crockett, M., & Messeri, L. (2023). *Should large language models replace human participants?* https://osf.io/4zdx9_v1

Engelkamp, J. (1998). *Memory for actions* (pp. vii, 166). Psychology Press/Taylor & Francis (UK).

Flores, S., Bailey, H. R., Eisenberg, M. L., & Zacks, J. M. (2017). Event segmentation improves event memory up to one month later. *Journal of Experimental Psychology: Learning, Memory, and Cognition*, *43*(8), 1183–1202. https://doi.org/10.1037/xlm0000367

Fujiwara, J., Usui, N., Park, S. Q., Williams, T., Iijima, T., Taira, M., Tsutsui, K.-I., & Tobler, P. N. (2013). Value of freedom to choose encoded by the human brain. *Journal of Neurophysiology*, *110*(8), 1915–1929. https://doi.org/10.1152/jn.01057.2012

Furman, O., Dorfman, N., Hasson, U., Davachi, L., & Dudai, Y. (2007). They saw a movie: Long-term memory for an extended audiovisual narrative. *Learning & Memory*, *14*(6), 457–467. https://doi.org/10.1101/lm.550407

Georgiou, A., Can, T., Katkov, M., & Tsodyks, M. (2025). Large-scale study of human memory for meaningful narratives. *Learning & Memory*, *32*(2), a054043. https://doi.org/10.1101/lm.054043.124

Gold, D. A., Zacks, J. M., & Flores, S. (2017). Effects of cues to event segmentation on subsequent memory. *Cognitive Research: Principles and Implications*, *2*(1), 1. https://doi.org/10.1186/s41235-016-0043-2

Gruber, M. J., & Ranganath, C. (2019). How curiosity enhances hippocampus-dependent memory. *OSF Preprints*. https://doi.org/10.31219/osf.io/5v6nm

Healey, M. K., Crutchley, P., & Kahana, M. J. (2014). Individual Differences in Memory Search and Their Relation to Intelligence. *Journal of Experimental Psychology. General*, *143*(4), 1553–1569. https://doi.org/10.1037/a0036306

Hon, N., & Yeo, N. (2021). Having a sense of agency can improve memory. *Psychonomic Bulletin & Review*, *28*(3), 946–952. https://doi.org/10.3758/s13423-020-01849-x

Houser, T. M., Tompary, A., & Murty, V. P. (2022). Agency enhances temporal order memory in an interactive exploration game. *Psychonomic Bulletin & Review*, *29*(6), 2219–2228. https://doi.org/10.3758/s13423-022-02152-7

Howard, M. W., & Kahana, M. J. (2002a). A Distributed Representation of Temporal Context. *Journal of Mathematical Psychology*, *46*(3), 269–299. https://doi.org/10.1006/jmps.2001.1388

Howard, M. W., & Kahana, M. J. (2002b). A Distributed Representation of Temporal Context. *Journal of Mathematical Psychology*, *46*(3), 269–299. https://doi.org/10.1006/jmps.2001.1388

Kahana, M. J. (1996). Associative retrieval processes in free recall. *Memory & Cognition*, *24*(1), 103–109. https://doi.org/10.3758/BF03197276

Koriat, A., & Goldsmith, M. (1996). Memory metaphors and the real-life/laboratory controversy: Correspondence versus storehouse conceptions of memory. *Behavioral and Brain Sciences*, *19*(2), 167–188. https://doi.org/10.1017/S0140525X00042114

Kornell, N., & Metcalfe, J. (2006). Study efficacy and the region of proximal learning framework. *Journal of Experimental Psychology: Learning, Memory, and Cognition*, *32*(3), 609–622. https://doi.org/10.1037/0278-7393.32.3.609

Kushnir, T., & Gopnik, A. (2005). Young Children Infer Causal Strength From Probabilities and Interventions. *Psychological Science*, *16*(9), 678–683. https://doi.org/10.1111/j.1467-9280.2005.01595.x

Lee, H., & Chen, J. (2022). Predicting memory from the network structure of naturalistic events. *Nature Communications*, *13*(1), 4235. https://doi.org/10.1038/s41467-022-31965-2

Leotti, L. A., & Delgado, M. R. (2011). The inherent reward of choice. *Psychological Science*. https://doi.org/10.1177/0956797611417005

Manning, J. R., & Kahana, M. J. (2012). Interpreting semantic clustering effects in free recall. *Memory*, *20*(5), 511–517. https://doi.org/10.1080/09658211.2012.683010

Markant, D., DuBrow, S., Davachi, L., & Gureckis, T. M. (2014). Deconstructing the effect of self-directed study on episodic memory. *Memory & Cognition*, *42*(8), 1211–1224. https://doi.org/10.3758/s13421-014-0435-9

Markus, H. (1977). Self-schemata and processing information about the self. *Journal of Personality and Social Psychology*, *35*(2), 63–78. https://doi.org/10.1037/0022-3514.35.2.63

Michelmann, S., Kumar, M., Norman, K. A., & Toneva, M. (2025). Large language models can segment narrative events similarly to humans. *Behavior Research Methods*, *57*(1), 39. https://doi.org/10.3758/s13428-024-02569-z

Mikolov, T., Yih, W., & Zweig, G. (2013). Linguistic regularities in continuous space word representations. In L. Vanderwende, H. Daumé III, & K. Kirchhoff (Eds.), *Proceedings of the 2013 Conference of the North American Chapter of the Association for Computational Linguistics: Human Language Technologies* (pp. 746–751). Association for Computational Linguistics. https://aclanthology.org/N13-1090

Murty, V. P., DuBrow, S., & Davachi, L. (2015). The simple act of choosing influences declarative memory. *Journal of Neuroscience*, *35*(16), 6255–6264. https://doi.org/10.1523/JNEUROSCI.4181-14.2015

Panela, R. A., Barnett, A. J., Barense, M. D., & Herrmann, B. (2025). *Event Segmentation Applications in Large Language Model Enabled Automated Recall Assessments* (No. arXiv:2502.13349). arXiv. https://doi.org/10.48550/arXiv.2502.13349

Qin, Y., & Karimi, H. A. (2024). Active and passive exploration for spatial knowledge acquisition: A meta-analysis. *Quarterly Journal of Experimental Psychology*, *77*(5), 964–982. https://doi.org/10.1177/17470218231185121

Raccah, O., Chen, P., Gureckis, T. M., Poeppel, D., & Vo, V. A. (2024). The “Naturalistic Free Recall” dataset: Four stories, hundreds of participants, and high-fidelity transcriptions. *Scientific Data*, *11*(1), 1317. https://doi.org/10.1038/s41597-024-04082-6

Reagh, Z. M., & Ranganath, C. (2023). Flexible reuse of cortico-hippocampal representations during encoding and recall of naturalistic events. *Nature Communications*, *14*(1), 1279. https://doi.org/10.1038/s41467-023-36805-5

Rogers, T. B., Kuiper, N. A., & Kirker, W. S. (1977). Self-reference and the encoding of personal information. *Journal of Personality and Social Psychology*, *35*(9), 677–688. https://doi.org/10.1037/0022-3514.35.9.677

Rotem-Turchinski, N., Ramaty, A., & Mendelsohn, A. (2019). The opportunity to choose enhances long-term episodic memory. *Memory*, *27*(4), 431–440. https://doi.org/10.1080/09658211.2018.1515317

Ruiz, N. A., DuBrow, S., & Murty, V. P. (2023). Agency as a bridge to form associative memories. *Journal of Experimental Psychology: General*, *152*(6), 1797–1813. https://doi.org/10.1037/xge0001356

Schreiner, M. R., Bröder, A., & Meiser, T. (2024). Agency effects on the binding of event elements in episodic memory. *Quarterly Journal of Experimental Psychology*, *77*(6), 1201–1220. https://doi.org/10.1177/17470218231203951

Sederberg, P. B., F. Miller, J., W. Howard, M., & J. Kahana, M. (2010). The temporal contiguity effect predicts episodic memory performance. *Memory & Cognition*, *38*(6), 689–699. https://doi.org/10.3758/MC.38.6.689

Shohamy, D., & Adcock, R. A. (2010). Dopamine and adaptive memory. *Trends in Cognitive Sciences*, *14*(10), 464–472. https://doi.org/10.1016/j.tics.2010.08.002

Sobel, D. M., & Kushnir, T. (2006). The importance of decision making in causal learning from interventions. *Memory & Cognition*, *34*(2), 411–419. https://doi.org/10.3758/BF03193418

Song, H., Lu, Q., Nguyen, T. T., Chen, J., Leong, Y. C., Rosenberg, M. D., Ching, S., & Zacks, J. M. (2025). *A neural network with episodic memory learns causal relationships between narrative events* (p. 2025.09.01.673596). bioRxiv. https://doi.org/10.1101/2025.09.01.673596

Symons, C. S., & Johnson, B. T. (1997). The self-reference effect in memory: A meta-analysis. *Psychological Bulletin*, *121*(3), 371–394. https://doi.org/10.1037/0033-2909.121.3.371

Tani, N., Olson, I. R., & Newcombe, N. S. (2025). The Limits of Agency: Young Children's Memory May Not Benefit From Choice. *Collabra: Psychology*, *11*(1), 137316. https://doi.org/10.1525/collabra.137316

Trabasso, T., & Sperry, L. L. (1985). Causal relatedness and importance of story events. *Journal of Memory and Language*, *24*(5), 595–611. https://doi.org/10.1016/0749-596X(85)90048-8

Trabasso, T., & Van Den Broek, P. (1985). Causal thinking and the representation of narrative events. *Journal of Memory and Language*, *24*(5), 612–630. https://doi.org/10.1016/0749-596X(85)90049-X

Tsuji, N., & Imaizumi, S. (2022). Sense of agency may not improve recollection and familiarity in recognition memory. *Scientific Reports*, *12*(1), 21711. https://doi.org/10.1038/s41598-022-26210-1

Voss, J. L., Galvan, A., & Gonsalves, B. D. (2011). Cortical regions recruited for complex active-learning strategies and action planning exhibit rapid reactivation during memory retrieval. *Neuropsychologia*, *49*(14), 3956–3966. https://doi.org/10.1016/j.neuropsychologia.2011.10.012

Voss, J. L., Gonsalves, B. D., Federmeier, K. D., Tranel, D., & Cohen, N. J. (2011). Hippocampal brain-network coordination during volitional exploratory behavior enhances learning. *Nature Neuroscience*, *14*(1), 115–120. https://doi.org/10.1038/nn.2693

Yee, E., & Thompson-Schill, S. L. (2016). Putting concepts into context. *Psychonomic Bulletin & Review*, *23*(4), 1015–1027. https://doi.org/10.3758/s13423-015-0948-7

Zadbood, A., Chen, J., Leong, Y. C., Norman, K. A., & Hasson, U. (2017). How We Transmit Memories to Other Brains: Constructing Shared Neural Representations Via

Communication. *Cerebral Cortex*, *27*(10), 4988–5000.

https://doi.org/10.1093/cercor/bhx202

We greatly appreciate the reviewers' positive and constructive comments. Throughout the response letter, we use consistent color-coding and formatting to indicate added text, revisions made in response to reviewer comments, and changes made for clarity: black fonts are the verbatim comments from reviewers, blue fonts being our responses, *italicized black fonts* are quotes of our documents from the original submission (manuscript, supplement), and *italicized red fonts* marking new additions.

Reviewer #1 (Remarks to the Author):

I appreciate the authors' thorough and thoughtful responses to the comments raised in my original review. Here are remaining issues with the authors' responses:

Comment1:
In the original review, I wrote that "Memory performance for the choice events themselves seems to be very low, can the authors explain the reason for this? (Figure 2A)."
The authors replied -
We thank the reviewer for highlighting this important detail. The choice events in our paradigm differ in nature from the other (non-choice) narrative events—they describe specific actions that participants can select and thus are much shorter (around 5 words) than non-choice events (~40–50 words). As the reviewer noticed, this brevity makes them less likely to be recalled in absolute terms. However, when recall is normalized by the number of words in each event, we see the opposite pattern: participants recall choice events more often per word than normal story events (see our new analysis in the figure below). This suggests that, although brief, choice events are encoded relatively well, consistent with the idea that making a choice improves memory.
We have added this new figure to the Supplement in section S4, Figure S3: "Memory for choice and non-choice events controlling for word count".

I appreciate the authors' response and the additional analysis; however, I am not fully convinced that it directly addresses the concern raised in my original comment. As described in the text, the stories were segmented into events, and participants' recall responses were subsequently assigned to these events. In the revised analysis, the authors appear to assume a direct relationship between the number of words in the original narrative and the likelihood of later recall, normalizing recall (binary) by word count. However, text length may not be a primary determinant of subsequent memory performance. Rather, I would expect that words associated with the opportunity to make a choice would be better remembered than words from non-choice periods, largely independent of text length. Demonstrating such an effect would more directly support the interpretation that agency enhances memory, which was the central issue raised in my original comment.

> We thank the reviewer for the careful follow-up and for articulating the remaining source of confusion so clearly. We agree that this point is important, particularly given this novel paradigm. We believe the disconnect arises from a mismatch between the reviewer's reasonable expectation of what constitutes a "choice event" in agency–memory paradigms and how choice points are implemented and scored in our task.
>
> This study was not designed to test whether choice content is better remembered than non-choice content. Instead, our central goal was to examine how agency alters the *organization and personalization* of memory for naturalistic narratives. Accordingly, in our paradigm, choice events and non-choice narrative events are intentionally not matched in length, informational density, or functional role.

Choice events are brief, interface-driven action prompts (e.g., “Don’t tell me.”; “You raise your hand.”) whose purpose is to enable interaction and steer the narrative, whereas non-choice events are extended narrative segments containing rich descriptive and causal information. Because recall was scored at the event level using a binary criterion (any mention of an event counted as recalled), longer narrative events naturally have a higher probability of being credited as recalled in free recall, independent of encoding strength. For this reason, a direct comparison of absolute recall rates between choice and non-choice events cannot be interpreted as a clean test of agency-related memory enhancement. To provide a concrete example, in the excerpt below, Events 101 and 104 are choice events, each consisting of only a few words, embedded within much longer narrative events:

Event	***Choice***	***Story Text***
99		As soon as you enter the party, you note the classic sticky floor, active game of beer pong and clusters of sweaty dancers. Lex screams, throwing her arm around a group of her sorority sisters.
100		One girl breaks away from the cluster and skips over to you. You’ve seen her at one or two parties. She points at you, grinning. “You’re Adam’s girlfriend.” Your throat catches. “Ex.” “Oh.” Her eyes twinkle. “I know a secret.” She leans closer. The smell of beer shoots up your nose. “Do you want to know?”
101	*Choice*	Don’t tell me.
102		Her gaze darts across the room. “Lex never thought you were good together.” She giggles. “I shouldn’t have said that.” She waves at a friend and disappears into the crowd.
103		You search for Lex, who is already engrossed in a game of Charades in the next room. Crossing your arms, you melt into the background and peer over the shoulder of an onlooker. A frat guy holds up a dry erase marker. “Alright, I’m looking for a volunteer brave enough to take on Lex, the Master of Charades herself.”
104	*Choice*	You raise your hand.
105		He points at you, gesturing with the marker. “Who better than Lex’s best friend? Come on up.” The crowd parts and you step up to the deck of cards. Selecting one, you scoff. Your prompt is a snake. The irony. You draw a messy squiggle with a forked tongue on the wall they’ve sacrificed for the game. Lex snaps her fingers. “Snake.” You confirm and the crowd cheers with excessive enthusiasm. “Yes, she’s wonderful.”

In the corresponding free recall (see below one subject’s recall), the participant recalled surrounding narrative content and one choice event (“I volunteer”) but did not recall the earlier brief choice prompt (“Don’t tell me.”). Under the event-based scoring procedure described in the manuscript, Event 101 therefore receives a recall score of 0, while Event 104 receives a score of 1. This illustrates how brief choice events can be missed in free recall even when adjacent

narrative content and other choice events are successfully recalled. When averaged across subjects and events, this design property naturally leads to lower absolute recall rates for choice events.

Event	***Choice***	***Subject Recall Texts***
99		There is beer pong
99		and the floors are sticky.
103		I walk over and see someone asking for another player for her game of Charades.
104	*Choice*	I volunteer
105		and I draw a picture of a snake with a forked tongue for my turn.
105		Lexi guesses it immediately.
105		The person who invited me said of course it's great you're Lexi's best friend to play this game.

The supplementary analysis (Supplementary S4) normalizing recall by word count was included solely to address this descriptive issue and not to assert a mechanistic relationship between text length and memory. Word count serves as a pragmatic proxy for differences in information load across event types under an event-based scoring approach, and the analysis shows that the apparent disadvantage for choice events is substantially reduced when these structural differences are taken into account.

To prevent misinterpretation, we have added a brief clarification to the legend of Figure 2A in the main manuscript, noting that choice events differ qualitatively from non-choice events and therefore should not be directly compared against each other:

> *“**Fig. 2| Recall ISC for the “shared story sections”. A,** Top: Average recall across all participants in each condition (Free, Yoked, Passive) for the 64 events in the shared story sections of the Romance story; the 15 choice events are indicated by grey bars. Bottom: Raster plot showing the raw data for each participant’s vector of recall performance (shaded = remembered, blank = forgotten). Note that choice events are qualitatively different from non-choice events in the current paradigm by design and therefore cannot be directly compared against each other. **B,** Left: Average within-condition Recall ISC for the 64 shared events. Right: Distribution plot from the permutation test comparing the true Recall ISC for the Free condition (blue dashed line) to 10,000 Recall ISC values from samples drawn from the Passive condition, where each Passive sample consisted of 18 unique story paths (green distribution). **C,** Same as B with Choice events excluded.”*

To provide further clarity, we also added to the supplement (S4. Recall for choice and non-choice events):

“Memory for choice events is significantly reduced compared to the other events (p < .001). The choice events in our paradigm differ in nature from the other (non-choice) narrative events—choice texts were presented in different fonts, sizes, styles, and served a different function from the non-choice events during the reading. Moreover, they describe specific actions that participants can select and thus are much shorter (around 5 words) than non-choice events (~40–50 words), which are usually extended narrative segments containing rich descriptive and action content (e.g., non-choice event: “One girl breaks away from the cluster and skips over to you. You’ve seen her at one or two parties. She points at you, grinning. “You’re Adam’s girlfriend.” Your throat catches. “Ex.” “Oh.” Her eyes twinkle. “I know a secret.” She leans closer. The smell of beer shoots up your nose. “Do you want to know?”; choice event: “Don’t tell me.”)

The overall less information contained in the choice events potentially rendered them less likely to be recalled in absolute terms. Given the differences in propositional density between non-choice and choice events, we may use word count to serve as a pragmatic proxy for information load within each scored event. When recall is normalized by the number of words in each event, we see the opposite pattern: participants recall choice events more often per word than normal story events. This was true across all agency conditions, even for the Passive condition.”

Finally, we would like to stress that the choice-versus-non-choice comparison is not central to the manuscript’s conclusions. All primary findings regarding agency-driven personalization of memory, reduced semantic predictability, and enhanced temporal binding remain unchanged when choice events are excluded from analysis.

We believe clarifying this design and scoring considerations will help readers more accurately interpret Fig. 2A and the supplementary analyses, and we appreciate the reviewer’s careful reading and the opportunity to clarify this point.

Comment3:
The network visualizations in Figure 3 are intriguing, but I could not find a clear explanation of what the shapes, layout, or edge weights represent, and how these networks were constructed. Please provide more detail on the methodology behind these graphs.

We appreciate this helpful request for clarification. The networks in Figure 3 are constructed from event-by-event relation matrices (semantic similarity or causal connection strength). Each node corresponds to a story event. Edges connect pairs of events if their similarity exceeds a fixed threshold of $\tau = 0.1$, with the edge thickness and color intensity proportional to the similarity weight. Node size is scaled by weighted degree centrality (sum of connected edge weights). Node positions are determined by a force-directed layout (Fruchterman–Reingold in MATLAB), which places more strongly connected events closer together.

I thank the authors for their clarifications. However, I remain unclear about several aspects of the network visualizations. First, do the spatial positions of the nodes carry specific meaning—that is, does the area where the networks are placed correspond to particular events? Second, what do the node colors (orange and blue) represent?
In addition, what do the color scales in the causal network matrices indicate (Figure 3B)? Please add a color mapping.

Thank you for the follow-up. Please see below for more detailed clarifications and revisions to the manuscript accordingly.

1) Do node spatial positions carry specific meaning (e.g., does a region of the plot correspond to particular events)?

No. The absolute spatial position of a node in the network plot does not correspond to a specific event index, story time, or any semantic category. Node coordinates are generated purely by a force-directed layout (Fruchterman–Reingold in MATLAB) applied to the thresholded similarity graph. This layout is used only to provide an intuitive visualization in which more strongly connected nodes tend to appear closer together, but the axes, orientation, and the overall placement of the network are otherwise arbitrary (e.g., the graph can rotate/flip without changing any underlying relationships). We now state this explicitly in the caption and Methods to prevent over-interpretation of the 2D placement.

For better clarity, we have added to the Methods section:

> *"Network visualization (Figure 3A-B) in matrix format showed the event-by-event pairwise Pearson similarity; those in the graph format (top right corners) are constructed from the similarity matrices for visualization purposes only: each node corresponds to a story event; edges connect pairs of events if their similarity exceeds a fixed threshold of τ = 0.1, with the edge thickness and color intensity proportional to the similarity weight; node size is scaled by weighted degree centrality (sum of connected edge weights); node positions are determined by a force-directed layout (Fruchterman–Reingold in MATLAB), which places more strongly connected events closer together. Note that the absolute 2D node coordinates, axes, and overall orientation carry no additional meaning and can rotate/flip without changing the underlying relationships."*

2) What do the node colors (orange and blue) represent?

Node color is not an analytic variable and does not encode event identity or event type. In our figure, the node colors are used simply to distinguish different example subjects' networks for visualization, i.e., example subject 1 networks have nodes in orange, while example subject 2 in blue. Each node still corresponds to a story event in that network.

> For better clarity, we have added to the Methods section:
>
> *"Node color is used solely as a visualization aid to distinguish example subject 1 (orange) from example subject 2 (blue)."*

3) What do the color scales in the causal network matrices indicate (Fig. 3B)?

In Fig. 3B, the matrix colormap encodes the pairwise causal relationship between event *i* and event *j*–the binary value in the corresponding cell of the causal relation matrix, with 1 being causally related (yellow), and 0 being unrelated (purple).

To improve readability, we have added a color legend to the figure as the reviewer suggested. We have also updated the caption to the figure to improve clarity:

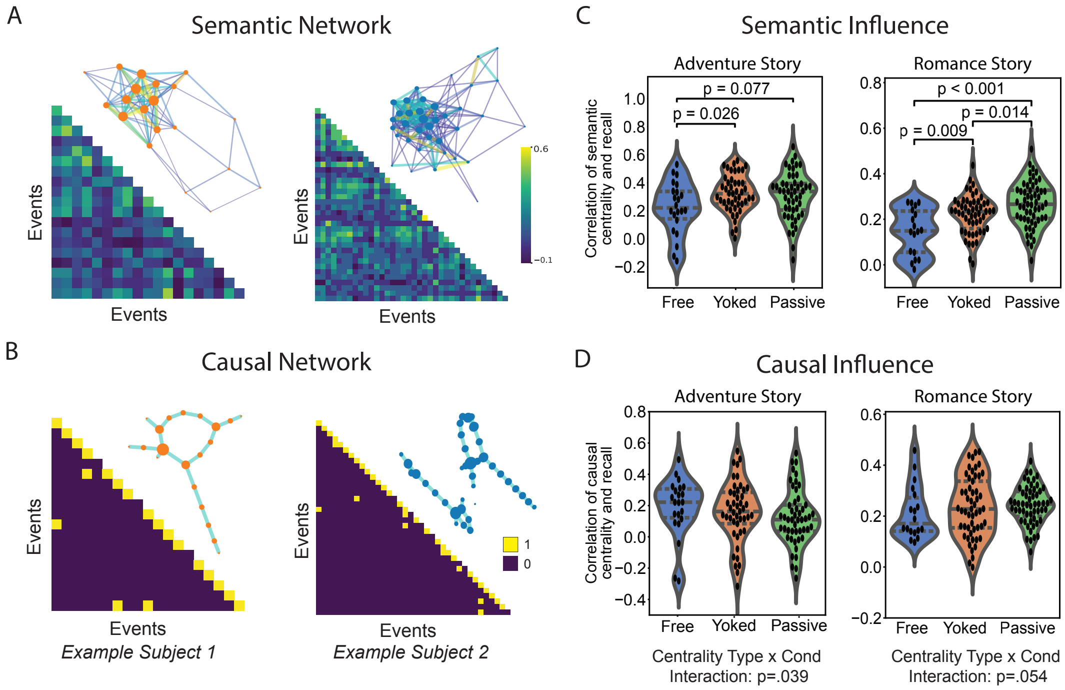

***Fig. 3| Semantic and causal centrality effects on memory. A**, Semantic similarity matrix showing the USE cosine similarity between pairs of events for example Adventure (left) and Romance (right) stories, and the corresponding semantic network plots for the same stories. Each node in the network graphs (top right corners) represents an event; edges (τ > 0.1) scale in thickness and color by similarity; node size reflects degree centrality. **B**, Causal matrix showing the human-judged causal connection (yellow: 1 = causally connected; purple: 0 = not causally connected) between pairs of events for example Adventure (left) and Romance (right) stories, and the corresponding causal network plots for the same stories (network graphs constructed using the same methods as in A). **C**, Distributions of semantic influence, i.e., the correlation between semantic centrality and recall performance, in the Adventure story (left) and the Romance story (right) in each condition. Each dot represents one participant. **D**, Distributions of causal influence, i.e., the correlation between causal centrality and recall performance, in the Adventure story (left) and the Romance story (right) in each condition. Each dot represents one participant.*

We hope these updates provided improvements to the clarity of the manuscript.

Line 780 – Figure 2B should be Figure 2A.

Thank you for bringing this to our attention—we have corrected it.

Reviewer #2 (Remarks to the Author):

All previously raised concerns have been appropriately addressed in the revisions.

> Thank you for your positive evaluation of the revision. We appreciate your time and thoughtful comments that helped improve our manuscript.

Reviewer #3 (Remarks to the Author):

I co-reviewed this manuscript with one of the reviewers who provided the listed reports. This is part of the Nature Communications initiative to facilitate training in peer review and to provide appropriate recognition for Early Career Researchers who co-review manuscripts.

> Thank you for your time and contribution to the review of our manuscript.

Reviewer #4 (Remarks to the Author):

I am satisfied with the authors' responses to my previous comments and appreciate the level of detail of the responses. I believe the manuscript has become much clearer and better streamlined. I have no further comments.

> Thank you for your thoughtful review. We appreciate the time and effort you invested in evaluating our work and helping improve the manuscript.

Reviewer #4 (Remarks on code availability):

I was able to run all_analyses.py and could reproduce the complete report (after providing my local path in base_path). I also randomly checked some of the individual run files and was able to create the corresponding output.

> Thank you for testing the code and verifying that you could reproduce the full report.
